# Unusual Vilasinin-Class Limonoids from *Trichilia rubescens*

**DOI:** 10.3390/molecules29030651

**Published:** 2024-01-30

**Authors:** Saidanxia Amuti, Yohei Saito, Shuichi Fukuyoshi, Katsunori Miyake, David J. Newman, Barry R. O’Keefe, Kuo-Hsiung Lee, Kyoko Nakagawa-Goto

**Affiliations:** 1 School of Pharmaceutical Sciences, College of Medical, Pharmaceutical and Health Sciences, Kanazawa University, Kanazawa 920-1192, Japan; saidanxia@stu.kanazawa-u.ac.jp (S.A.); saito-y@staff.kanazawa-u.ac.jp (Y.S.); fukuyosi@p.kanazawa-u.ac.jp (S.F.); 2 School of Pharmacy, Tokyo University of Pharmacy and Life Sciences, Hachioji 192-0392, Japan; miyake@toyaku.ac.jp; 3 Natural Products Branch, Developmental Therapeutics Program, Center of Cancer Research, Division of Cancer Treatment and Diagnosis, National Cancer Institute, Frederick, MD 21702-1201, USA; dn22a@nih.gov (D.J.N.); okeefeba@mail.nih.gov (B.R.O.); 4 Molecular Targets Program, Center for Cancer Research, Division of Cancer Treatment and Diagnosis, National Cancer Institute, Frederick, MD 21702-1201, USA; 5 Natural Products Research Laboratories, UNC Eshelman School of Pharmacy, University of North Carolina at Chapel Hill, Chapel Hill, NC 27599-7568, USA; 6 Chinese Medicine Research and Development Center, China Medical University and Hospital, 2 Yuh-Der Road, Taichung 40447, Taiwan

**Keywords:** *Trichilia rubescens*, vilasinin-class limonoid, chlorinated, rubescins K–R, antiproliferative activity

## Abstract

Eight vilasinin-class limonoids, including the unusually chlorinated rubescins K–M (**1**–**3**), the 2,3-epoxylated rubescin N (**4**), and rubescins O–R (**5**–**8**), were newly isolated from *Trichilia rubescens*. The structures of the isolated compounds were determined through spectroscopic and spectrometric analyses, as well as ECD calculations. The natural occurrence of chlorinated limonoids **1**–**3** was confirmed by chemical methods and HPLC analysis of a roughly fractionated portion of the plant extract. Eight selected limonoids, including previously known and new compounds, were evaluated for antiproliferative activity against five human tumor cell lines. All tested limonoids, except **8**, exhibited significant potency, with IC_50_ values of <10 μM; in particular, limonoid **14** strongly inhibited tumor cell growth, with IC_50_ values of 0.54–2.06 μM against all tumor cell lines, including multi-drug-resistant cells.

## 1. Introduction

Tropical rainforests are renowned for their extraordinary biodiversity, which is the result of intricate ecosystems, a diverse array of species, and extensive genetic diversity within those species. As part of ongoing research focused on the phytochemical exploration of tropical rainforests, we investigated *Trichilia rubescens* (Meliaceae), which displayed significant antiproliferative activity in an NCI-60 panel screening (Figure S1 in the Supplementary Materials).

**Figure 1:**
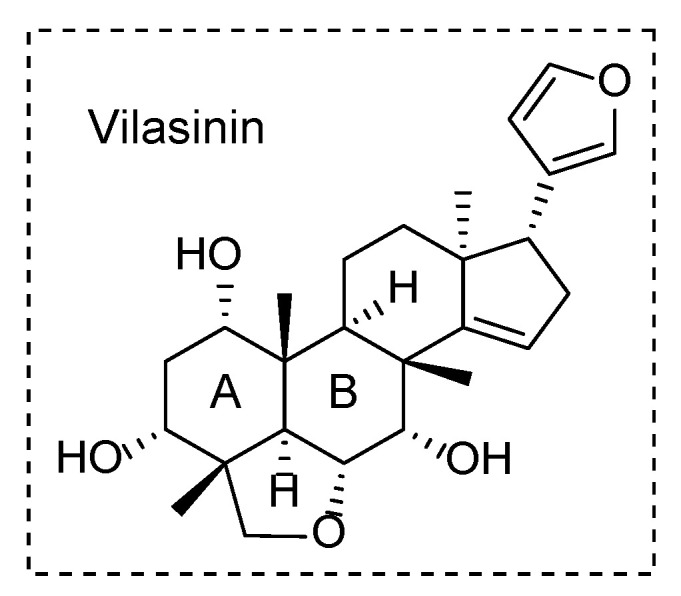
Structure of vilasinin, a ring-intact limonoid.

The family Meliaceae has approximately 50 genera and about 1400 species; however, only limited genera have been investigated phytochemically [1]. The genus *Trichilia*, commonly found in the tropical and subtropical regions of South America and Africa [2], is a well-studied genus known to produce various limonoids [3]. Limonoids are a structurally unique class of tetra-nor-triterpenes containing a furan ring and exhibit many biological activities, such as antitumor, anti-HIV, insecticidal, and anti-inflammatory activities [3,4]. Interestingly, limonoids have been isolated from specific restricted families, primarily Meliaceae and Rutaceae, with approximately 2700 meliaceous limonoids being isolated and identified to date [1]. Among the genera in the former family, the genus *Trichilia* yields ring-intact limonoids in a high 25% ratio, while other genera produce mostly rearranged limonoids [1]. Vilasinin is a ring-intact limonoid characterized by a tetrahydrofuran ring adjacent to the A and B rings (Figure 1). Currently, only 18 vilasinin-class limonoids have been reported from the genus *Trichilia*, with 15 isolated from *T. rubescens* [5,6,7,8,9,10,11,12].

**Figure 2:**
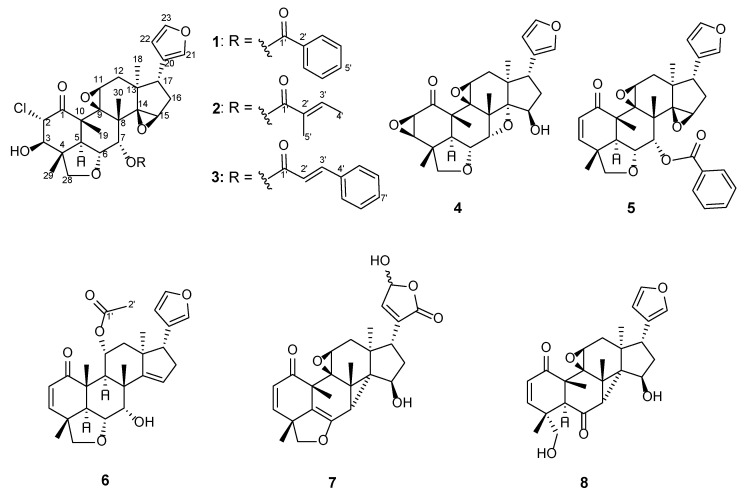
New vilasinin-class limonoids **1**–**8** isolated from *T. rubescens*.

**Figure 3:**
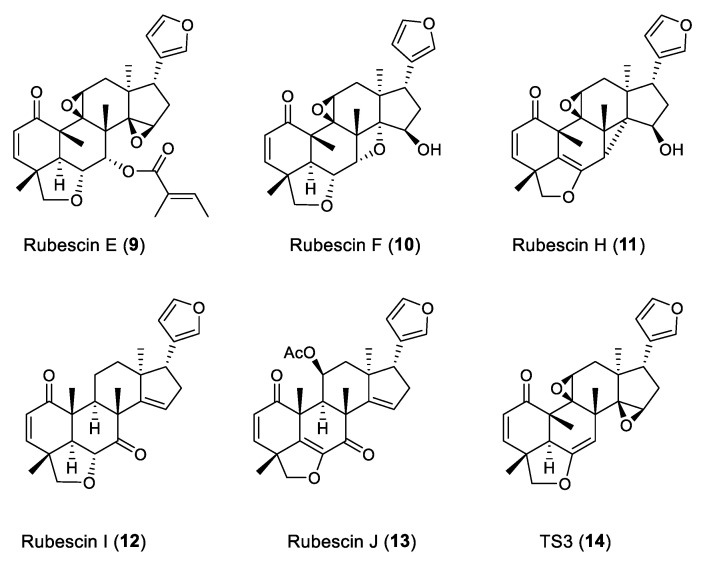
Known vilasinin-class limonoids **9**–**14** isolated from *T. rubescens* in this work.

In this study, extensive phytochemical research on *T. rubescens* led to the identification of eight new vilasinin-class limonoids, including the unusually chlorinated rubescins K–M (**1**–**3**), the 2,3-epoxylated rubescin N (**4**), and rubescins O–R (**5**–**8**) (Figure 2), along with six known vilasinin-class limonoids, rubescin E (**9**) [6], rubescin F (**10**) [7], rubescin H (**11**) [7], rubescin I (**12**) [8], rubescin J (**13**) [8], and TS3 (**14**) [9], three sesquiterpenes, guaianediol [13], alismol [14], and eudesm-4(15)-ene-1β,6α-diol [15], as well as two sterols, β-sitosterol and stigmasterol [16] (Figure 3). The natural occurrence of the chlorinated limonoids **1**–**3** was further confirmed using chemical methods and HPLC analysis. This paper deals with the isolation, structure elucidation, and evaluation of antiproliferative activity against human tumor cell lines of the isolated limonoids.

## 2. Results and Discussion

A 50% MeOH/CH_2_Cl_2_ extract (N047159) of the leaves of *T. rubescens* was partitioned with EtOAc and water. The EtOAc-soluble fraction was subjected to a series of chromatographic techniques using silica gel and octadecylsilica (ODS) gel via medium-pressure liquid chromatography (MPLC), column chromatography (CC), preparative TLC (pTLC), and HPLC to obtain pure new limonoids **1**–**8**, along with the known compounds **9**–**14**. The structures of all known compounds were identified by a comparison of their 1D NMRs with previously reported values [6,7,8,9].

**Figure 4:**
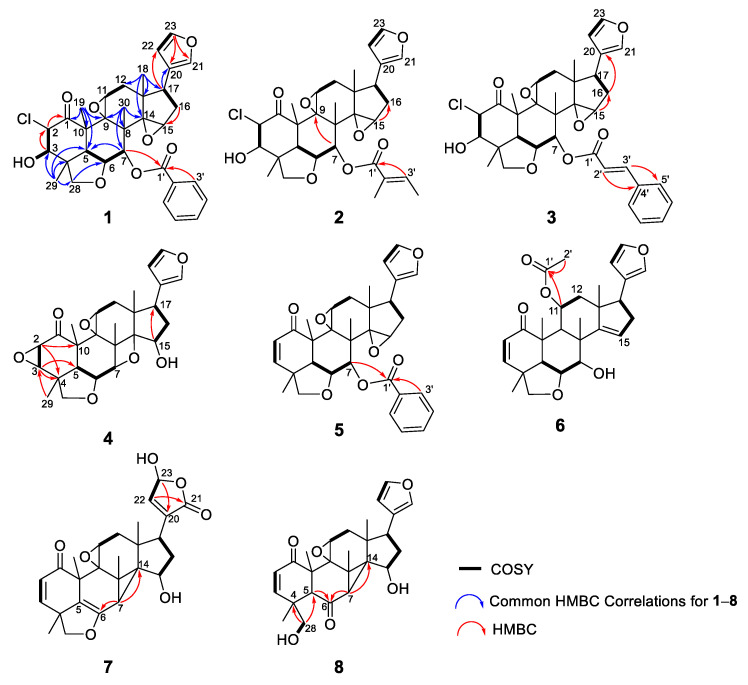
Selected ^1^H–^1^H COSY and HMBC correlations of new limonoids **1**–**8**.

**Figure 5:**
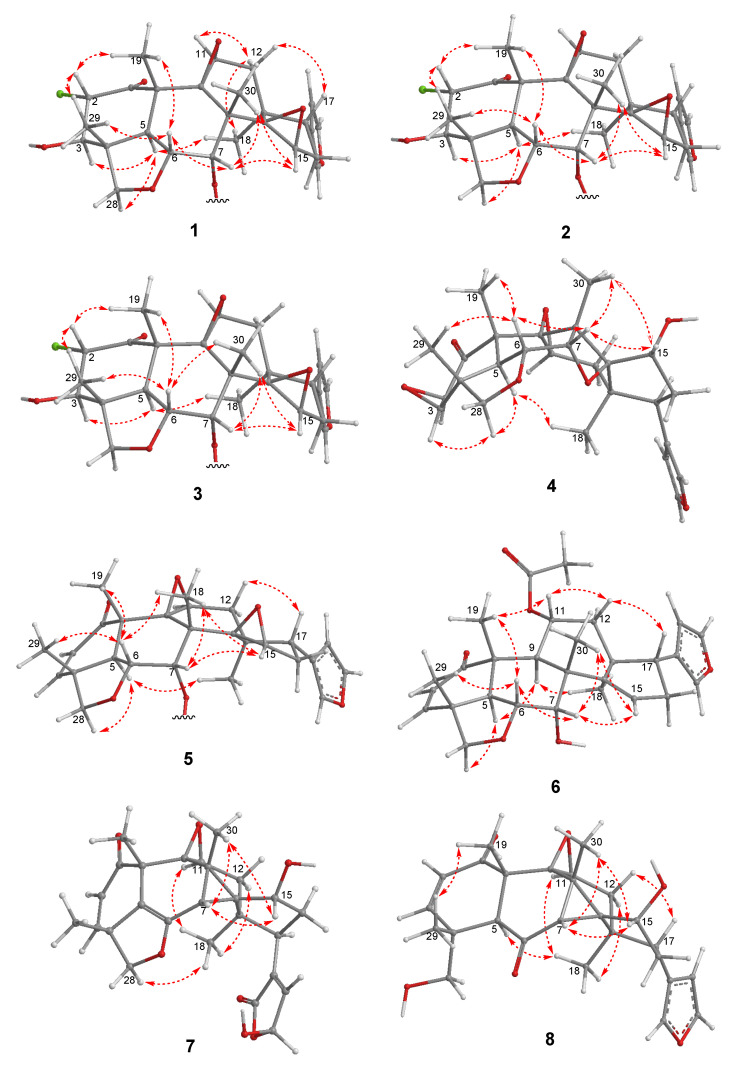
Selected NOESY correlations (red dashed arrows) of compounds **1**–**8**.

**Figure 6:**
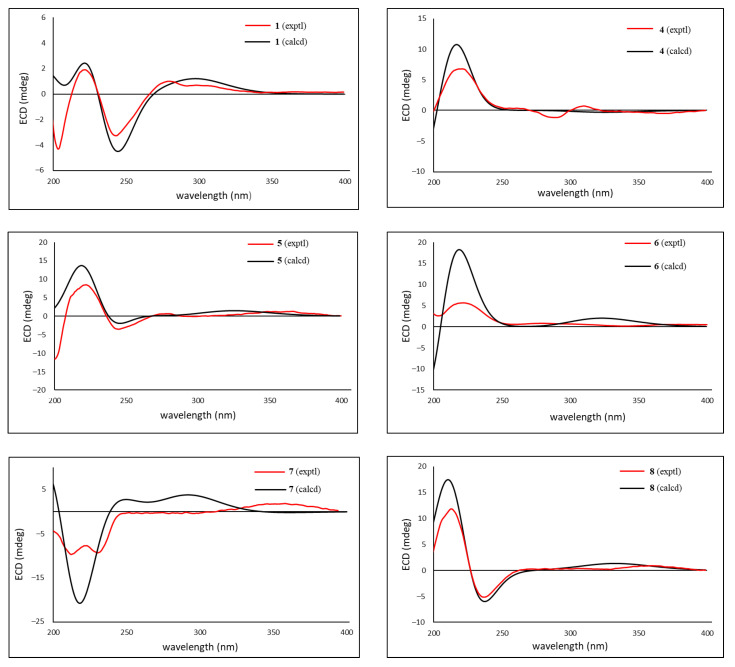
Experimental and calculated ECD spectra of compounds **1** and **4**–**8**.

Compound **1** was isolated as a colorless amorphous solid with a specific rotation of ${\left[\alpha \right]}_{D}^{21}\;$−22 (*c* 0.1, CHCl_3_). The HRAPCIMS spectrum of **1** showed protonated molecular peaks at *m*/*z* 595.2118 and 597.2100 [M+H]^+^ (calcd 595.2099 and 597.2069) in a 3:1 ratio, which suggested the presence of chlorine and a molecular formula of C_33_H_35_ClO_8_. The IR absorptions at 3257 and 1720 cm^−1^ implied the presence of hydroxy and carbonyl groups, respectively. The ^1^H NMR spectrum showed signals assignable to protons for a mono-substituted phenyl (δ_H_ 7.92, 7.62, 7.47), three olefinic, three methylene, eight methine, and four methyl groups (Table 1). The ^13^C NMR spectrum of **1** (Table 2) presented 33 peaks, including the signals for a ketone carbonyl (δ_C_ 199.4), an ester carbonyl (δ_C_ 165.9), four olefinic, and seven tertiary carbons. These signals were similar to those of TS2 (Figure S66 in the Supplementary Materials), a vilasinin-class limonoid with two β-epoxy groups at C-9, -11 and C-14, -15, a methacrylate at C-7, and an α,β-unsaturated ketone in ring-A, which was isolated previously from the leaves of *T. rubescens* [9]. However, the chemical shifts of C-2, C-3, and the ester moiety at C-7 in **1** differed significantly from those in TS2; therefore, compound **1** likely lacks a C-2, C-3 double bond and has a different ester group at C-7. The planar structure of **1** was further confirmed by 2D NMR experiments (Figure 4). ^1^H–^1^H COSY correlations were observed between OH-3/H-3, H-2/H-3, H-5/H-6/H-7, H-11/H-12, H-16/H-17, and H-22/H-23. The HMBC correlations from H_3_-19 to C-1 and H-2 to C-1 suggested a carbonyl at C-1, while the correlations from H_3_-29 to C-3 indicated a hydroxy group at C-3. A benzoyl ester was assigned at C-7, based on the HMBC correlations of H-7 and H-3′ with C-1′. The positions of the two epoxy groups at C-9, C-11 and C-14, C-15 were confirmed, based on multiple HMBC cross-peaks from H_3_-18, H_2_-16, and H_3_-30 to oxygenated carbons at C-14, C-15, and C-9, respectively. A pendant furan ring at C-17 was suggested by HMBC correlations from H-17 to C-20 and C-22, as well as from H-23 to C-20 and C-21. These data indicate that compound **1** has the same basic skeleton as TS2, but has a chlorine at C-2, a hydroxy at C-3, and a benzoate rather than methacrylate at C-7. The key NOESY correlations (Figure 5) from H-2 to H_3_-19/H_3_-29, H-6 to H-7/H_3_-19/H_3_-29, H-7 to H-15/H_3_-30, H-15 to H_3_-30, H-12α to H_3_-18/H-11, and H-12β to H-17 suggested α-orientations for the chlorine at C-2, the methyl at C-18, the ester carbonyl at C-7, and the furan ring at C-17; the latter three assignments were consistent with those of other related vilasinin-class limonoids, such as TS2 [9]. The absolute configuration of **1** was deduced by comparing the experimental and calculated ECD spectra (Figure 6). Thus, the structure of **1** (rubescin K) was elucidated as the first chlorinated vilasinin-class limonoid, with (2*S*, 3*R*, 4*R*, 5*S*, 6*R*, 7*S*, 8*S*, 9*S*, 10*S*, 11*S*, 13*S*, 14*R*, 15*R*, 17*S*) absolute configurations.

Compounds **2** (rubescin L) and **3** (rubescin M) were obtained as optically active colorless amorphous solids. Their HRAPCIMS spectra also indicated the presence of chlorine, and the protonated molecule peaks at *m*/*z* 573.2253/575.2231 [M+H]^+^ (3:1, calcd 573.2255/575.2226) for compound **2** and *m*/*z* 621.2248/623.2237 [M+H]^+^ (3:1, calcd 621.2255/623.2226) for compound **3** indicated the molecular formulae of C_31_H_37_ClO_8_ and C_35_H_37_ClO_8_, respectively. The similarities among the 1D (Table 1 and Table 2) and 2D (Figure 3 and Figure 4) NMR spectra of compounds **1**–**3** strongly suggested that all three compounds are 2-chloro-3-hydroxy vilasinin limonoids. The only differences were found in the signals assignable to the C-7 ester moiety. The ^13^C and ^1^H NMR spectra of compound **2** suggested a tigloyl group, with an olefinic methine and two methyl carbons, as well as an olefinic and two sets of methyl protons assigned to the ester moiety. Additionally, an HMBC correlation between H-3′ and C-1′ supported the presence of a tigloyl ester, and a good comparison was found with the NMR data of the related tigloyl limonoid, compound **9** [6]. The ester moiety of **3** was elucidated as a cinnamate, based on NMR observations of five phenyl methine and two olefinic protons, as well as HMBC correlations from olefinic protons H-2′ and H-3′ to phenyl carbons C-4′ and C-5′. Although limited isolations of related cinnamoyl limonoids from the Meliaceae have been reported, the comparisons of the NMR data for the ester moiety of compound **3** with those of other cinnamoyl limonoids, such as toosendansins E and F [17], 7-cinnamoyltoosendanin [18], and ohchininolide [19] and its derivatives [20], also supported the presence of a cinnamate at C-7 in **3**. Unfortunately, the instability of compounds **2** and **3** did not allow further investigation, such as with UV, IR, and ECD; however, all other data strongly supported that, like **1**, both compounds are interesting chlorinated limonoids.

Compound **4** was isolated as a colorless amorphous solid with an optical rotation of ${\left[\alpha \right]}_{D}^{21}\;$−13 (*c* 0.1, CHCl_3_). With a protonated molecular peak at *m*/*z* 455.2070 [M+H]^+^ (calcd. 455.2064) in its HRFABMS spectrum, the molecular formula of **4** is C_26_H_30_O_7_. The ^1^H and ^13^C NMR data (Table 3 and Table 4) of **4** were like those of **10** (Figure 3) [7], a vilasinin-class limonoid with an oxetane ring formed by an ether linkage between C-7 and C-14. However, the signals that were due to the olefinic protons (H-2/H-3) in **10** were replaced by signals for protons (δ_H_ 3.15 and δ_H_ 7.57) on oxygenated carbons in **4**, with a ^1^H–^1^H COSY correlation between the two protons. The ^13^C NMR signals for C-2 and C-3 in **4** (δc 51.9 and δc 60.4) were shielded from those in **10** (δc 131.3 and δc 152.7) [7]. The above data together with HMBC correlations between H-2 and C-4/C-10, H-3 and C-4/C-5, and H3-29 and C-3 indicated the presence of an epoxy ring at C-2/C-3. The relative configurations of **4** were established from a NOESY experiment (Figure 5). The β-orientation of the 2,3-epoxy moiety was suggested by the key correlations of H-3/H_2_-28α and H_2_-28α/H-5. The NOESY correlations from H-7 to H-15/H_3_-30 and from H-15 to H_3_-30 also indicated a β-orientation for OH-15. A strong NOE correlation was observed between β-oriented H-7 and α-oriented H-15; both protons are pseudo-equatorial, with a proton-proton distance of 2.4 Å. The same relative configurations were also supported by a comparison with all NMR data for compound **10** [7]. The absolute configuration was supplied from the calculated and experimental ECD spectra (Figure 6). Thus, compound **4** (rubescin N) is (2*R*, 3*R*, 4*R*, 5*S*, 6*R*, 7*S*, 8*S*, 9*S*, 10*S*, 11*S*, 13*S*, 14*S*, 15*R*, 17*S*)-2,3-epoxyrubescin N, the first reported 2,3-epoxylated vilasinin-class limonoid. Furthermore, it is the second reported vilasinin-class limonoid with an oxetane ring [7], although several other classes of oxetane limonoids have been isolated [21,22,23,24]. As a biogenetic oxetane formation has been proposed previously [21,23], compound **4** might be produced from TS1 (Figure S66), which was also isolated from the same plant [9] through 2,3-epoxidation and the intramolecular nucleophilic attack of OH-7 on C-14 to open the epoxy ring.

Compound **5** (rubescin O) has a molecular formula of C_33_H_34_O_7_, as indicated by HRFABMS. Its ^1^H and ^13^C NMR signal patterns were nearly identical to those of compound **1**, suggesting a vilasinin skeleton for **5** with two epoxy moieties at C-9 (δ_C_ 64.6)/C-11 (δ_C_ 60.4/δ_H_ 3.98) and C-14 (δ_C_ 68.3)/C-15 (δ_C_ 55.2/δ_H_ 3.50), a furan ring at C-17 (δ_C_ 38.7/δ_H_ 2.47), and a benzoate at C-7 (δ_C_ 75.5/δ_H_ 5.68). However, significantly different chemical shifts were observed at C-2 (δ_C_ 131.3/δ_H_ 5.99) and C-3 (δ_C_ 151.7/δ_H_ 7.10) (Table 3 and Table 4), which implied the presence of an olefin creating an α,β-unsaturated ketone with C-1 (δ_C_ 200.3). This assignment was confirmed via comparisons with the NMR data of the related limonoids with an α,β-unsaturated ketone, such as compound **9** (Figure 3) [6]. All 2D NMR, including COSY, HMBC, and NOESY, supported the planar structure and the relative configurations of **5** (Figure 4 and Figure 5). The absolute configurations of **5** (4*R*, 5*S*, 6*R*, 7*S*, 8*S*, 9*S*, 10*S*, 11*S*, 13*S*, 14*R*, 15*R*, 17*S*) were deduced via a comparison of the experimental and calculated ECD spectra (Figure 6).

The molecular formula of compound **6** (rubescin P) was elucidated as C_28_H_34_O_6_, based on HRFABMS. The 1D NMR data for **6** were related to those of rubescins C [5] and J [8], which are vilasinin-class limonoids with an acetate at C-11. The major difference was the chemical shift at C-7 (δ_C_ 75.4) and the appearance of a proton at δ_H_ 4.09, suggesting the presence of a hydroxy group at C-7 (Table 3 and Table 4). The HMBC correlations of H-11/C-1′ and H_3_-2′/C-1′ (Figure 4) and the NOESY correlations between H-11 and H_3_-19/H-12β provided further support for an α-oriented acetoxy group at C-11 (Figure 5). The NOESY correlations of H-7/H-6/H_3_-30 indicated that the C-7 hydroxy group was β-oriented. The absolute configurations of (4*R*, 5*S*, 6*R*, 7*S*, 8*R*, 9*S*, 10*R*, 11*S*, 13*S*, 17*R*) were deduced from the calculated and experimental ECD spectrum (Figure 6).

Compound **7** (rubescin Q) has a molecular formula of C_26_H_28_O_7_, as suggested by HRFABMS. The 1D and 2D NMR spectroscopic data (Table 3 and Table 4) of **7** indicated a comparable structure to that of **11** [7], in which a cyclopropane ring is formed between C-7, C-8, and C-14. However, the pendant furan ring found at C-17 in **11** was not present in **7**. Instead, a 5-hydroxy-2-oxo-dihydrofuran ring was present in **7**, based on the HMBC correlations (Figure 4) from H-17 to C-20 (δ_C_ 138.8/138.9) and from H-23 to C-20 (δ_C_ 138.8/138.9) and H-22 to C-21 (δ_C_ 171.3/171.0), together with a ^1^H–^1^H COSY correlation between H-22 and H-23. Also, the related chemical shifts were identical to those of trichirubine A, which has the same substituent at C-17 [10]. The 1:1 pairwise signals observed in the ^1^H and ^13^C NMR spectra indicated that **7** was a diastereomixture caused by the epimerization of a hemiacetal moiety at C-23. The same observation was also made with rubescin G [7] and trichirubine A [10]. Based on the relative configurations revealed by NOESY correlations (Figure 4), the NOESY cross-peaks of H-7/H_3_-30/H-15 confirmed that a hydroxy group at C-15 and a proton at C-7 were β-oriented. The rigid conformation around the cyclopropane ring necessarily allowed the construction of the *R* configuration at C-14. The calculated and experimental ECD (Figure 6) elucidated the absolute configuration of **7** as (4*R*, 7*S*, 8*S*, 9*S*, 10*S*, 11*S*, 13*S*, 14*R*, 15*R*, 17*R*).

HRFABMS suggested a molecular formula of C_26_H_30_O_6_ for compound **8** (rubescin R). A comparison of the NMR data (Table 3 and Table 4) of **8** with those of compound **11** [7] (Figure 3) showed overall similarity, except at C-5, C-6, C-7, C-28, and C-29. The most significant differences were the shielded C-5 (δ_c_ 55.4) and the deshielded C-6 (δc 207.1) and C-7 (δc 44.4) in **8** compared with **11**, together with the appearance of a second hydroxy proton (δ_H_ 1.64, 28-OH) and an aliphatic methine proton (δ_H_ 3.67, H-5). These data suggested the absence of a C-5, C-6 double bond, the presence of a carbonyl at C-6, and the cleavage of the ether linkage between C-6 and C-28. The HMBC correlations of H-5 and H-7 with C-6 also supported the presence of a carbonyl at C-6 (δ_C_ 207.1), while the shielded carbon signal for C-28 (δ_C_ 70.3), the COSY connectivity of H_2_-28/OH-28, and the HMBC cross-peaks of H_2_-28 to C-4/C-5 indicated the presence of a hydroxymethyl moiety at C-4. While a NOESY analysis (Figure 5) confirmed the relative configuration of **8**, an ECD analysis (Figure 6) and the negative specific rotation value determined that **8** and **11** have the same absolute configuration (4*R*, 5*S*, 7*S*, 8*S*, 9*S*, 10*S*, 11*S*, 13*S*, 14*R*, 15*R*, 17*R*). All the above data indicate that **8** is a seco-vilasinin-class limonoid and is a possible biosynthetic intermediate of **11**.

**Figure 7:**
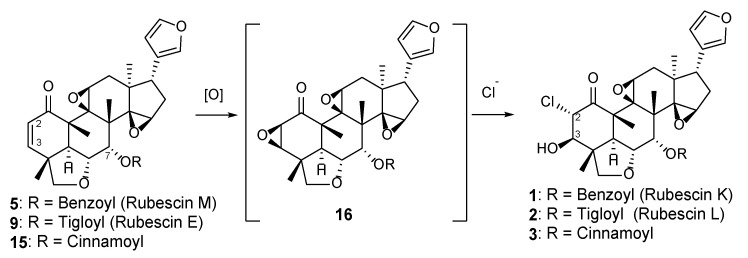
Possible biosynthetic pathway of chlorinated vilasinin-class limonoids.

A possible biosynthetic pathway to the chlorinated limonoids **1**–**3** could involve the epoxidation of related limonoids, such as **5**, **9**, and **15**, which contain an α,β-unsaturated ketone in ring-A to give the tri-epoxy **16**, followed by chlorination at C-2 (Figure 7). Limonoids **5** and **9** were isolated in this study. While halogenation is thought to primarily be a final biosynthetic step, limited evidence has indicated that halogenation can also be followed by subsequent steps that lead to other biosynthetic intermediates [25]. Accordingly, there is a slight possibility that an epoxy group might be produced by the attack of a hydroxy group and the removal of a neighboring chlorine atom.

Halogenated natural products are produced mainly by marine organisms living in halogen-rich environments, or by microorganisms such as algae, cyanobacteria, and fungus [26,27,28,29,30,31]. Although rarely isolated from terrestrial plants, several plant-derived natural products containing halogen, usually chlorine, have been reported [32,33,34,35]. However, some of these compounds could be artifacts that are produced when halogenated solvents are used. Since this study is the first to report chlorinated limonoids and halogen-containing natural products from the family Meliaceae, further investigation was needed to determine whether they occur naturally in the plant extract.

**Figure 8:**
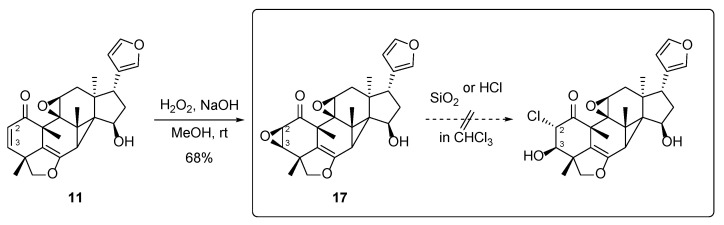
Investigation of the possibility of an artifact using a model substrate **11**.

Chlorination of the related β-2,3-epoxy limonoid **16** (Figure 7) could happen during the isolation process, for example, with the use of CHCl_3_ or CH_2_Cl_2_ under acidic conditions, such as on silica gel, or unexpectedly, by contamination with HCl. To investigate the possibility that the isolated chlorinated limonoids might be artifacts, the model substrate **17** was prepared as a biosynthetic precursor mimic of **1**. The known limonoid **11**, which was isolated in sufficient quantities in this study, was epoxidized via a general condition using H_2_O_2_ to give **17** at a 68% yield (Figure 8) [36]. The β-orientation of the 2,3-epoxide was confirmed by a NOESY correlation between H-3 and H-28α, which was also seen with **4**. The treatment of **17** with excess silica gel in CHCl_3_ for 12 h produced no reaction; only the starting material was recovered. This observation suggested a low probability of chlorination occurring during silica gel column chromatography with a chlorinated solvent. The treatment of **17** with dried HCl on silica gel in CHCl_3_ gave a complex inseparable mixture rather than a chlorinated product [37]. The ^1^H-NMR spectrum of the mixture showed no characteristic peaks at H-2 and H-3 corresponding to the 2-chloro-3-hydroxy segment in **1**. This result indicated that the chlorination of a related epoxide was unlikely, even after unexpected contamination with HCl.

To further confirm that the isolated chlorinated limonoids are natural products, LC/MS analysis was carried out on the initial fraction 7b containing **1**, which was roughly twice separated from the original extract using silica gel CC. The peak with the exact mass for an authentic sample of **1** was observed within a range of 4.3–4.5 min, and the same peak was detected in fraction 7b at the same retention time (Figures S64 and S65 in the Supplementary Materials).

Based on the results of the above chemical methods and LC/MS analysis, it is highly possible that chlorinated limonoids **1**–**3** occur naturally in *T. rubescens*.

Eight selected vilasinin-class limonoids (**1**, **5**, **6**, **8**, **9**, **11**, **13**, and **14**) were evaluated for their antiproliferative activities against human tumor lines (HTCLs), A549 (lung adenocarcinoma), MDA-MB-231 (triple-negative breast cancer), MCF-7 (HER2-negative), KB (HeLa derivative), and KB-VIN [P-gp overexpressing multidrug-resistant (MDR) KB subline] (Table 5). All the tested limonoids, except **8**, showed significant activity, with IC_50_ values of 0.54–8.46 μM, even against the KB-VIN MDR cell line, which suggested that these limonoids are not P-gp substrates. In particular, compound **14** exhibited the highest potency against all HTCLs, including the MDR tumor cell line. Compounds **9** and **14** have also been reported to effectively decrease the viability of hepatoma cells at TC_50_ concentrations ranging from 5 to 10 μM. [38]. Comparing the results of the current study, these two compounds show potential value as anticancer drugs. The chlorine-containing limonoid **1** showed slightly selective inhibition against the MCF-7 cell line (IC_50_ 1.34 μM) compared with the other tested HTCLs (IC_50_ 2.53–5.72 μM). Compounds **1** and **5** showed similar activity, indicating that the 2,3-double bond is not very important for this activity. The comparison of **5**, **9**, and **14** suggested that the function group at C-7 has an insignificant effect on this activity, although compound **14,** with a 6,7-double bond, displayed slightly more potent activity than other compounds. Since compound **8** did not exhibit significant antiproliferative activity (IC_50_ > 40 μM), the tetrahydrofuran ring, which is formed by an ether linkage between C-6 and C-28, might be important for the antiproliferative activity of this compound type [39].

## 3. Materials and Methods

### 3.1. General Experimental Procedures

Optical rotations were determined using a JASCO P-2200 digital polarimeter (JASCO, Tokyo, Japan). The UV spectra were measured with a Thermo Scientific Genesys 50 spectrometer (Thermo Scientific, Waltham, MA, USA). ECD spectra were measured with a JASCO J-820 spectrometer. Infrared spectra (IR) were recorded with a Shimadzu IRSpirit FT-IR spectrometer (Shimadzu, Kyoto, Japan) with a QATR-S single reflection ATR accessory, using neat samples. The NMR spectra were obtained via the JEOL JMN-ECA600 and JMN-ECS400 NMR spectrometers (JEOL, Akishima, Japan), with tetramethylsilane as an internal standard; chemical shifts are stated as *δ* values. HRMS data were recorded on a JMS-700 MStation (FAB) mass spectrometer. MPLC analysis was performed with C_18_ cartridges (Sfär C18 D, Biotage, Charlotte, NC, USA). Preparative HPLC analysis was conducted on a GL Science recycling system using an InertSustain C_18_ column (5 μm; 20 × 250 mm) (GL Sciences, Tokyo, Japan). LC/MS analysis was recorded with a Shimadzu LCMS-9030 connected to an Imtakt Cadenza CX-C18 (2 × 50 mm; 3 μm) (Imtakt, Kyoto, Japan).

### 3.2. Plant Material

A 50% CH_2_Cl_2_/MeOH extract of *T. rubescens* leaves (N047159) was provided by the NCI Natural Products Branch (Developmental Therapeutics Branch, Frederick, MD, USA) as reported previously [40]. The plants were collected in the Central Africa Republic by J. M. Fay in August 1988 and were identified by the taxonomist R. Gereau.

### 3.3. Extraction and Isolation

The extract N047159 (10.0 g) was partitioned between H_2_O and EtOAc to obtain H_2_O-soluble and EtOAc-soluble portions. The EtOAc-soluble portion (5.2 g) was subjected to silica gel CC using gradient mixed solvents [*n*-hexane/EtOAc (9:1, 8:2, 2:1, 0:1), CH_3_OH] as eluents to afford eight fractions (frs. 1–8). Fr. 7 was separated by silica gel CC (CHCl_3_ /MeOH, 1:0–9:1) to give six fractions (frs. 7a–f). Fr. 7a was loaded on ODS-MPLC (MeOH/H_2_O, 7:3) to give eight subfractions (frs. 7a1–8). Frs. 7a3 and 7a4 were crystallized from MeOH, to give compounds **11** (28.5 mg) and **14** (47.8 mg), respectively. Further separation of Fr. 7a5 by silica gel CC (CHCl_3_/MeOH, 100:0–95:5), following ODS-HPLC (CH_3_CN/H_2_O, 65:35), afforded compounds **13** (24.4 mg), **1** (9.9 mg), and **6** (2.0 mg). Fr. 7a6 was subjected to silica gel CC (CHCl_3_/MeOH, 100:0–95:5) to give four fractions (frs. 7a6a–d). Frs. 7a6a and 7a6b were purified by ODS-HPLC (MeCN/H_2_O, 65:35) to yield compounds **5** (3.2 mg) and **9** (1.2 mg). The sequential CC of fr. 7a7 with silica gel CC (*n*-hexane/EtOAc, 4:1–6:4) and pTLC (*n*-hexane/EtOAc, 4:1) afforded compound **12** (0.8 mg).

Fr. 7b was subjected to ODS-MPLC (MeOH/H_2_O, 70:30) to yield five fractions (frs. 7b1–5). Fr. 7b1 was separated by silica gel CC (CHCl_3_/MeOH, 100:0–95:5) to afford seven fractions (frs. 7b1a–g). Sequential silica gel chromatography (*n*-hexane/EtOAc, 4:1–1:1 and CHCl_3_/MeOH, 100:0–99:1) afforded compound **4** (0.7 mg). Fr. 7b2 was separated by silica gel CC (CHCl_3_/MeOH, 100:0–95:5) and ODS-HPLC (MeCN/H_2_O, 60:40) to give compounds **1** (3.1 mg) and **2** (2.1 mg). Fr. 7b3 was subjected to silica gel CC (CHCl_3_/MeOH, 100:0–95:5) to give four fractions (frs. 7b3a–d). Purification of fr. 7b3b by ODS-HPLC (MeCN/H_2_O, 60:40) furnished compound **1** (18.1 mg). Fr. 7b4 was separated by silica gel CC (CHCl_3_/MeOH, 100:0–97:3, followed by toluene/EtOAc, 1:0–4:1) and ODS-HPLC (MeCN/H_2_O, 60:40) to obtain compound **3** (3.4 mg).

Fr. 7c was separated by ODS-MPLC (MeOH/H_2_O, 60:40) to yield four fractions (frs. 7c1–4). Fr. 7c2 was applied to ODS-MPLC (MeOH/H_2_O, 40:60) to furnish eight fractions (frs. 7c2a–h). Fr. 7c2b was further separated by ODS-HPLC (MeCN/H_2_O, 40:60) and silica gel CC (*n*-hexane/EtOAc, 2:1–1:2, toluene/EtOAc, 3:2–0:1 and then *n*-hexane/EtOAc, 2:1–1:2) to give compound **10** (0.7 mg).

Fr. 7d was applied to ODS-MPLC (MeOH/H_2_O, 40:60) to give three fractions (frs. 7d1–3). Fr. 7d2 was separated by ODS–MPLC (MeCN/H_2_O, 40:60), silica gel CC (CHCl_3_/MeOH, 100:0–97:3), and ODS-HPLC (MeCN/H_2_O, 40:60), following silica gel CC (*n*-hexane/EtOAc, 6:4–4:6), to give compound **8** (3.3 mg).

Fr. 7e was loaded on ODS-MPLC (MeOH/H_2_O, 70:30 and MeOH/H_2_O, 50:50) following a silica gel CC (CHCl_3_/MeOH,100:0–95:5) to give compound **7** (1.2 mg).

Fr. 4 was separated by ODS-MPLC (MeOH/H_2_O, 70:30 and MeCN/H_2_O, 70:30) to yield guaianediol (27.5 mg). Fr. 5 was fractionated by silica gel CC (CHCl_3_/MeOH, 100:0–95:5 and *n*-hexane/acetone, 9:1–0:1) to afford a mixture of β-sitosterol and stigmasterol (13.2 mg). Fr. 6 was loaded onto a Sephadex LH-20 column (MeOH/H_2_O, 60:40–90:10), silica gel CC (*n*-hexane/CH_2_Cl_2_/EtOAc, 5:4:1, 5:3:2, 0:0:1 and CHCl_3_/MeOH, 100:0–98:2) following ODS-HPLC (MeCN/H_2_O, 40:60) to yield eudesm-4(15)-ene-1β,6α-diol (1.2 mg).

*Rubescin K* (**1**): Colorless amorphous solid; ${\left[\alpha \right]}_{D}^{21}\;$−22 (*c* 0.1, CHCl_3_); UV (MeOH) λ_max_ (logε) 226 (4.04); ECD (MeCN) λ_max_ (Δε) 222 (+1.92); IR (neat) ν_max_ 3857, 2941, 2870, 1720, 1278 cm^−1^, HRAPCIMS *m*/*z* 595.2118 [M+H]^+^ (calcd for C_33_H_36_ClO_8_, 595.2099); ^1^H and ^13^C NMR data (Table 1 and Table 2).

*Rubescin L* (**2**): Colorless amorphous solid; ${\left[\alpha \right]}_{D}^{21}\;$−25 (*c* 0.1, CHCl_3_); HRAPCIMS *m/z* 573.2253 [M+H]^+^ (calcd for C_31_H_38_ClO_8_, 573.2255), ^1^H and ^13^C NMR data (Table 1 and Table 2).

*Rubescin M* (**3**): Colorless amorphous solid; ${\left[\alpha \right]}_{D}^{21}\;$−20 (*c* 0.1, CHCl_3_); HRAPCIMS *m/z* 621.2248 [M+H]^+^ (calcd for C_35_H_38_ClO_8_, 621.2255), ^1^H and ^13^C NMR data (Table 1 and Table 2).

*Rubescin N* (**4**): Colorless amorphous solid; ${\left[\alpha \right]}_{D}^{20}\;$−13 (*c* 0.1, CHCl_3_); UV (MeOH) λ_max_ (logε) 202 (3.85); ECD (MeCN) λ_max_ (Δε) 221 (+6.81); IR (neat) ν_max_ 2925, 1713, 1453, 1378 cm^−1^; HRFABMS *m*/*z* 455.2064 [M+H]^+^ (calcd for C_26_H_31_O_7_, 455.2070); ^1^H and ^13^C NMR data (Table 3 and Table 4).

*Rubescin O* (**5**): Colorless amorphous solid; ${\left[\alpha \right]}_{D}^{23}\;$+16 (*c* 0.1, CHCl_3_); UV (MeOH) λ_max_ (logε) 226 (4.14); ECD (MeCN) λ_max_ (Δε) 222 (+8.50); IR (neat) ν_max_ 2939, 2923, 1720, 1675, 1453 cm^−1^; HRFABMS *m*/*z* 543.2378 [M+H]^+^ (calcd for C_33_H_35_O_7_, 543.2383); ^1^H and ^13^C NMR data (Table 3 and Table 4).

*Rubescin P* (**6**): Colorless amorphous solid; ${\left[\alpha \right]}_{D}^{23}\;$+33 (*c* 0.1, CHCl_3_); UV (MeOH) λ_max_ (logε) 204 (4.02); ECD (MeCN) λ_max_ (Δε) 222 (+5.69); IR (neat) ν_max_ 2926, 1732, 1675 cm^−1^; HRFABMS *m*/*z* 467.2440, [M+H]^+^ (calcd for C_28_H_35_O_6_, 467.2434); ^1^H and ^13^C NMR data (Table 3 and Table 4).

*Rubescin Q* (**7**): Colorless amorphous solid; ${\left[\alpha \right]}_{D}^{20}\;$−81 (*c* 0.1, CHCl_3_); UV (MeOH) λ_max_ (logε) 218 (3.98); ECD (MeCN) λ_max_ (Δε) 223 (–7.77); IR (neat) ν_max_ 3468, 2930, 1752, 1723, 1700, 1655 cm^−1^; HRFABMS *m/z* 453.1895 [M+H]^+^ (calcd for C_26_H_29_O_7_, 453.1913); ^1^H and ^13^C NMR data (Table 3 and Table 4).

*Rubescin R* (**8**): Colorless amorphous solid; ${\left[\alpha \right]}_{D}^{20}$ −29 (*c* 0.1, CHCl_3_); UV (MeOH) λ_max_ (logε) 216 (4.14); ECD (MeCN) λ_max_ (Δε) 220 (+11.89); IR (neat) ν_max_ 3468, 2926, 1672 1665 cm^−1^; HRFABMS *m*/*z* 439.2109 [M+H]^+^ (calcd for C_26_H_31_O_6_, 439.2121); ^1^H and ^13^C NMR data (Table 3 and Table 4).

Preparation of compound **17**: 5% NaOH (0.2 mL) and 30% H_2_O_2_ (0.099 mL) were added to compound **11** (17.0 mg) in MeOH (1.0 mL) and the solution was stirred at room temperature for 11.5 h. The completion of the reaction was confirmed with TLC. The reaction mixture was quenched with the 1M HCl (20 mL). The resulting mixture was then extracted 3 times with EtOAc. The organic layer was dried over Na_2_SO_4_, filtered, and evaporated under vacuum [36]. The crude product was purified via silica gel CC (*n*-hexane: EtOAc; 1:1) to yield compound **17** as a colorless amorphous solid (12.0 mg).

*2,3-Epoxyrubescin H* (**17**): Colorless amorphous solid; ${\left[\alpha \right]}_{D}^{20}$ −281 (*c* 0.1, CHCl_3_), UV (MeOH) λ_max_ (logε) 206 (4.12); IR (neat) ν_max_ 2925, 1713, 1453, 1378 cm^−1^, HRFABMS *m*/*z* 437.1962 [M+H]^+^ (calcd for C_26_H_29_O_6_, 437.1964), ^1^H NMR (600 MHz, CDCl_3_) 7.38 (1H, t, *J* = 1.6 Hz, H-23), 7.22 (1H, dd, *J* = 1.6, 0.7 Hz, H-21), 6.27 (1H, dd, *J* = 1.6, 0.7 Hz, H-22), 4.42 (1H, d, *J* = 9.3 Hz, H-28a), 4.27 (1H, d, *J* = 9.3 Hz, H-28b), 3.9 (1H, brs, H-15), 3.4 (1H, dd, *J* = 13.8, 5.2 Hz, H-17), 3.32 (1H, d, *J* = 4.0 Hz, H-3), 3.29 (1H, d, *J* = 4.0 Hz, H-2), 2.85 (1H, dd, *J* = 6.5, 4.0 Hz, H-11), 2.21 (1H, dd, *J* = 13.6, 6.5 Hz, H-12a), 2.16 (1H, ddd, *J* = 13.8, 12.5, 3.2 Hz, H-16a), 1.95 (1H, ddd, *J* = 12.5, 5.2, 1.0 Hz, H-16b), 1.65 (3H, s, H-30), 1.62 (1H, s, H-7), 1.54 (3H, s, H-19), 1.53 (1H, brs, OH-15), 1.36 (1H, dd, *J* = 13.6, 4.0 Hz, H-12b), 1.31 (3H, s, H-29), 0.8 (3H, s, H-18). ^13^C NMR (150 MHz, CDCl_3_) 205.1 (C-1), 151.9 (C-6), 142.8 (C-23), 139.5 (C-21), 124.5 (C-20), 115.6 (C-5), 111.2 (C-22), 80.1 (C-28), 79.8 (C-15), 63.1 (C-3), 62.0 (C-9), 59.3 (C-11), 57.1 (C-2), 51.3 (C-14), 48.9 (C-10), 45.9 (C-4), 45.5 (C-17), 43.7 (C-13), 40.9 (C-12), 38.7 (C-16), 29.5 (C-7), 23.7 (C-8), 23.1 (C-19), 23.08 (C-29), 19.5 (C-18), 19.1 (C-30).

### 3.4. Calculation of ECD Spectra

Preliminary conformational analyses of all compounds, except compound **4**, were performed by CONFLEX9 with the MMFF94 force field. Spaltan20 was used for the preliminary conformational analysis of compound **4**. The obtained conformers were further optimized in MeOH by the density functional theory (DFT) method, with the B3LYP functional and 6–31(d) basis set. The ECD spectrum was calculated via the time-dependent DFT (TDDFT) method, using the CAM-B3LYP functional and TZVP basis set. The calculation was performed using the conformers within 2 kcal/mol predicted in MeOH. The solvent effect was introduced by the conductor-like polarizable continuum model (CPCM). The DFT optimization and TDDFT-ECD calculation were accomplished by Gaussian16 (Gaussian, Inc., Wallingford, CT, USA). The calculated spectrum was displayed using GaussView 6.1, with the peak half-width at half-height being 0.333 eV. The Boltzmann-averaged spectrum at 298.15 K was calculated using Excel 2016 (Microsoft Co., Redmond, WA, USA). The calculations were re-optimized according to the literature [41].

### 3.5. Antiproliferative Activity Assay

A549, KB, MDA-MB-231 and MCF-7 were obtained from the Lineberger comprehensive Cancer Center (UNC-CH, NC) or from ATCC (Manassas, VA, USA). KB-VIN was a generous gift of Professor Y.-C. Cheng (Yale University, New Haven, CT, USA). We confirmed our KB and KB-VIN are identical to AV-3 (ATCC number, CCL-21) as a HeLa (cervical carcinoma) derivative by short tandem repeat (STR) profiling. The antiproliferative activity was examined via an SRB assay, as described previously [42]. Briefly, freshly trypsinized cell suspensions were seeded in 96-well microtiter plates at densities of 4000–11,000 cells per well for each compound. The attached cells were fixed in 10% trichloroacetic acid and stained with 0.04% SRB after culturing for 72 h. The absorbance at 515 nm was measured using a microplate reader (Spark 10M, Tecan, Zurich, Switzerland) with SparkControl software version 2.3 (Tecan) after solubilizing the bound dye with 10 mM Tris base. The mean IC_50_ was determined as the average from at least three independent experiments of duplication for an assay and similar determinations.

## 4. Conclusions

The phytochemical investigation of a 50% MeOH/CH_2_Cl_2_ extract from the leaves of a tropical rainforest plant, *T. rubescens,* led to the isolation of 14 vilasinin-class limonoids, including the new rubescins K–R (**1**–**8**) and known rubescins E, F, and H–J (**9**–**13**), as well as TS3 (**14**), together with three sesquiterpenes and two sterols. An extensive analysis of isolated compounds revealed that rubescins K–M (**1**–**3**) were unusually chlorinated limonoids and rubescin N (**4**) was the first 2,3-epoxylated vilasinin-class limonoid. The natural occurrence of chlorinated limonoids was further confirmed using chemical methods and LC/MS analysis. 

The isolated vilasinin-type limonoids **1**, **5**, **6**, **8**, **9**, **11**, **13**, and **14** were evaluated for their growth-inhibitory effects against five human tumor cell lines, including a multidrug-resistant cell line, KB-VIN. All the tested limonoids, except for compound **8**, exhibited significant activity, with IC_50_ values of 0.54–8.46 μM against all tested tumor cell lines, including KB-VIN. Compound **14** showed the highest inhibitory activity, while chlorinated limonoid **1** demonstrated slightly selective inhibition against the MCF-7 cell line. The preliminary structure–activity relationship study indicated that the tetrahydrofuran ring formed by C4–6 and C-28, which is characteristic of the vilasinin-class limonoid, might be important for this activity.

## Figures and Tables

**Table 1:** ^1^H NMR spectroscopic data (600 MHz, CDCl_3_) of chlorinated limonoids **1**–**3**.

Position	1	2		3	
	δ_H_ (*J* in Hz)	δ_H_ *(J* in Hz)		δ_H_ (*J* in Hz)	
2	4.74, d (10.1)	4.72, d (10.3)		4.70, d (10.1)	
3	3.87, d (10.1)	3.77, d (10.3)		3.84, d (10.1)	
5	2.54, d (11.9)	2.38, d (11.9)		2.43, d (11.9)	
6	4.59, dd (11.9, 3.8)	4.51, dd (11.9, 3.8)		4.54, dd (11.9, 3.9)	
7	5.66, d (3.8)	5.47, d (3.8)		5.48, d (3.8)	
11	4.68, dd (6.9, 1.4)	4.65, dd (6.9, 1.4)		4.65, dd (6.8, 1.3)	
12a	1.97, dd (13.7, 6.9)	1.98, dd (13.3, 6.9)		2.03, dd (13.5, 6.8)	
12b	1.84, dd (13.7, 1.4)	1.83, overlap		1.87, d (13.5)	
15	3.53, brs ^a^	3.46, brs ^a^		3.45, brs ^a^	
16a	2.07, ddd (13.3, 6.5, 1.0)	2.11, ddd (13.4, 6.5, 0.7)		2.12, ddd (13.4, 6.4, 1.0)	
16b	1.42, dd (13.3, 11.3)	1.54, overlap		1.59, dd (13.4, 11.3)	
17	2.47, dd (11.3, 6.5)	2.51, dd (11.3, 6.5)		2.53, dd (11.3, 6.4)	
18	0.61, s	0.62, s		0.77, s	
19	1.54, s	1.50, s		1.51, s	
21	6.88, dd (1.6, 0.9)	7.03, brs		7.01, dd (1.6, 0.9)	
22	5.87, dd (1.6, 0.9)	6.07, brs		6.07, dd (1.6, 0.9)	
23	7.24, t (1.6)	7.35, t (1.7)		7.31, t (1.6)	
28a	3.85, d (7.3)	3.91, d (7.9)		3.92, d (7.8)	
28b	3.48, d (7.3)	3.52, d (7.9)		3.54, d (7.8)	
29	1.40, s	1.39, s		1.38, s	
30	1.26, s	1.20, s		1.22, s	
OH-3	2.68, brs ^a^	2.61, brs ^a^		2.58, brs ^a^	
Ester moiety at C-7					
position	*δ*_H_ (*J* in Hz)	position	*δ*_H_ (*J* in Hz)	position	*δ*_H_ (*J* in Hz)
2′		2′		2′	6.37, d (15.9)
3′/7′	7.92, dd (8.3, 1.3)	3′	6.72, dd (7.2, 1.4)	3′	7.79, d (15.9)
4′/6′	7.47, dd (8.3, 8.3)	4′	1.83, d (7.2)	4′	-
5′	7.62, m	5′	1.86, brs	5′/9′	7.53, dd (7.8, 2.2)
				6′/8′	7.44, m
				7′	7.43, m

^a^ Broad singlet.

**Table 2:** ^13^C NMR spectroscopic data (150 MHz, CDCl_3_) of chlorinated limonoids **1**–**3**.

Position	1	2		3	
	*δ*_C_	*δ*_C_		*δ*_C_	
1	199.4	199.3		199.5	
2	68.8	68.7		68.7	
3	79.4	79.5		79.2	
4	45.4	45.3		45.2	
5	48.2	48.1		47.8	
6	71.3	71.3		71.3	
7	74.9	74.1		74.1	
8	45.1	45.2		45.0	
9	64.9	64.9		64.9	
10	50.1	50.1		50.0	
11	60.2	60.2		60.2	
12	35.1	35.3		35.2	
13	41.1	41.1		41.2	
14	68.4	68.3		68.4	
15	55.6	55.6		55.5	
16	31.1	31.1		31.1	
17	38.6	38.8		38.7	
18	21.7	21.4		18.1	
19	18.1	18.2		21.2	
20	122.7	123.0		122.9	
21	139.3	139.4		139.4	
22	110.8	110.9		110.9	
23	142.8	142.9		143.0	
28	83.0	82.9		83.0	
29	14.3	14.4		14.3	
30	23.0	22.8		22.9	
Ester moiety at C-7					
position	*δ*_C_	position	*δ*_C_	position	*δ*_C_
1′	165.9	1′	167.2	1′	165.9
2′	129.8	2′	128.7	2′	116.7
3′/7′	128.7	3′	138.5	3′	147.1
4′/6′	129.6	4′	14.6	4′	133.9
5′	133.6	5′	12.7	5′/9′	128.3
				6′/8′	129.1
				7′	130.9

**Table 3:** ^1^H NMR spectroscopic data (600MHz, CDCl_3_) of compounds **4**–**8**.

Position	4	5	6	7	8
	*δ*_H_ (*J* in Hz)	*δ*_H_ (*J* in Hz)	*δ*_H_ (*J* in Hz)	*δ*_H_ (*J* in Hz)	*δ*_H_ (*J* in Hz)
2	3.15, d (2.8)	5.99, d (9.6)	5.87, d (9.6)	5.85, d (9.8)	5.90, d (10.3)
3	3.57, d (2.8)	7.10, d (9.6)	6.94, d (9.6)	6.68, d (9.8)	6.64, d (10.3)
5	3.12, d (12.6)	3.05, d (12.7)	2.66, d (12.4)		3.67, s
6	4.00, dd (12.6, 4.1)	4.49, dd (12.7, 4.1)	4.34, dd (12.4, 3.1)		
7	5.02, d (4.1)	5.68, d (4.1)	4.09, d (3.1)	1.66, s	2.00, s
9			2.44, d (3.7)		
11	3.18, dd (6.5, 4.7)	3.98, dd (6.5, 1.4)	6.23, dd (8.0, 3.7)	2.69, dd (6.3, 4.3)	3.04, dd (6.5, 3.6)
12a	2.21, dd (13.8, 6.5)	1.90, dd (13.6, 6.5)	2.50, dd (14.6, 8.0)	2.60, dd (13.5, 6.3)	2.27, dd (13.8, 6.5)
12b	1.31, overlap	1.85, d (13.6)	1.64, dd (14.6, 3.7)	1.47, dd (13.5, 4.3)	1.51, dd (13.8, 3.6)
15	4.66, brs ^a^	3.50, brs ^a^	5.67, brs ^a^	3.90, brs ^a^	4.00, brs ^a^
16a	2.08, ddd (13.9, 13.0, 2.9)	2.07, dd (13.2, 6.5)	2.53, ddd (15.6, 11.3, 1.7)	2.17, ddd (13.0, 11.6, 3.6)	2.20, ddd (13.8, 12.6, 3.4)
16b	1.85, ddd (13.0, 5.3, 1.0)	1.41, dd (13.2, 11.3)	2.45, ddd (15.6, 7.6, 3.4)	1.95/1.93, dd (11.6, 5.6)	1.95, ddd (12.6, 4.8, 0.9)
17	3.12, dd (13.9, 5.3)	2.47, dd (11.3, 6.5)	2.89, dd (11.3, 7.6)	3.34/3.32, dd (13.0, 5.6)	3.43, dd (13.8, 4.8)
18	0.76, s	0.64, s	0.82, s	0.66/0.63, s	0.67, s
19	1.48, s	1.46, s	1.35, s	1.64/1.63, s	1.67, s
21	7.17, dd (1.6, 0.8)	6.84, brs ^a^	7.25, brs ^a^		7.21, dd (1.7, 0.8)
22	6.20, dd (1.6, 0.8)	5.87, brs ^a^	6.30, brs ^a^	6.85, s	6.23, dd (1.7, 0.8)
23	7.37, t (1.6)	7.22, t (1.7)	7.37, t (1.7)	6.12, brs	7.21, t (1.7)
28a	3.96, d (7.7)	3.70, d (7.4)	3.79, d (7.2)	4.29, d (9.0)	4.20, dd (10.2, 7.1)
28b	3.92, d (7.7)	3.54, d (7.4)	3.62, d (7.2)	4.07/4.05, d (9.0)	3.45, dd (10.2, 7.1)
29	1.26, s	1.35, s	1.36, s	1.36, s	1.20, s
30	1.31, s	1.27, s	1.46, s	1.68/1.67, s	1.67, s
OH-7			2.09, s		
OH-15	1.42, d (2.9)			1.55, overlap	1.56, d (2.8)
OH-28					1.64, d (7.1)
2′			2.04, s		
3′/7′		7.94, dd (7.9, 1.4)			
4′/6′		7.46, dd (7.9, 7.9)			
5′		7.59, m			

^a^ Broad singlet.

**Table 4:** ^13^C NMR spectroscopic data (150MHz, CDCl_3_) of compounds **4**–**8**.

Position	4	5	6	7 ^a^	8
	*δ*_C_	*δ*_C_	*δ*_C_	*δ*_C_	*δ*_C_
1	202.4	200.3	202.1	197.1	196.7
2	51.9	131.3	130.0	128.54/128.53	127.6
3	60.4	151.7	150.8	152.4/152.3	155.4
4	39.0	42.8	41.9	44.8	42.1
5	51.8	50.8	48.0	118.2	55.4
6	71.8	71.3	73.8	149.7	207.1
7	80.0	75.5	75.4	29.1	44.4
8	47.9	44.8	45.9	22.7	29.3
9	61.8	64.6	39.4	60.5	61.3
10	47.8	47.6	47.3	48.2	49.3
11	59.4	60.4	71.0	58.7	58.1
12	37.2	35.3	44.2	40.9/40.7	39.9
13	45.6	41.4	45.5	43.8/43.7	43.9
14	96.1	68.3	158.4	51.4, 51.3	55.2
15	76.1	55.2	121.6	79.0	80.3
16	37.1	31.1	34.3	37.5/37.4	38.6
17	43.0	38.7	52.3	45.0/44.9	46.0
18	19.9	21.8	22.5	19.4	22.3
19	15.9	17.4	16.3	21.9	21.0
20	123.9	122.8	124.1	138.8/138.9	124.1
21	139.5	139.3	139.8	171.3/171.0	139.6
22	111.1	110.9	110.9	144.5/144.9	110.9
23	142.9	142.7	142.8	96.7/96.3	143.1
28	79.4	79.7	80.1	81.4	70.3
29	16.8	21.1	20.1	28.1	17.4
30	21.6	22.8	27.8	19.2	18.9
1′		166.1	169.7		
2′		128.6	21.8		
3′/7′		129.9			
4′/6′		129.8			
5′		133.4			

^a^ 1:1 Diastereomixture.

**Table 5:** Antiproliferative activity of the selected limonoids.

	Cell Lines ^a^ (IC_50_ μM)				
Compounds	A549	MDA-MB-231	MCF-7	KB	KB-VIN
**1**	2.53	5.72	1.34	3.38	4.02
**5**	3.17	5.45	2.85	4.48	2.09
**6**	3.39	17.6	4.56	6.83	8.07
**8**	37.4	>40	>40	>40	>40
**9**	2.14	5.88	0.96	3.68	4.35
**11**	1.60	20.9	8.46	6.83	8.09
**13**	1.42	6.76	8.07	4.38	3.28
**14**	0.56	2.06	0.75	0.57	0.54
PXL ^b^ (nM)	2.57	12.41	2.71	6.76	2939.33

^a^ A549: Lung adenocarcinoma, MDA-MB-231: triple-negative breast cancer (ER-/PR-/HER2-), MCF-7: HER2-negative, KB: originally isolated from an epidermoid carcinoma of the nasopharynx (contaminated HeLa cell), KB-VIN: multidrug-resistant KB subline. ^b^ Paclitaxel.

## Data Availability

The data presented in this study are available on request from the corresponding author.

## Supplementary Materials

The following supporting information can be downloaded at: https://www.mdpi.com/article/10.3390/molecules29030651/s1, Figure S1. NCI-60 Human tumor cell line assay data for the crude organic extract of *T. rubescens* (N047159). Figure S2. ^1^H NMR spectrum of compound **1** in CDCl_3_ at 600 MHz. Figure S3. ^13^C NMR spectrum of compound **1** in CDCl_3_ at 150 MHz. Figure S4. HMQC spectrum of compound **1** in CDCl_3_ at 600 MHz. Figure S5. HMBC spectrum of compound **1** in CDCl_3_ at 600 MHz. Figure S6. COSY spectrum of compound **1** in CDCl_3_ at 600 MHz. Figure S7. NOESY spectrum of compound **1** in CDCl_3_ at 600 MHz. Figure S8. UV spectrum of compound **1** in MeOH. Figure S9. IR spectrum of compound **1**. Figure S10. ^1^H NMR spectrum of compound **2** in CDCl_3_ at 600 MHz. Figure S11. ^13^C NMR spectrum of compound **2** in CDCl3 at 150 MHz. Figure S12. HMQC spectrum of compound **2** in CDCl_3_ at 600 MHz. Figure S13. HMBC spectrum of compound **2** in CDCl_3_ at 600 MHz. Figure S14. COSY spectrum of compound **2** in CDCl_3_ at 600 MHz. Figure S15. NOESY spectrum of compound **2** in CDCl_3_ at 600 MHz. Figure S16. ^1^H NMR spectrum of compound **3** in CDCl_3_ at 600 MHz. Figure S17. ^13^C NMR spectrum of compound **3** in CDCl_3_ at 150 MHz. Figure S18. HMQC spectrum of compound **3** in CDCl_3_ at 600 MHz. Figure S19. HMBC spectrum of compound **3** in CDCl_3_ at 600 MHz. Figure S20. COSY spectrum of compound **3** in CDCl_3_ at 600 MHz. Figure S21. NOESY spectrum of compound **3** in CDCl_3_ at 600 MHz. Figure S22. ^1^H NMR spectrum of compound **4** in CDCl_3_ at 600 MHz. Figure S23. ^13^C NMR spectrum of compound **4** in CDCl_3_ at 150 MHz. Figure S24. HMQC spectrum of compound **4** in CDCl_3_ at 600 MHz. Figure S25. HMBC spectrum of compound **4** in CDCl_3_ at 600 MHz. Figure S26. COSY spectrum of compound **4** in CDCl_3_ at 600 MHz. Figure S27. NOESY spectrum of compound **4** in CDCl_3_ at 600 MHz. Figure S28. UV spectrum of compound **4** in MeOH. Figure S29. IR spectrum of compound **4**. Figure S30. ^1^H NMR spectrum of compound **5** in CDCl_3_ at 600 MHz. Figure S31. ^13^C NMR spectrum of compound **5** in CDCl_3_ at 150 MHz. Figure S32. HMQC spectrum of compound **5** in CDCl_3_ at 600 MHz. Figure S33. HMBC spectrum of compound **5** in CDCl_3_ at 600 MHz. Figure S34. COSY spectrum of compound **5** in CDCl_3_ at 600 MHz. Figure S35. NOESY spectrum of compound **5** in CDCl_3_ at 600 MHz. Figure S36. UV spectrum of compound **5** in MeOH. Figure S37. IR spectrum of compound **5**. Figure S38. ^1^H NMR spectrum of compound **6** in CDCl_3_ at 600 MHz. Figure S39.^13^C NMR spectrum of compound **6** in CDCl_3_ at 150 MHz. Figure S40. HMQC spectrum of compound **6** in CDCl_3_ at 600 MHz. Figure S41. HMBC spectrum of compound **6** in CDCl_3_ at 600 MHz. Figure S42. COSY spectrum of compound **6** in CDCl_3_ at 600 MHz. Figure S43. NOESY spectrum of compound **6** in CDCl_3_ at 600 MHz. Figure S44. UV spectrum of compound **6** in MeOH. Figure S45. IR spectrum of compound **6**. Figure S46. ^1^H NMR spectrum of compound **7** in CDCl_3_ at 600 MHz. Figure S47. ^13^C NMR spectrum of compound **7** in CDCl_3_ at 150 MHz. Figure S48. HMQC spectrum of compound **7** in CDCl_3_ at 600 MHz. Figure S49. HMBC spectrum of compound **7** in CDCl_3_ at 600 MHz. Figure S50. COSY spectrum of compound **7** in CDCl_3_ at 600 MHz. Figure S51. NOESY spectrum of compound **7** in CDCl_3_ at 600 MHz. Figure S52. UV spectrum of compound **7** in MeOH. Figure S53. IR spectrum of compound **7**. Figure S54. ^1^H NMR spectrum of compound **8** in CDCl_3_ at 600 MHz. Figure S55.^13^C NMR spectrum of compound **8** in CDCl_3_ at 150 MHz. Figure S56. HMQC spectrum of compound **8** in CDCl_3_ at 600 MHz. Figure S57. HMBC spectrum of compound **8** in CDCl_3_ at 600 MHz. Figure S58. COSY spectrum of compound **8** in CDCl_3_ at 600 MHz. Figure S59. NOESY spectrum of compound **8** in CDCl_3_ at 600 MHz. Figure S60. UV spectrum of compound **8** in MeOH. Figure S61. IR spectrum of compound **8**. Figure S62. ^1^H NMR spectrum of compound **17** in CDCl_3_ at 600 MHz. Figure S63.^13^C NMR spectrum of compound **17** in CDCl_3_ at 150 MHz. Figure S64. LC/MS analysis of compound **1**. Figure S65. LC/MS analysis of fraction 7.2. Figure S66. Structures of TS1 and TS2.
