# Supplementary material for: Unusual Vilasinin-Class Limonoids from Trichilia rubescens
Source: Molecules. 2024 Jan 30;29(3):651. doi: 10.3390/molecules29030651 (PMC10856392; doi:10.3390/molecules29030651)
Supplement: Supplementary file 1 [file molecules-29-00651-s001.zip › molecules-2835374-supplementary.pdf]

# Supporting Information

## Unusual Vilasinin-Class Limonoids from *Trichilia rubescens*

**Saidanxia Amuti [1], Yohei Saito [1], Shuichi Fukuyoshi [1], Katsunori Miyake [2], David J. Newman [3], Barry R. O'Keefe [3,4], Kuo-Hsiung Lee [5,6,†] and Kyoko Nakagawa-Goto [1,5,*]**

[1] School of Pharmaceutical Sciences, College of Medical, Pharmaceutical and Health Sciences, Kanazawa University, Kanazawa 920-1192, Japan; saidanxia@stu.kanazawa-u.ac.jp (S.A.); saito-y@staff.kanazawa-u.ac.jp (Y.S.); fukuyosi@p.kanazawa-u.ac.jp (S.F.)
[2] School of Pharmacy, Tokyo University of Pharmacy and Life Sciences, Hachioji 192-0392, Japan; miyake@toyaku.ac.jp
[3] Natural Products Branch, Developmental Therapeutics Program, Center of Cancer Research, Division of Cancer Treatment and Diagnosis, National Cancer Institute, Frederick, MD 21702-1201, USA; dn22a@nih.gov (D.J.N.); okeefeba@mail.nih.gov (B.R.O.)
[4] Molecular Targets Program, Center for Cancer Research, Division of Cancer Treatment and Diagnosis, National Cancer Institute, Frederick, MD 21702-1201, USA
[5] Natural Products Research Laboratories, UNC Eshelman School of Pharmacy, University of North Carolina at Chapel Hill, Chapel Hill, NC 27599-7568, USA; khlee@unc.edu
[6] Chinese Medicine Research and Development Center, China Medical University and Hospital, 2 Yuh-Der Road, Taichung 40447, Taiwan
* Correspondence: kngoto@p.kanazawa-u.ac.jp
† Deceased 24 October 2021.

**List of Contents**

Figure S1. NCI-60 Human tumor cell line assay data for the crude organic extract of *T. rubescens* (N047159)........S1
Figure S2. $^{1}H$ NMR spectrum of compound **1** in $CDCl_3$ at 600MHz........S2
Figure S3. $^{13}C$ NMR spectrum of compound **1** in $CDCl_3$ at 150MHz........S2
Figure S4. HMQC spectrum of compound **1** in CDCl3 at 600MHz........S3
Figure S5. HMBC spectrum of compound **1** in $CDCl_3$ at 600MHz........S3
Figure S6. COSY spectrum of compound **1** in $CDCl_3$ at 600MHz........S4
Figure S7. NOESY spectrum of compound **1** in $CDCl_3$ at 600MHz........S4
Figure S8. UV spectrum of compound **1** in MeOH ........S5
Figure S9. IR spectrum of compound **1** ........S5

Figure S10. $^{1}H$ NMR spectrum of compound **2** in $CDCl_3$ at 600MHz........S6
Figure S11.$^{13}C$ NMR spectrum of compound **2** in $CDCl_3$ at 150MHz........S6
Figure S12. HMQC spectrum of compound **2** in $CDCl_3$ at 600MHz........S7
Figure S13. HMBC spectrum of compound **2** in $CDCl_3$ at 600MHz........S7
Figure S14. COSY spectrum of compound **2** in $CDCl_3$ at 600MHz........S8
Figure S15. NOESY spectrum of compound **2** in $CDCl_3$ at 600MHz........S8

Figure S16. $^{1}H$ NMR spectrum of compound **3** in $CDCl_3$ at 600MHz........S9
Figure S17.$^{13}C$ NMR spectrum of compound **3** in $CDCl_3$ at 150MHz........S9
Figure S18. HMQC spectrum of compound **3** in $CDCl_3$ at 600MHz........S10
Figure S19. HMBC spectrum of compound **3** in $CDCl_3$ at 600MHz........S10
Figure S20. COSY spectrum of compound **3** in $CDCl_3$ at 600MHz........S11
Figure S21. NOESY spectrum of compound **3** in $CDCl_3$ at 600MHz........S11

Figure S22. $^{1}H$ NMR spectrum of compound **4** in $CDCl_3$ at 600MHz........S12
Figure S23.$^{13}C$ NMR spectrum of compound **4** in $CDCl_3$ at 150MHz........S12
Figure S24. HMQC spectrum of compound **4** in $CDCl_3$ at 600MHz........S13
Figure S25. HMBC spectrum of compound **4** in $CDCl_3$ at 600MHz........S13
Figure S26. COSY spectrum of compound **4** in $CDCl_3$ at 600MHz........S14
Figure S27. NOESY spectrum of compound **4** in $CDCl_3$ at 600MHz........S14
Figure S28. UV spectrum of compound **4** in MeOH ........S15
Figure S29. IR spectrum of compound **4** ........S15

Figure S30. $^{1}H$ NMR spectrum of compound **5** in $CDCl_3$ at 600MHz........S16
Figure S31.$^{13}C$ NMR spectrum of compound **5** in $CDCl_3$ at 150MHz........S16
Figure S32. HMQC spectrum of compound **5** in $CDCl_3$ at 600MHz........S17
Figure S33. HMBC spectrum of compound **5** in $CDCl_3$ at 600MHz........S17
Figure S34. COSY spectrum of compound **5** in $CDCl_3$ at 600MHz........S18
Figure S35. NOESY spectrum of compound **5** in $CDCl_3$ at 600MHz........S18
Figure S36. UV spectrum of compound **5** in MeOH ........S19
Figure S37. IR spectrum of compound **5** ........S19

Figure S38. $^{1}$H NMR spectrum of compound **6** in $CDCl_3$ at 600MHz....................S20
Figure S39.$^{13}$C NMR spectrum of compound **6** in $CDCl_3$ at 150MHz....................S20
Figure S40. HMQC spectrum of compound **6** in $CDCl_3$ at 600MHz......................S21
Figure S41. HMBC spectrum of compound **6** in $CDCl_3$ at 600MHz......................S21
Figure S42. COSY spectrum of compound **6** in $CDCl_3$ at 600MHz.......................S22
Figure S43. NOESY spectrum of compound **6** in $CDCl_3$ at 600MHz....................S22
Figure S44. UV spectrum of compound **6** in MeOH ..............................................S23
Figure S45. IR spectrum of compound **6** ..............................................................S23

Figure S46. $^{1}$H NMR spectrum of compound **7** in $CDCl_3$ at 600MHz....................S24
Figure S47.$^{13}$C NMR spectrum of compound **7** in $CDCl_3$ at 150MHz....................S24
Figure S48. HMQC spectrum of compound **7** in $CDCl_3$ at 600MHz......................S25
Figure S49. HMBC spectrum of compound **7** in $CDCl_3$ at 600MHz......................S25
Figure S50. COSY spectrum of compound **7** in $CDCl_3$ at 600MHz.......................S26
Figure S51. NOESY spectrum of compound **7** in $CDCl_3$ at 600MHz....................S26
Figure S52. UV spectrum of compound **7** in MeOH ..............................................S27
Figure S53. IR spectrum of compound **7** ..............................................................S27

Figure S54. $^{1}$H NMR spectrum of compound **8** in $CDCl_3$ at 600MHz....................S28
Figure S55.$^{13}$C NMR spectrum of compound **8** in $CDCl_3$ at 150MHz....................S28
Figure S56. HMQC spectrum of compound **8** in $CDCl_3$ at 600MHz......................S29
Figure S57. HMBC spectrum of compound **8** in $CDCl_3$ at 600MHz......................S29
Figure S58. COSY spectrum of compound **8** in $CDCl_3$ at 600MHz.......................S30
Figure S59. NOESY spectrum of compound **8** in $CDCl_3$ at 600MHz....................S30
Figure S60. UV spectrum of compound **8** in MeOH ..............................................S31
Figure S61. IR spectrum of compound **8** ..............................................................S31

Figure S62. $^{1}$H NMR spectrum of compound **17** in $CDCl_3$ at 600 MHz..................S32
Figure S63.$^{13}$C NMR spectrum of compound **17** in $CDCl_3$ at 150 MHz..................S32

Figure S64. LC/MS Analysis of compound **1**..........................................................S33
Figure S65. LC/MS Analysis of fraction 7.2...........................................................S34

Figure S66. Structures of TS1 and TS2....................................................................S34

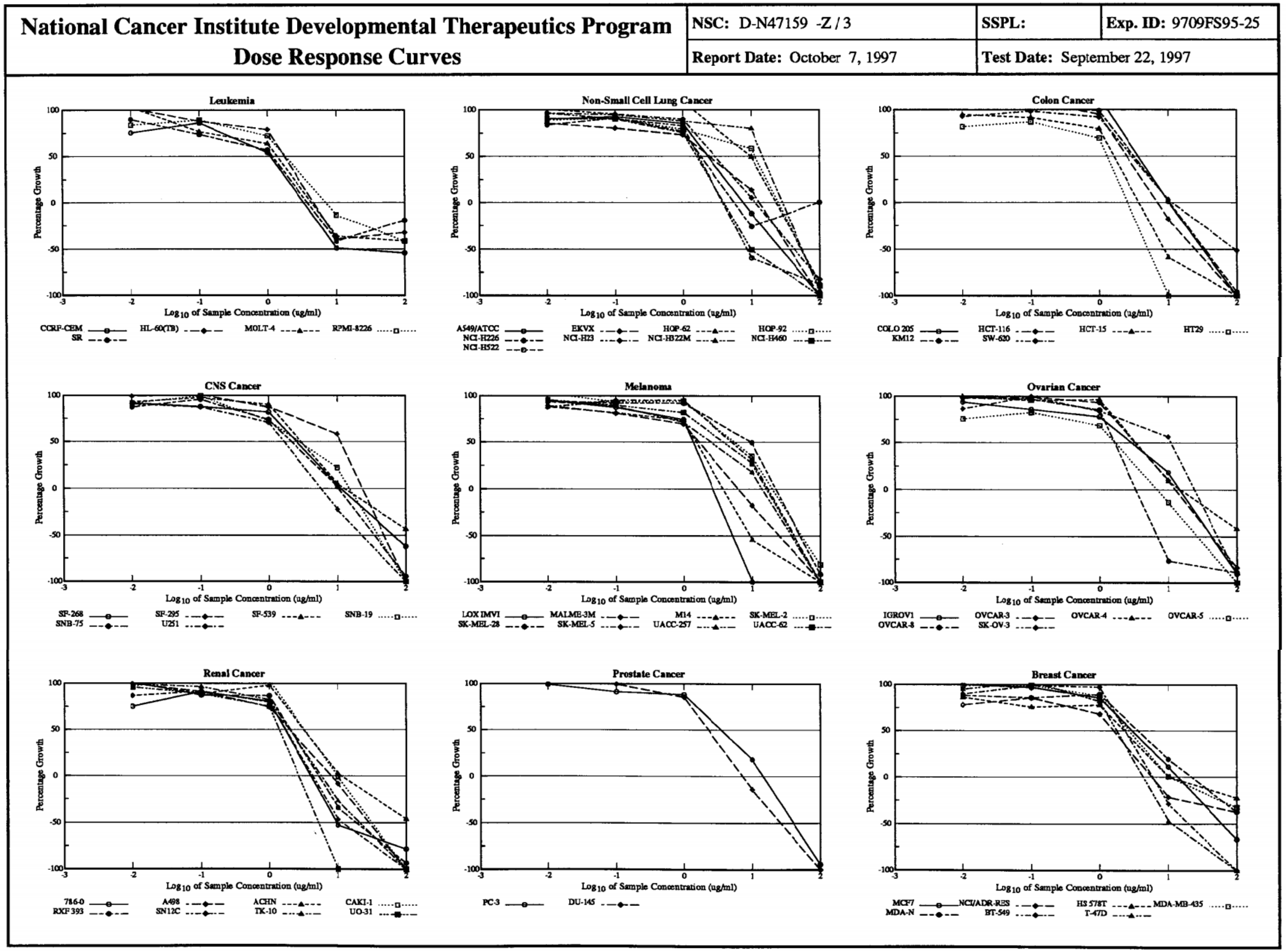


Figure S1. NCI-60 Human tumor cell line assay data for the crude organic extract of *T. rubescens* (N047159)

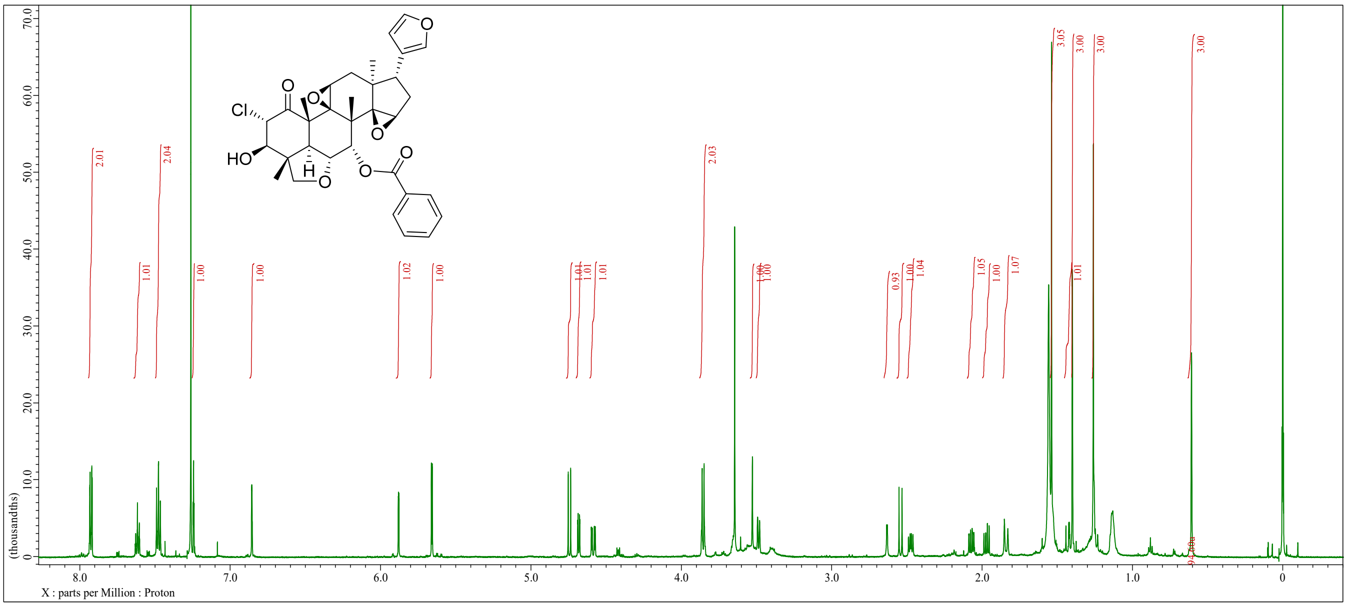

Figure S2. $^{1}$H NMR spectrum of compound **1** in $CDCl_3$ at 600MHz

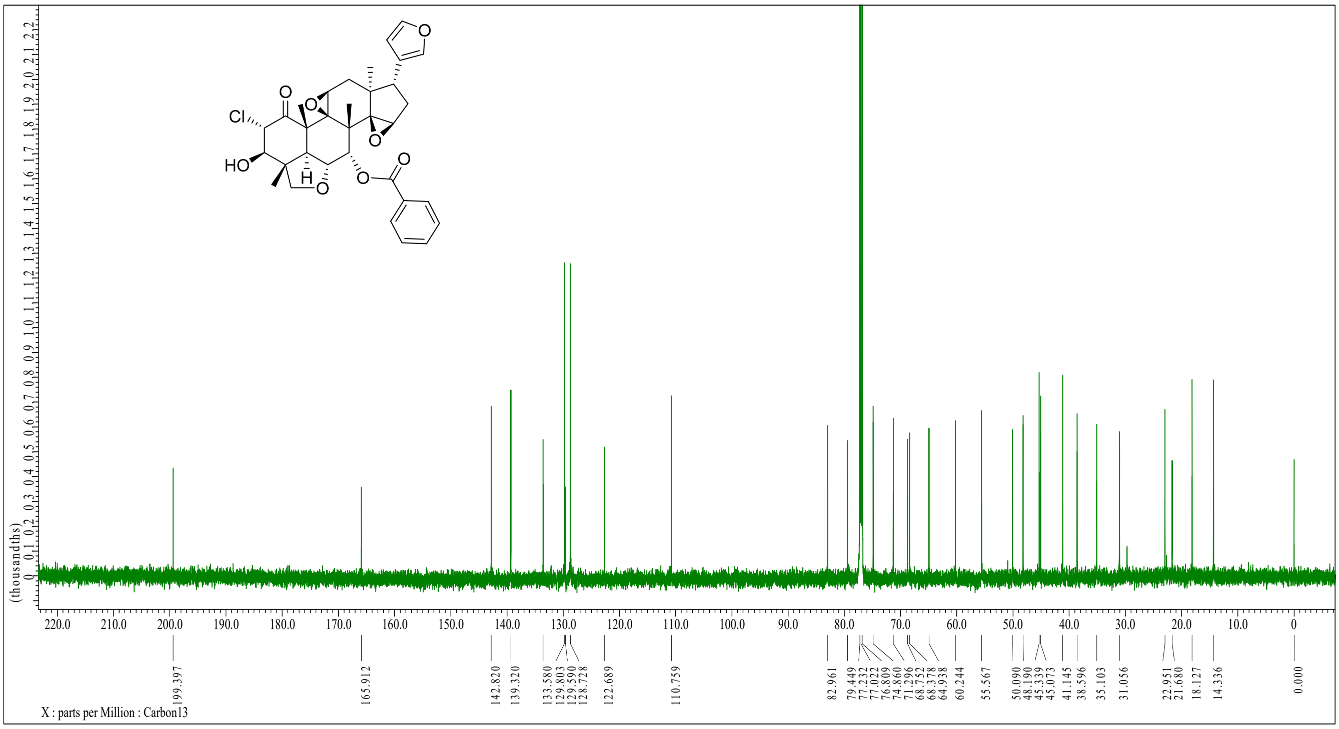

Figure S3. $^{13}$C NMR spectrum of compound **1** in $CDCl_3$ at 150MHz

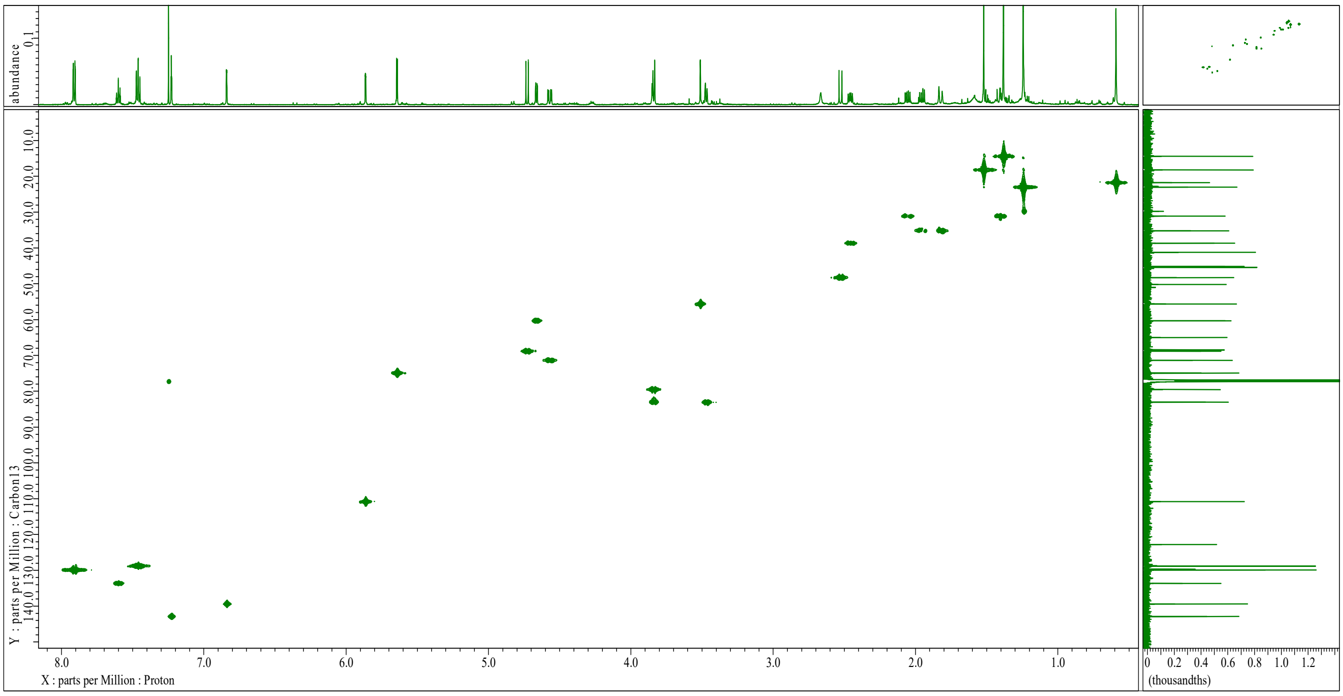

Figure S4. HMQC spectrum of compound **1** in CDCl3 at 600MHz

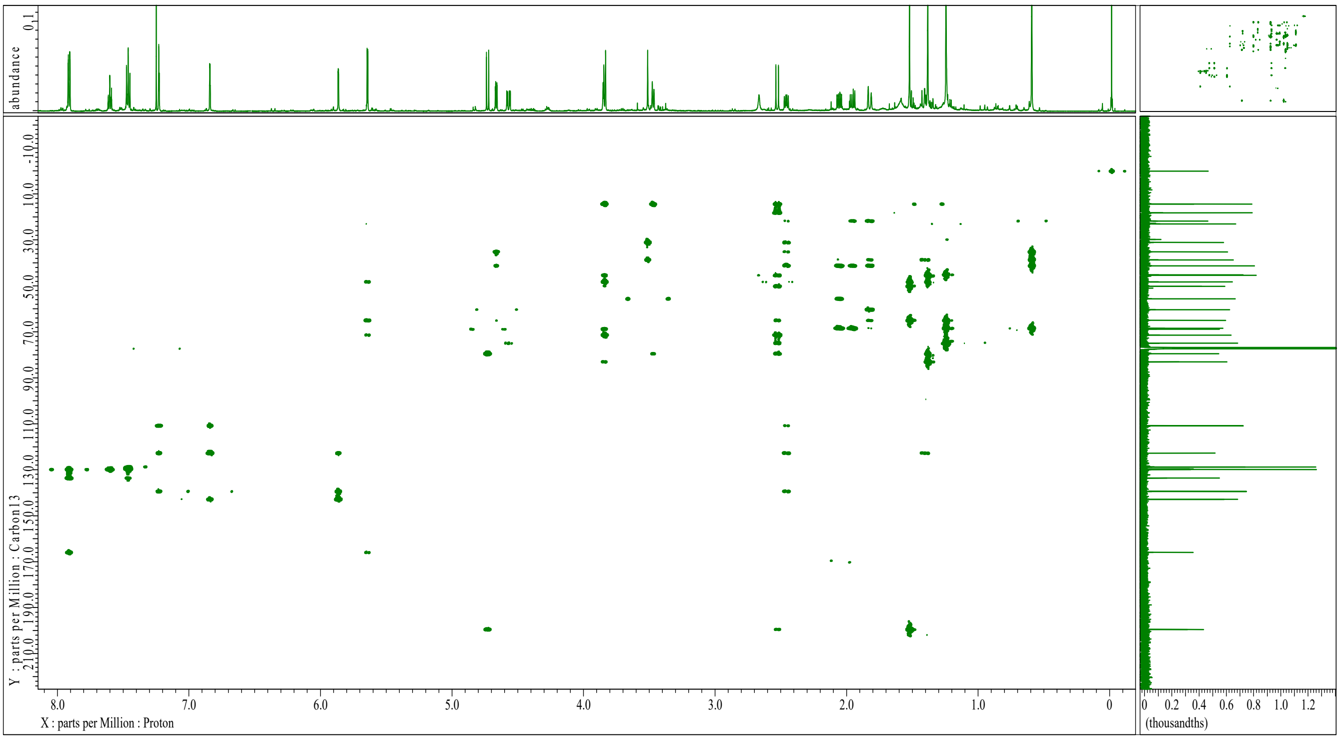

Figure S5. HMBC spectrum of compound **1** in $CDCl_3$ at 600MHz

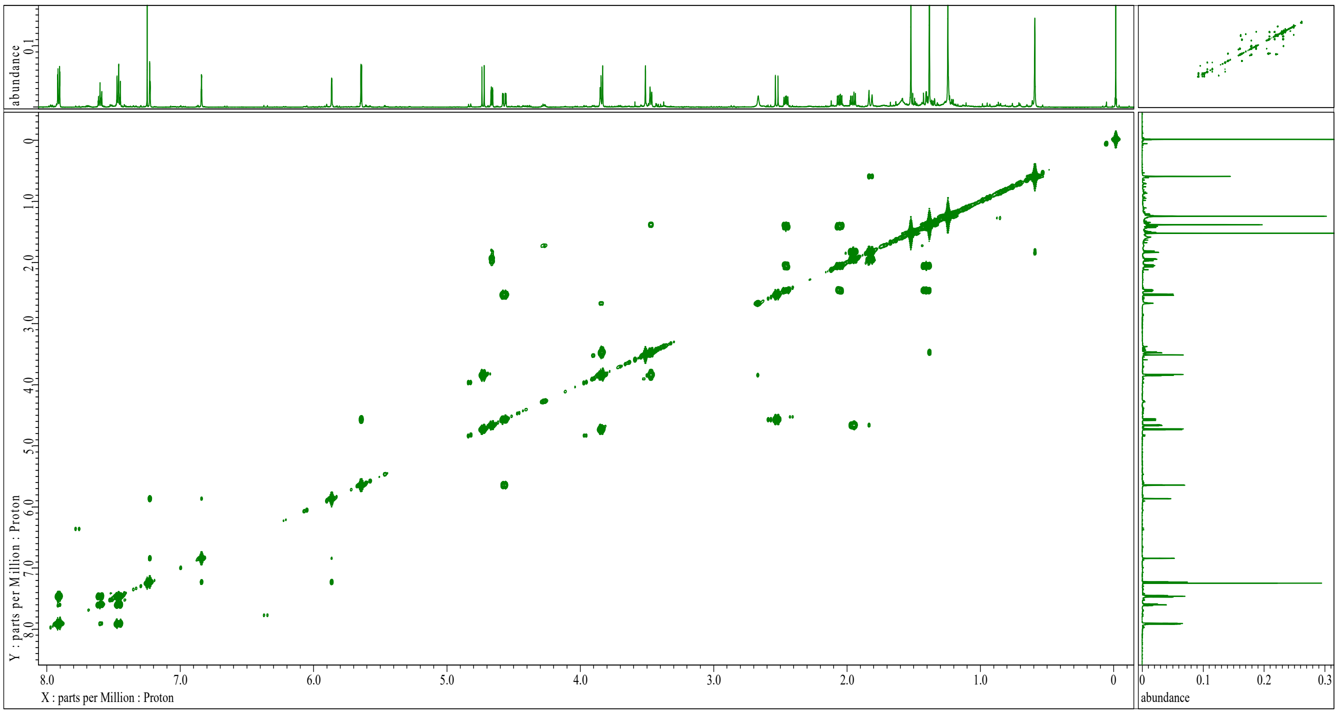


Figure S6. COSY spectrum of compound **1** in $CDCl_3$ at 600MHz

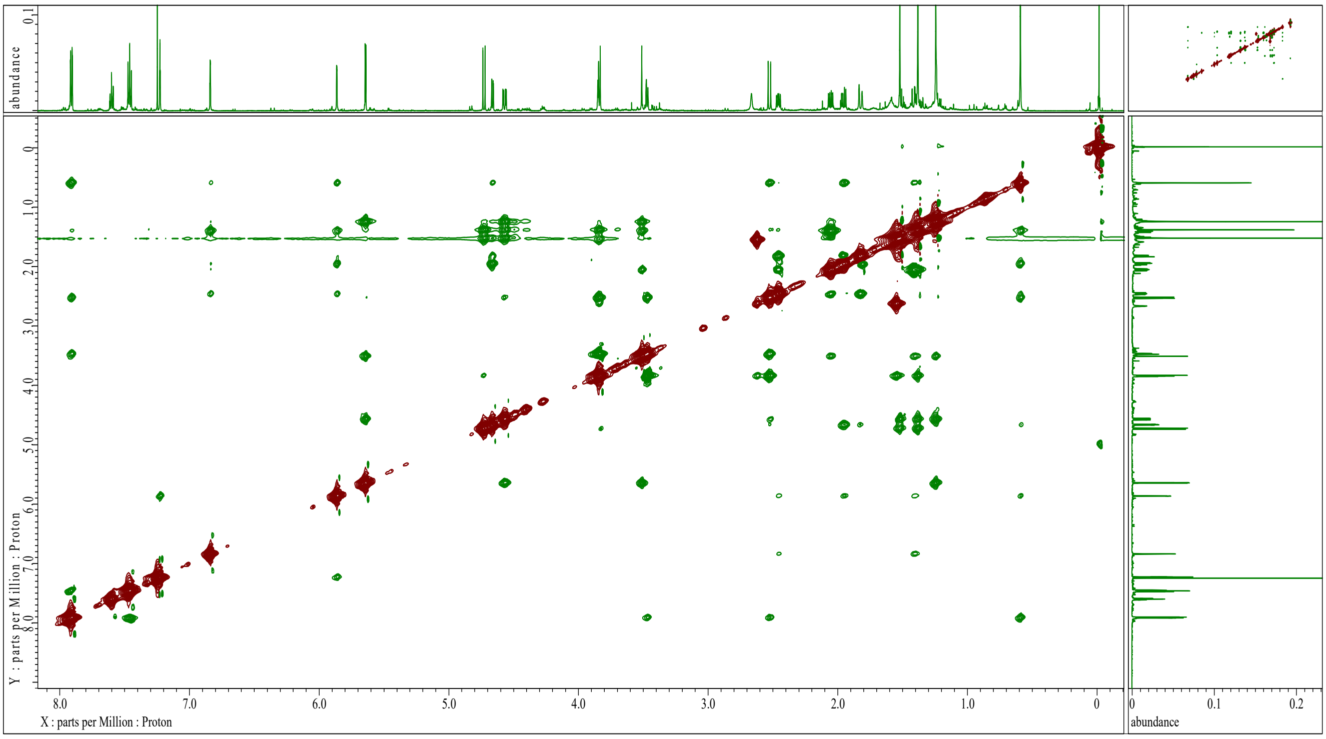


Figure S7. NOESY spectrum of compound **1** in $CDCl_3$ at 600MHz

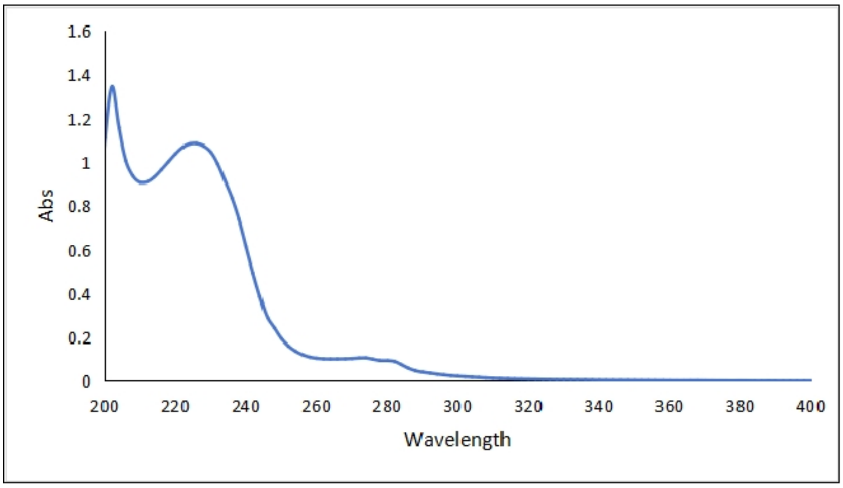


Figure S8. UV spectrum of compound **1** in MeOH

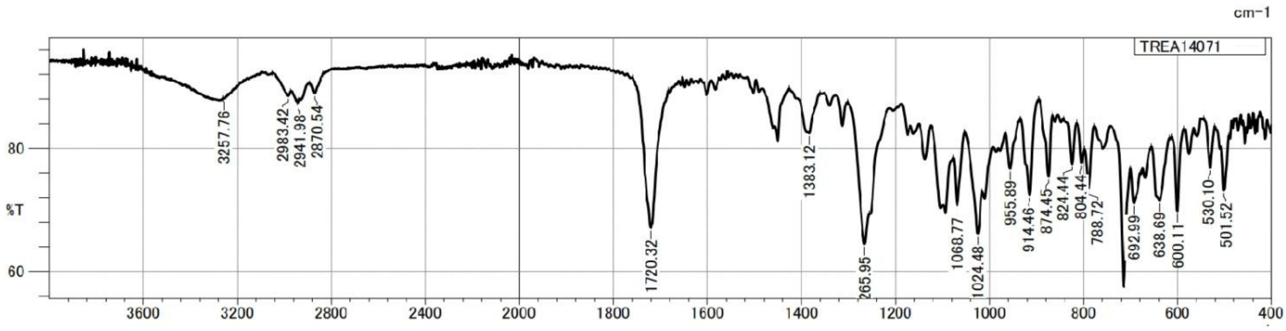


Figure S9. IR spectrum of compound **1**

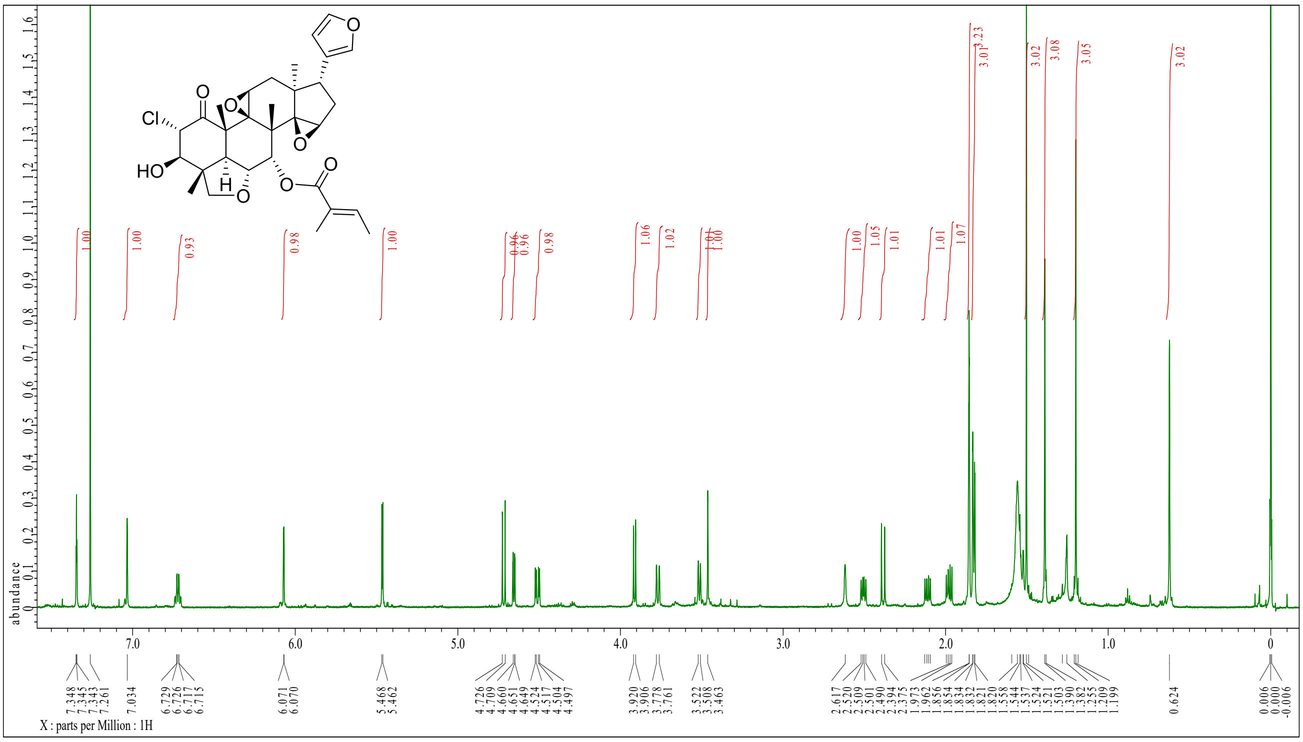


Figure S10. $^{1}$H NMR spectrum of compound **2** in $CDCl_3$ at 600MHz

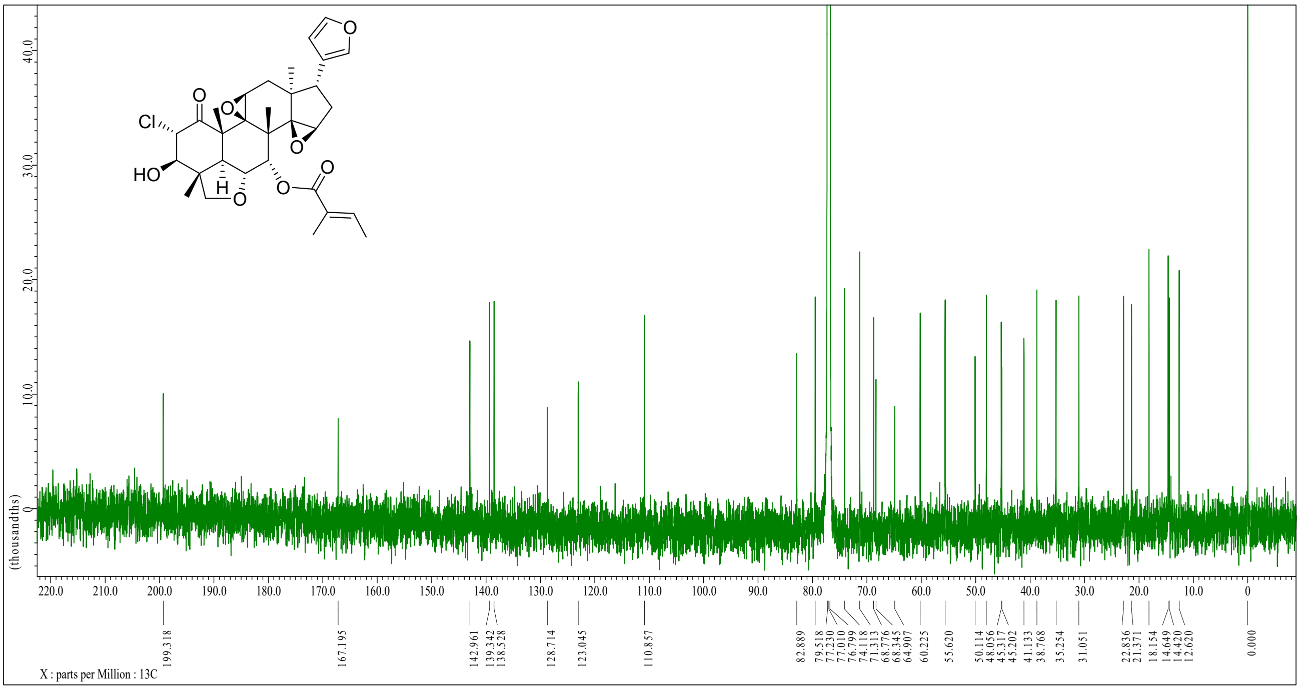


Figure S11.$^{13}$C NMR spectrum of compound **2** in $CDCl_3$ at 150MHz

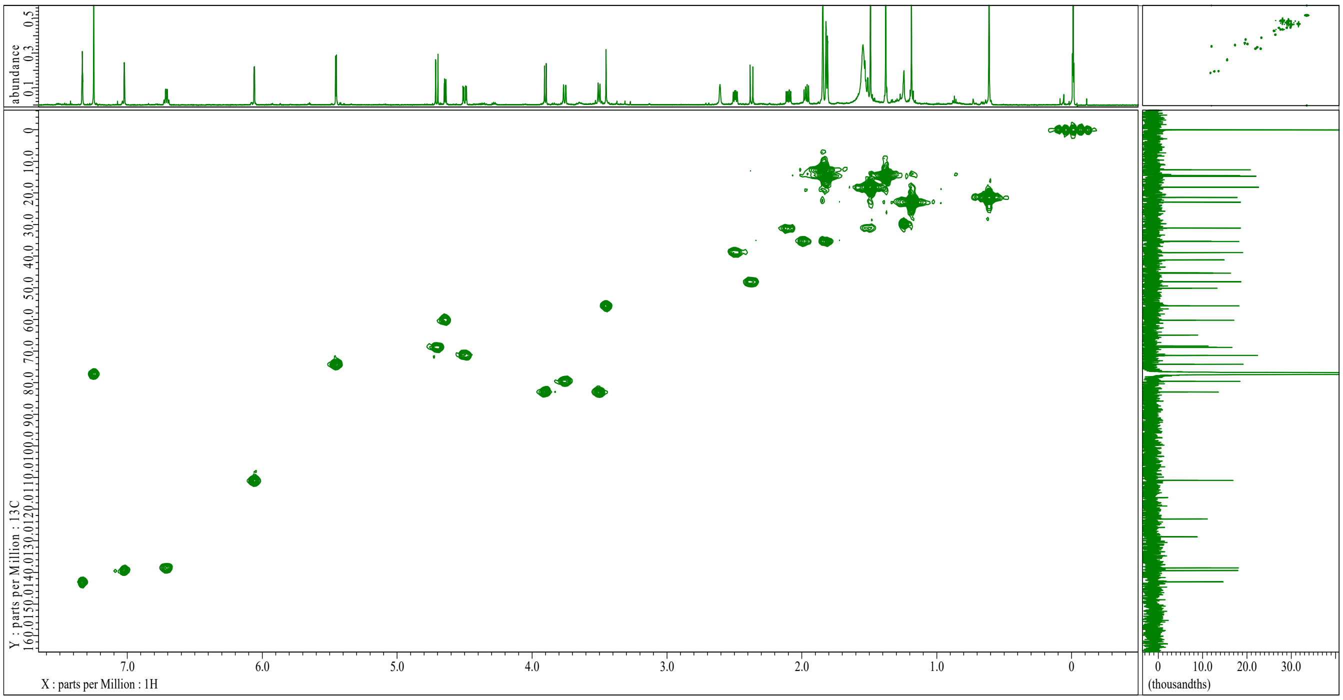


Figure S12. HMQC spectrum of compound **2** in $CDCl_3$ at 600MHz

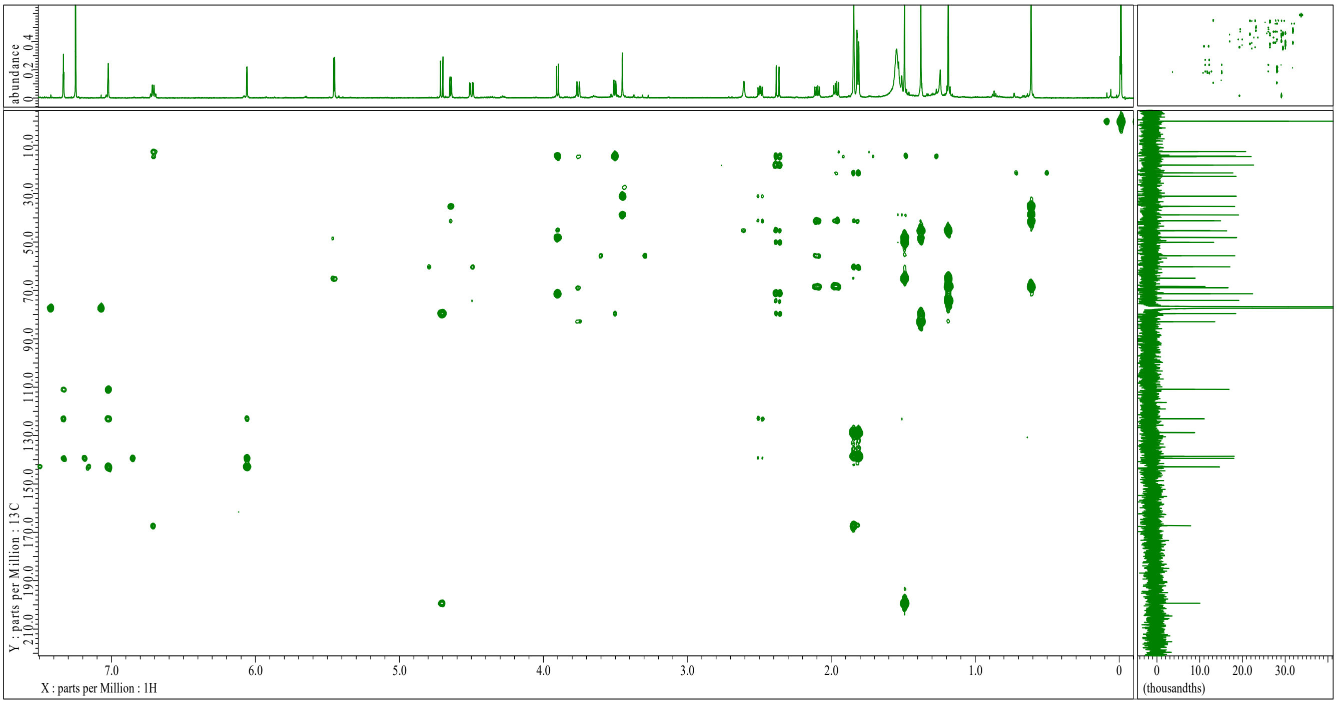


Figure S13. HMBC spectrum of compound **2** in $CDCl_3$ at 600MHz

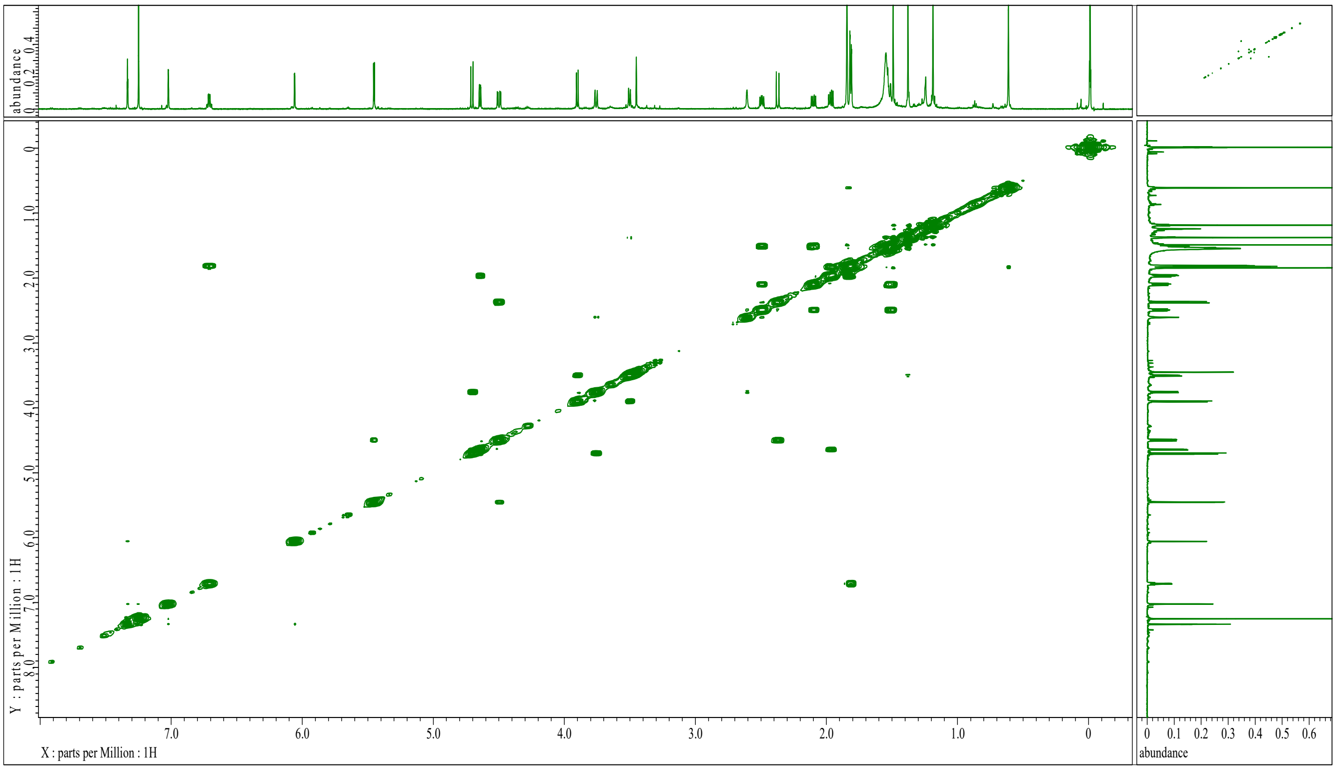


Figure S14. COSY spectrum of compound **2** in $CDCl_3$ at 600MHz

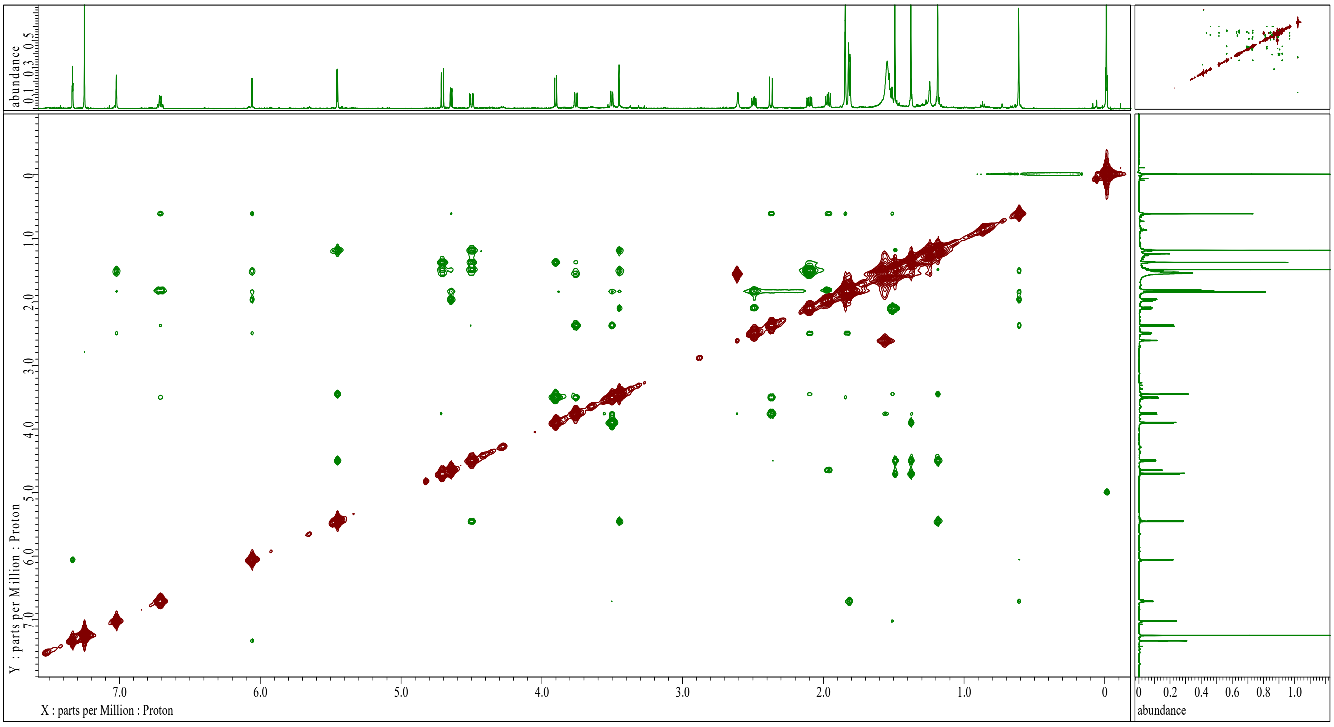


Figure S15. NOESY spectrum of compound **2** in $CDCl_3$ at 600MHz

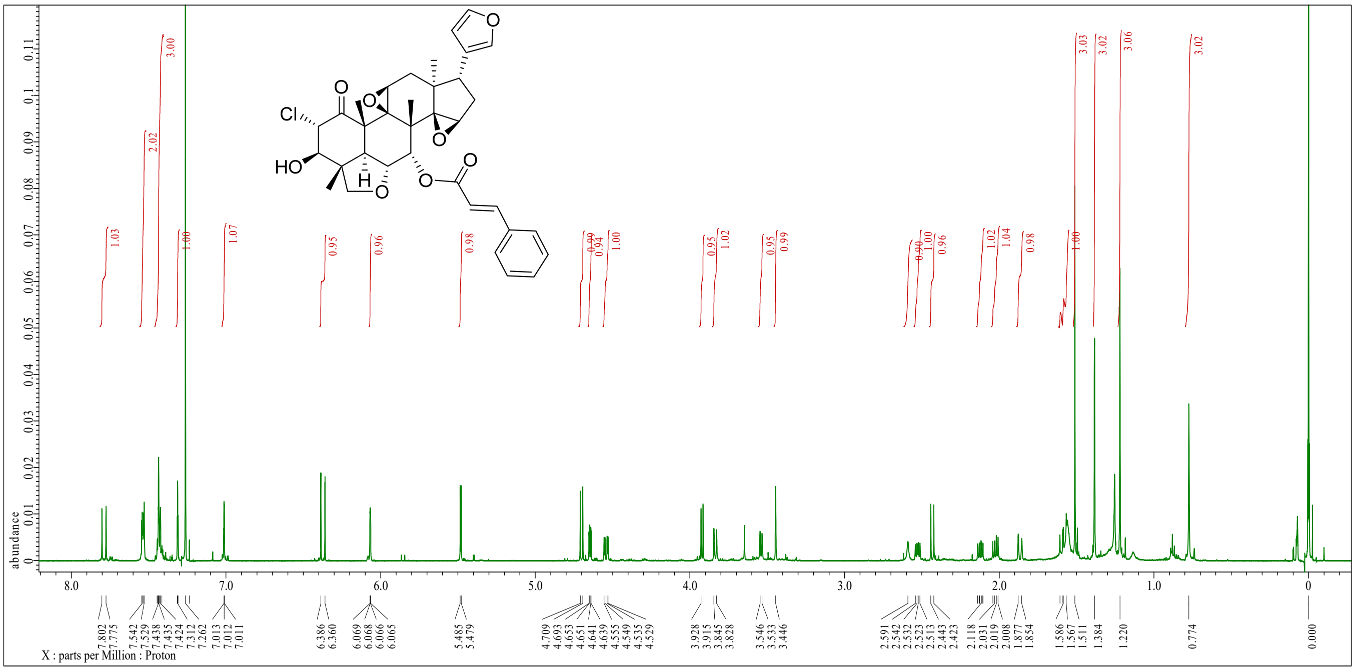

Figure S16. $^{1}$H NMR spectrum of compound **3** in $CDCl_3$ at 600MHz

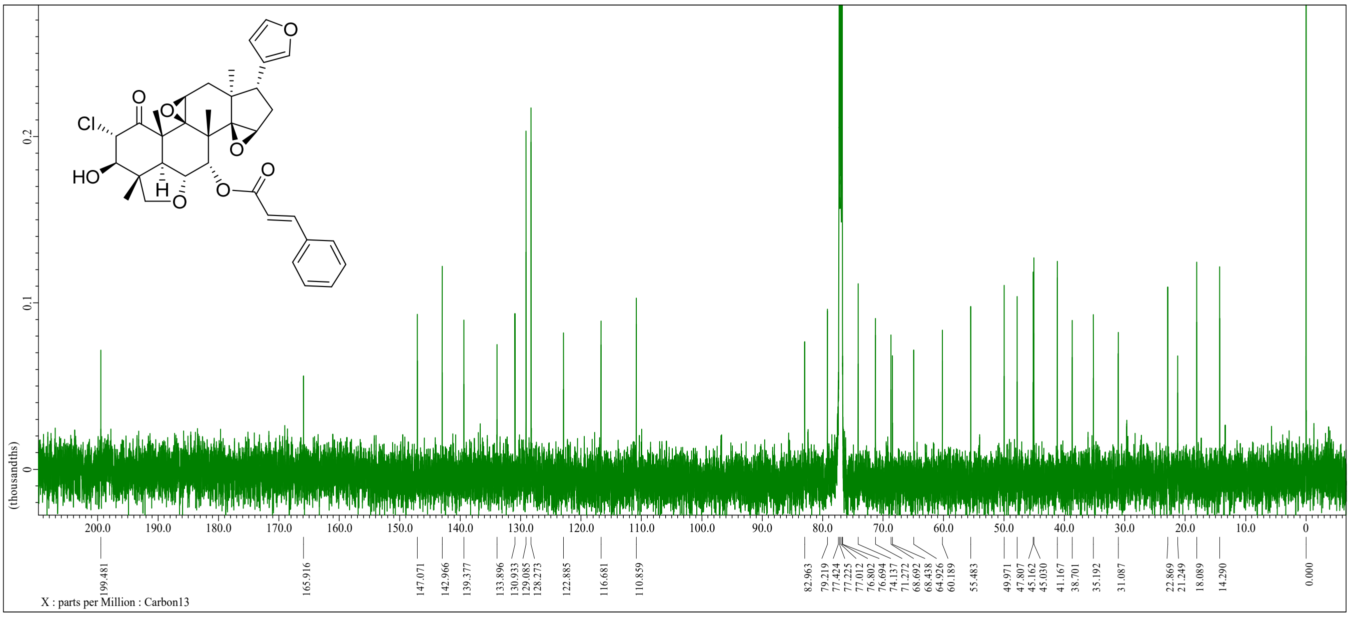

Figure S17.$^{13}$C NMR spectrum of compound **3** in $CDCl_3$ at 150MHz

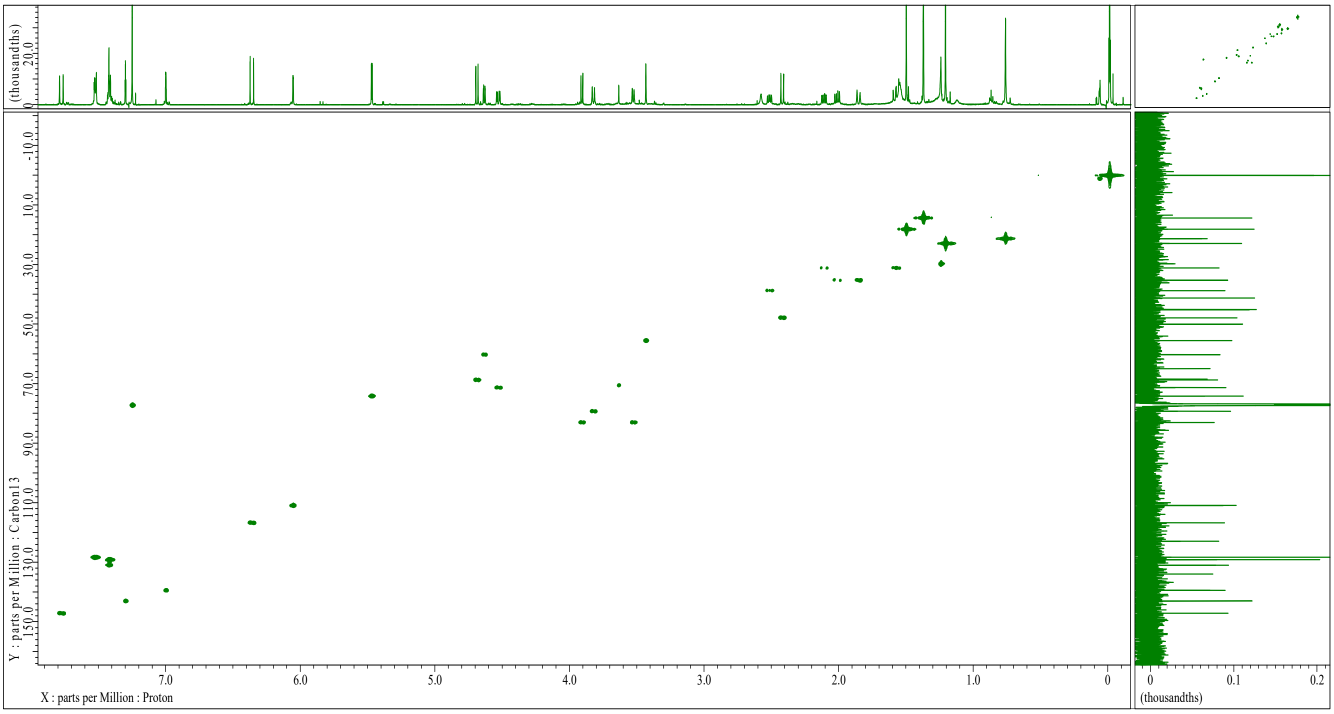


Figure S18. HMQC spectrum of compound **3** in $CDCl_3$ at 600MHz

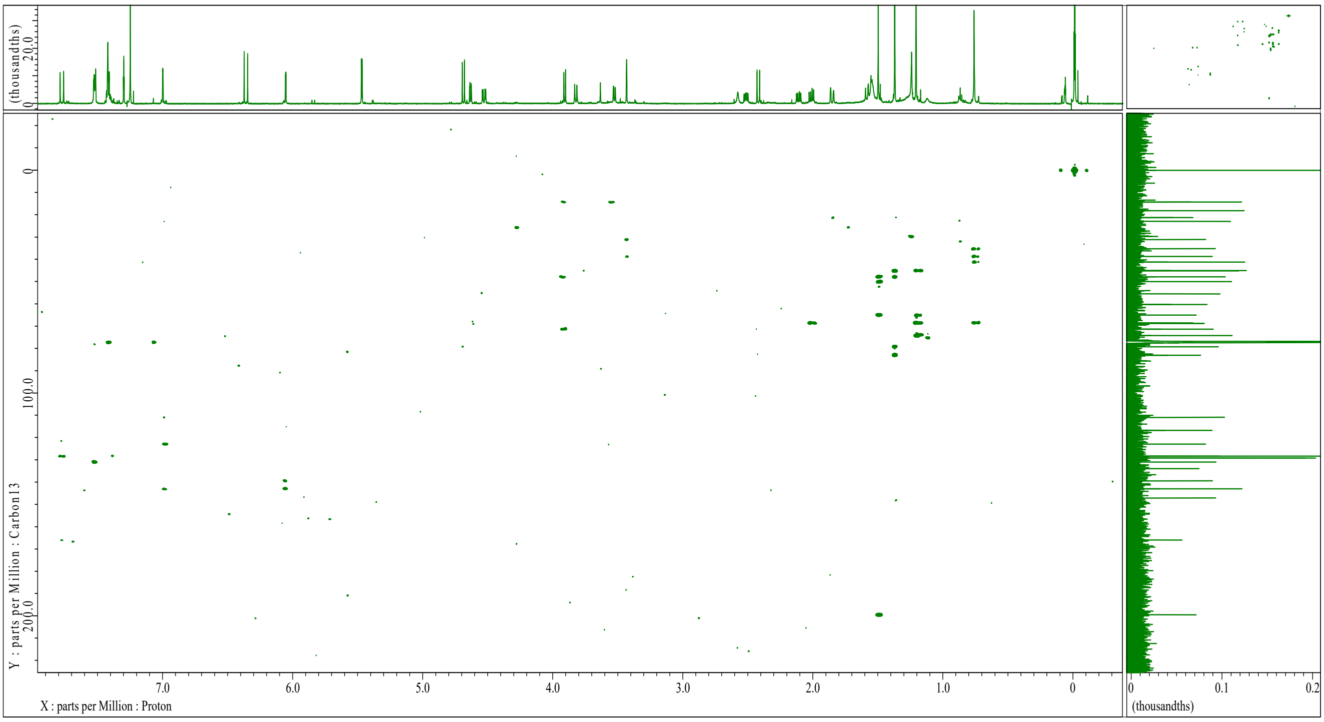


Figure S19. HMBC spectrum of compound **3** in $CDCl_3$ at 600MHz

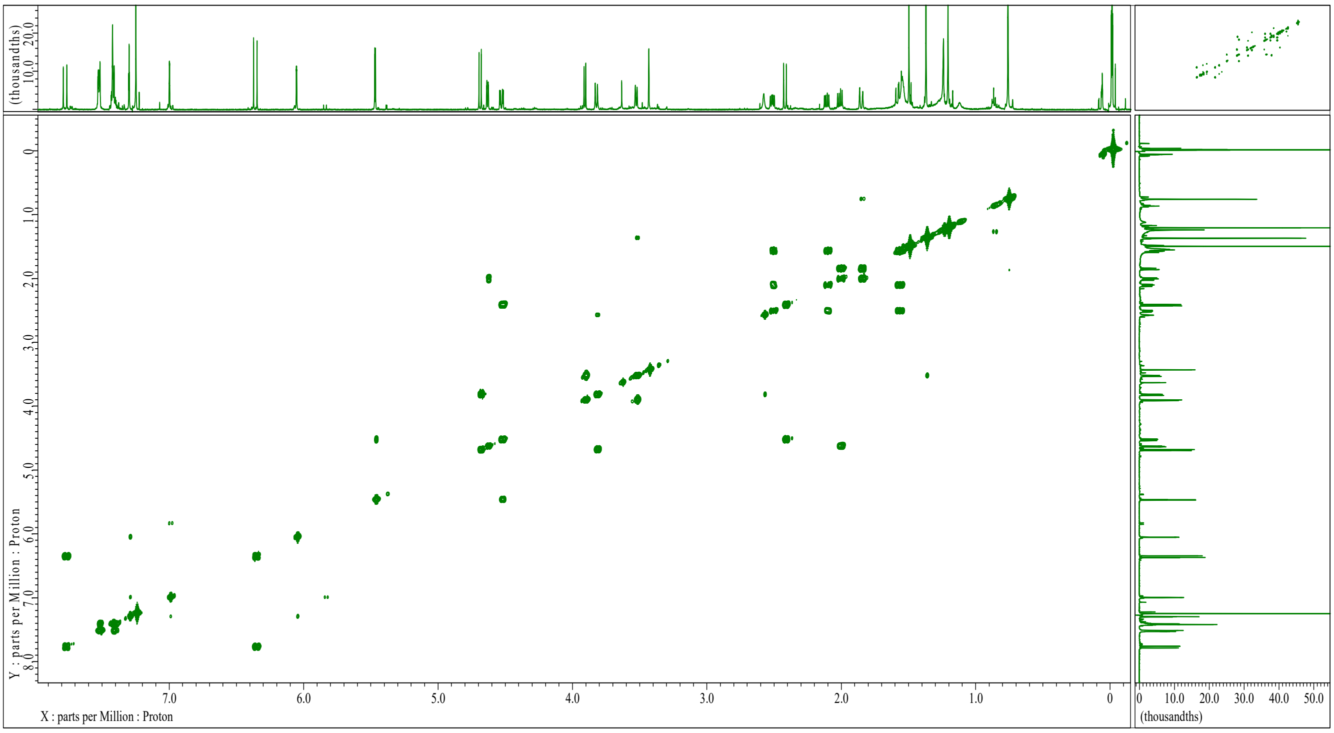


Figure S20. COSY spectrum of compound **3** in $CDCl_3$ at 600MHz

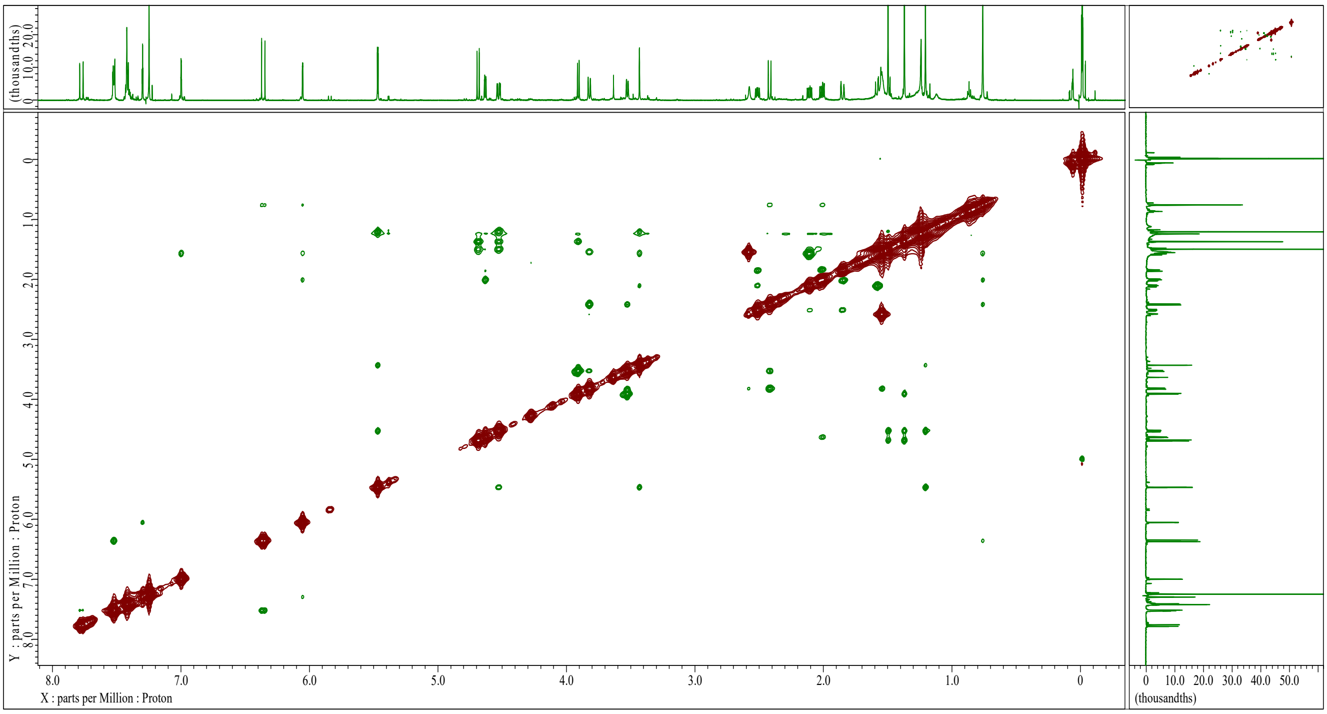


Figure S21. NOESY spectrum of compound **3** in $CDCl_3$ at 600MHz

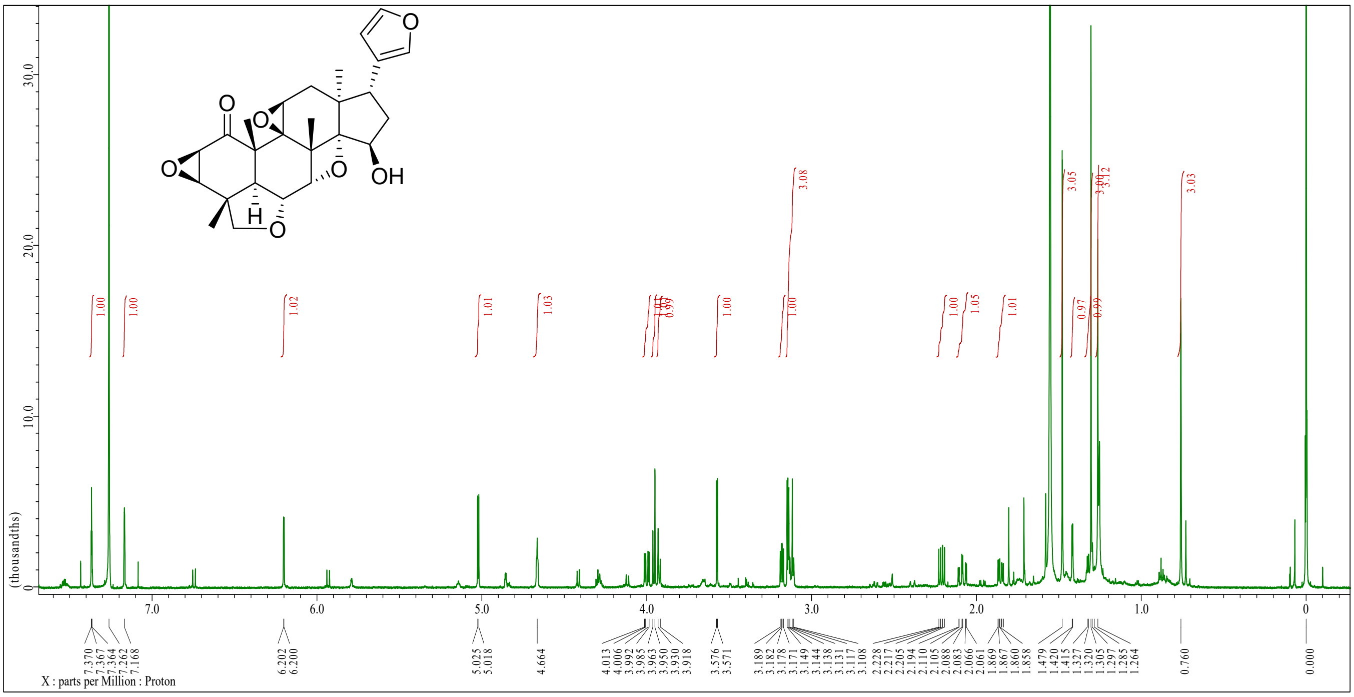

Figure S22. $^{1}$H NMR spectrum of compound **4** in $CDCl_3$ at 600MHz

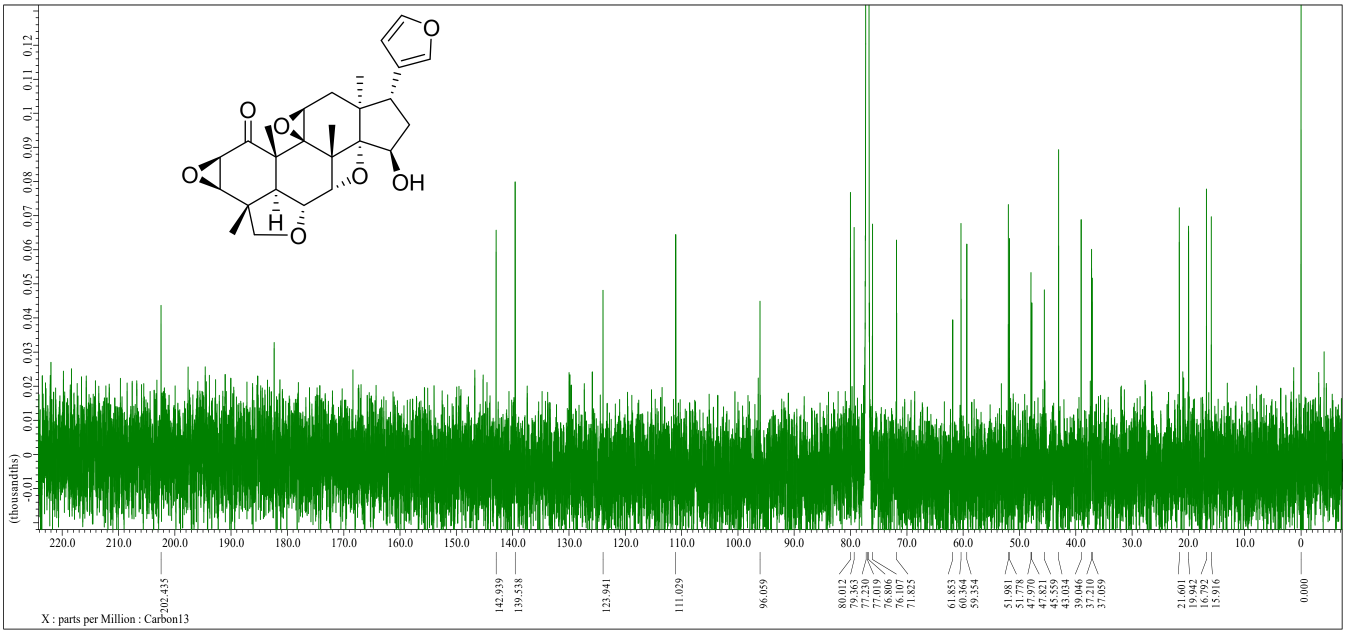

Figure S23. $^{13}$C NMR spectrum of compound **4** in $CDCl_3$ at 150MHz

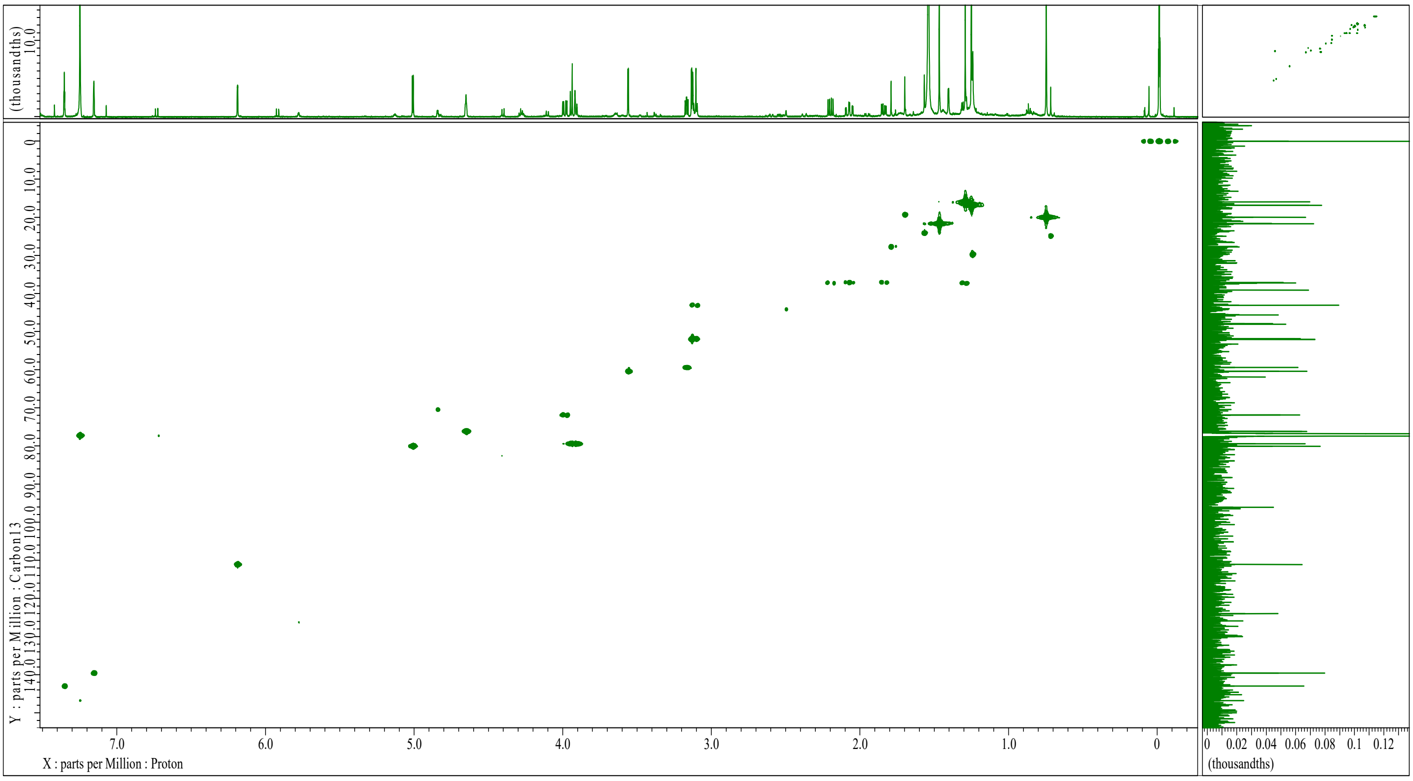


Figure S24. HMQC spectrum of compound **4** in $CDCl_3$ at 600MHz

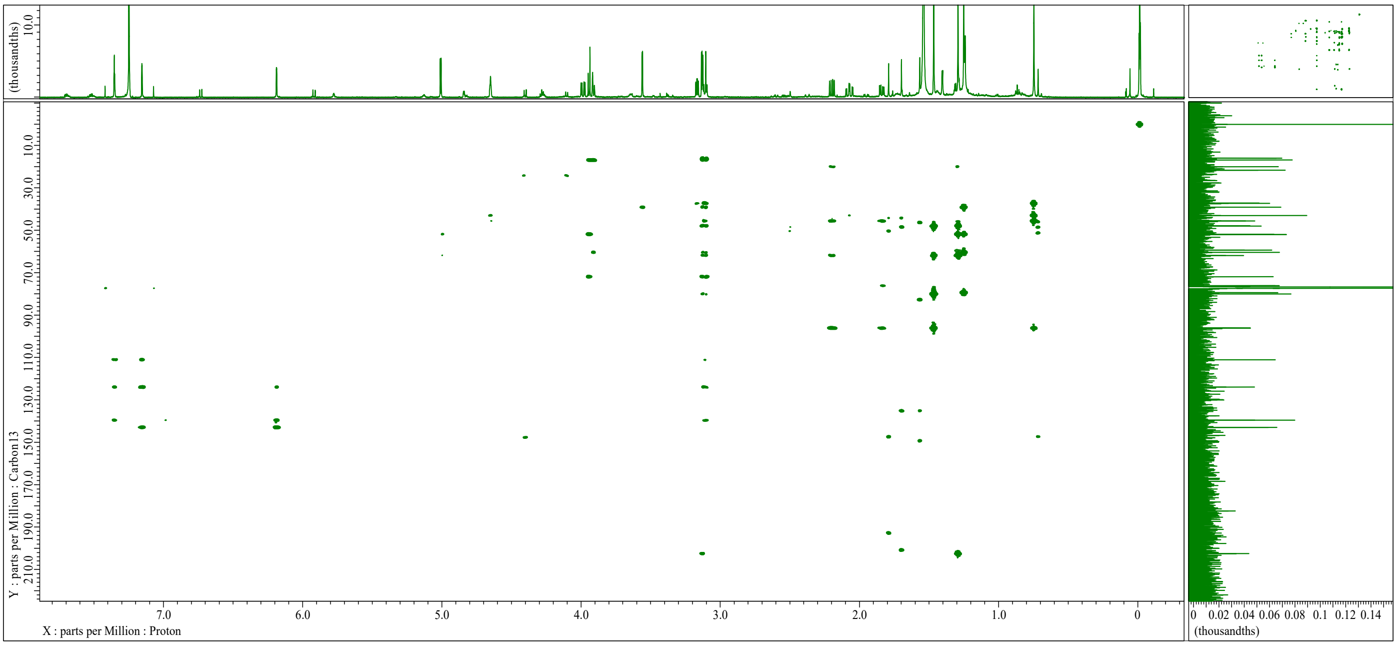


Figure S25. HMBC spectrum of compound **4** in $CDCl_3$ at 600MHz

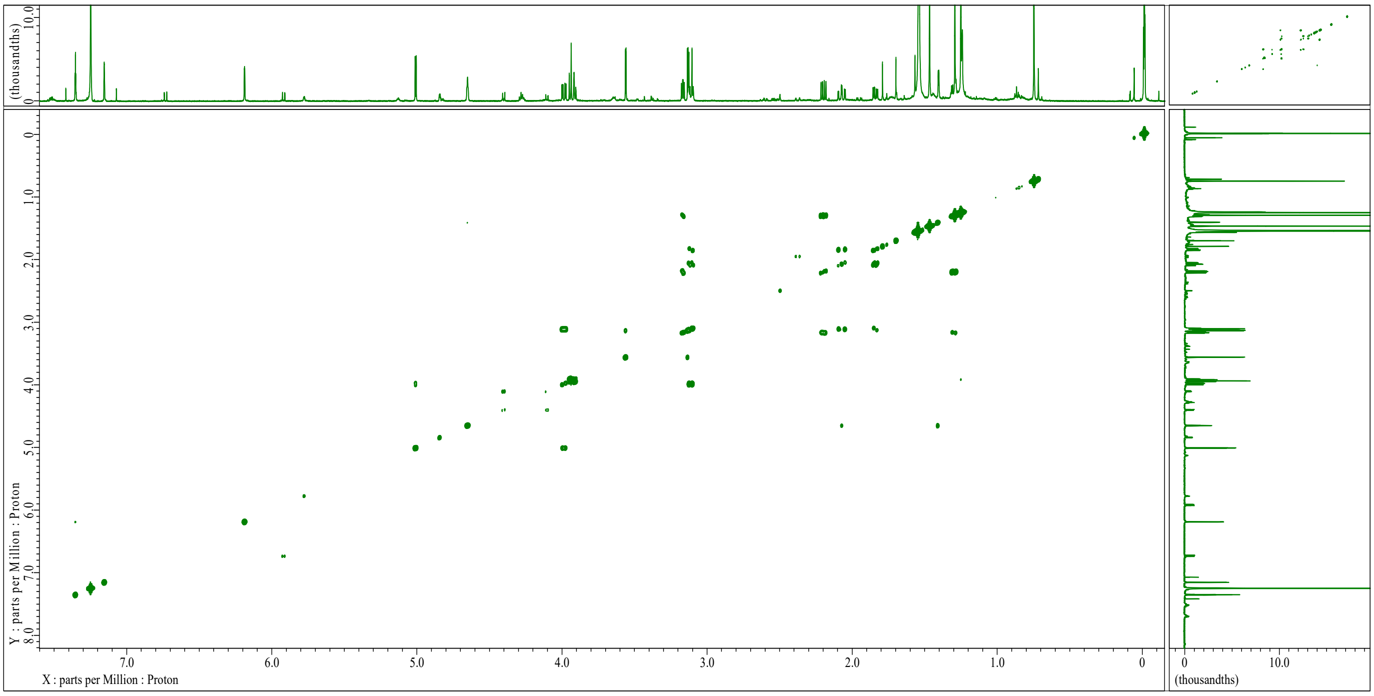


Figure S26. COSY spectrum of compound **4** in $CDCl_3$ at 600MHz

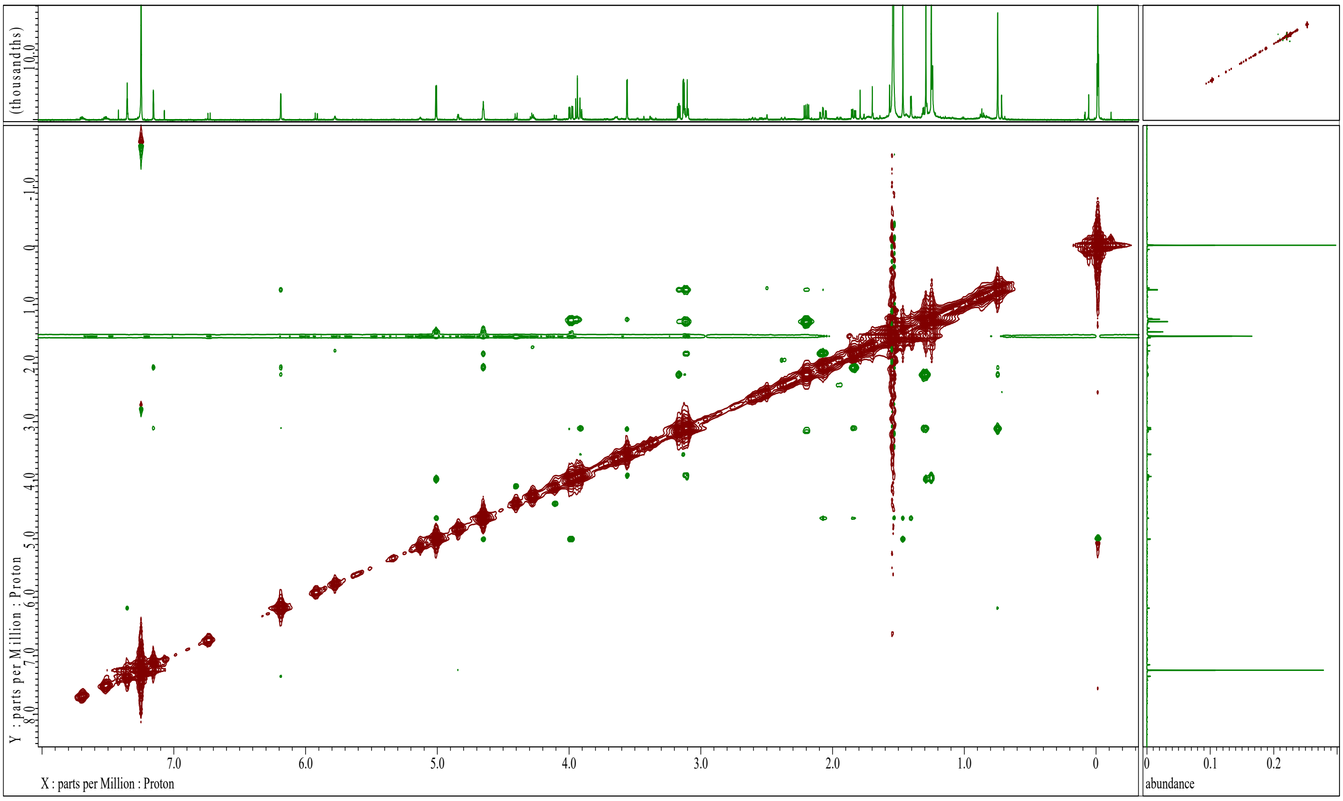


Figure S27. NOESY spectrum of compound **4** in $CDCl_3$ at 600MHz

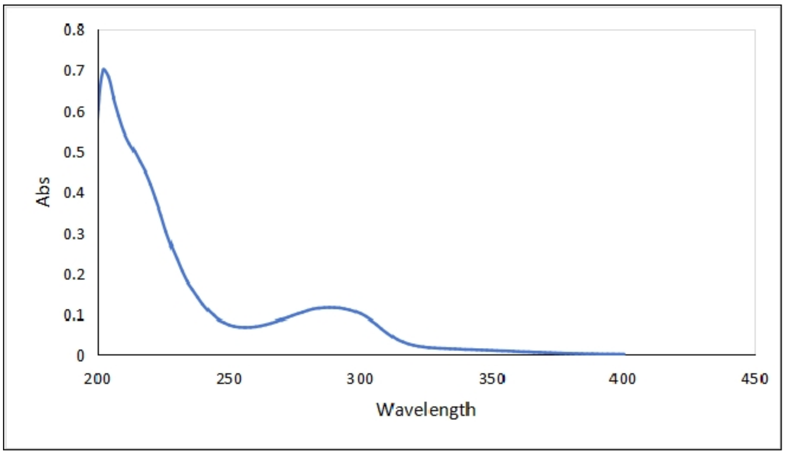


Figure S28. UV spectrum of compound **4** in MeOH

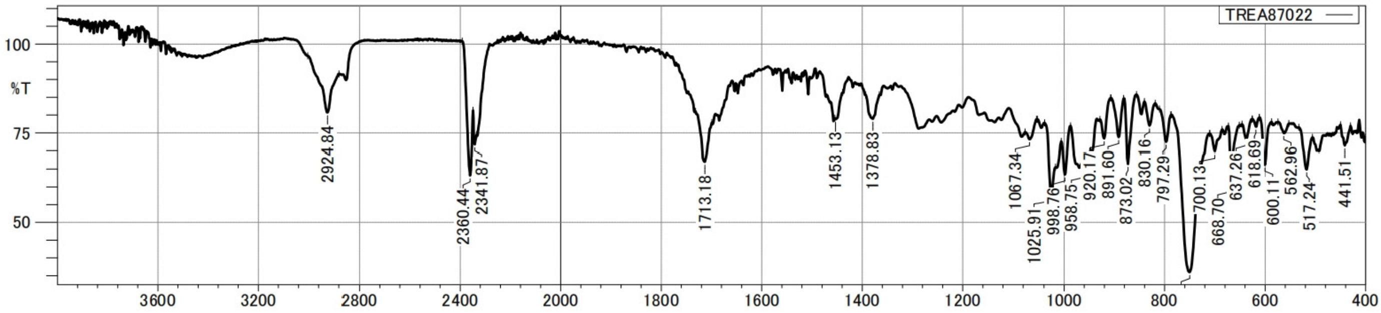


Figure S29. IR spectrum of compound **4**

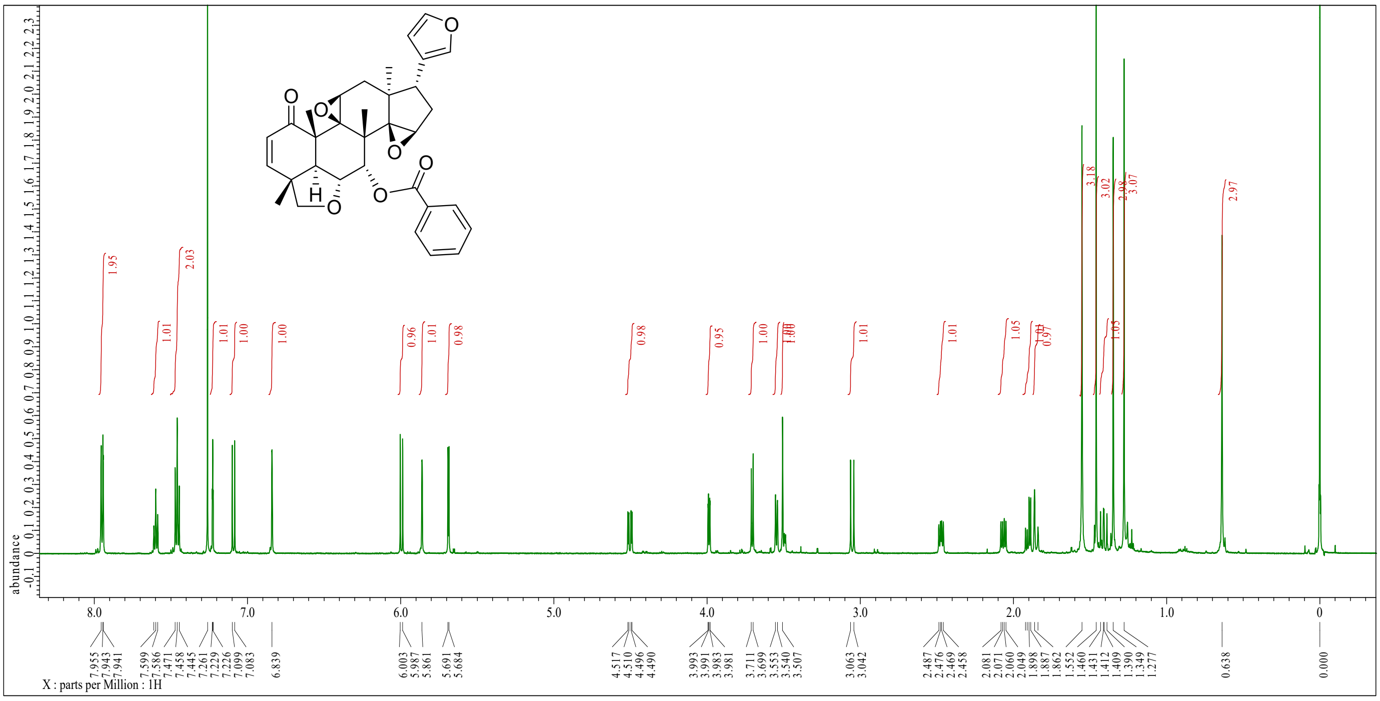

Figure S30. $^{1}$H NMR spectrum of compound **5** in $CDCl_3$ at 600MHz

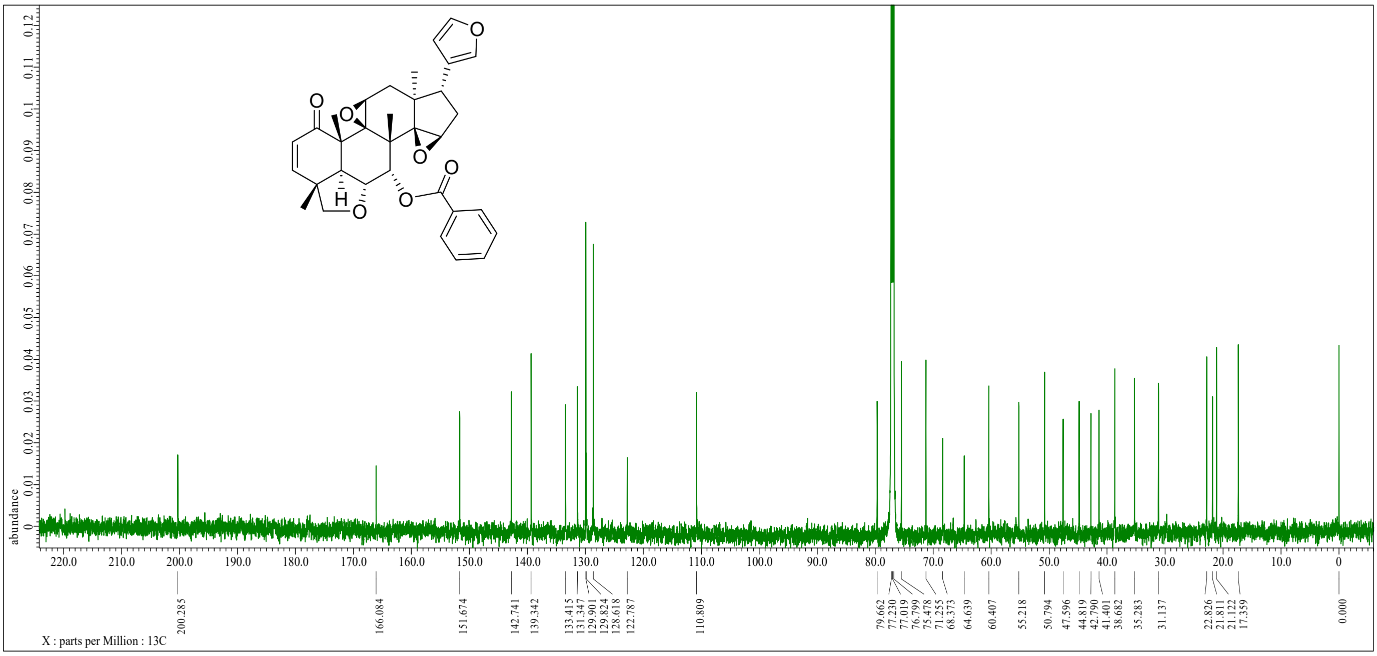

Figure S31. $^{13}$C NMR spectrum of compound **5** in $CDCl_3$ at 150MHz

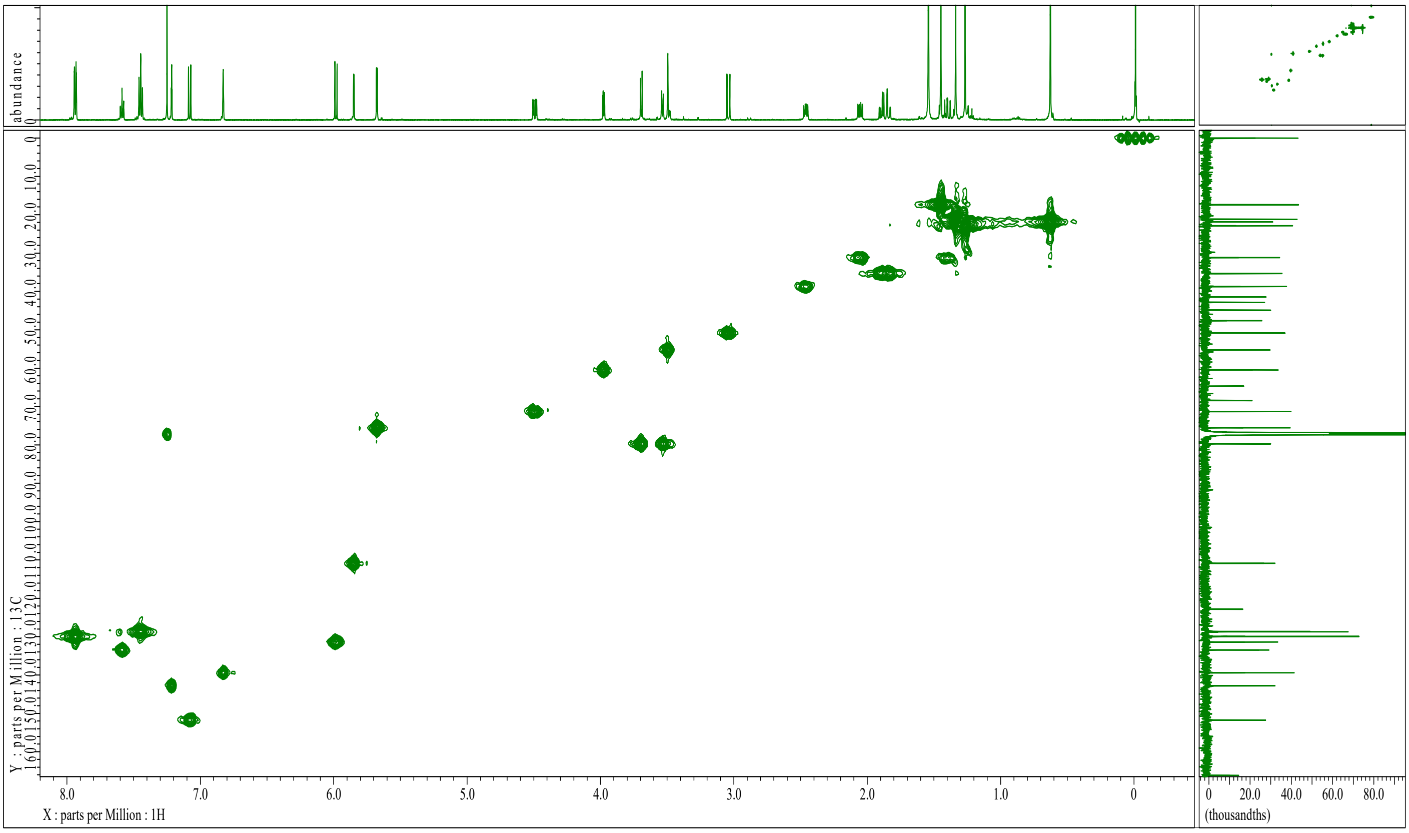


Figure S32. HMQC spectrum of compound **5** in $CDCl_3$ at 600MHz

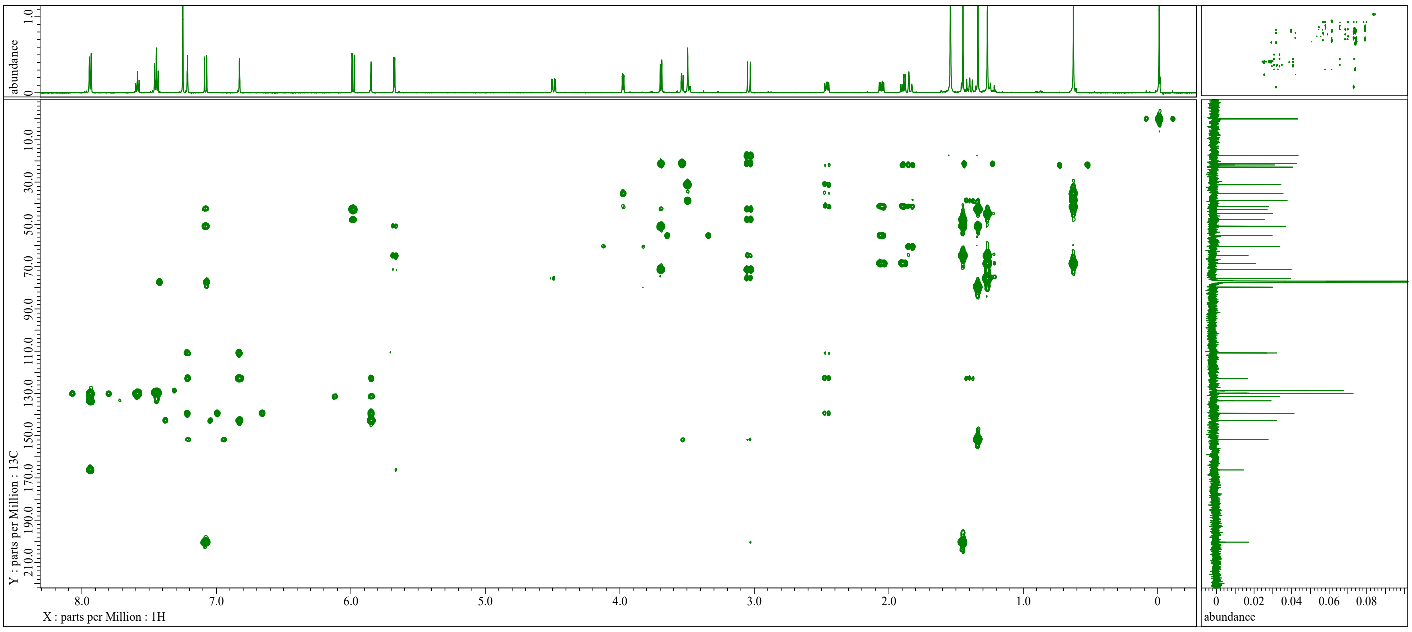


Figure S33. HMBC spectrum of compound **5** in $CDCl_3$ at 600MHz

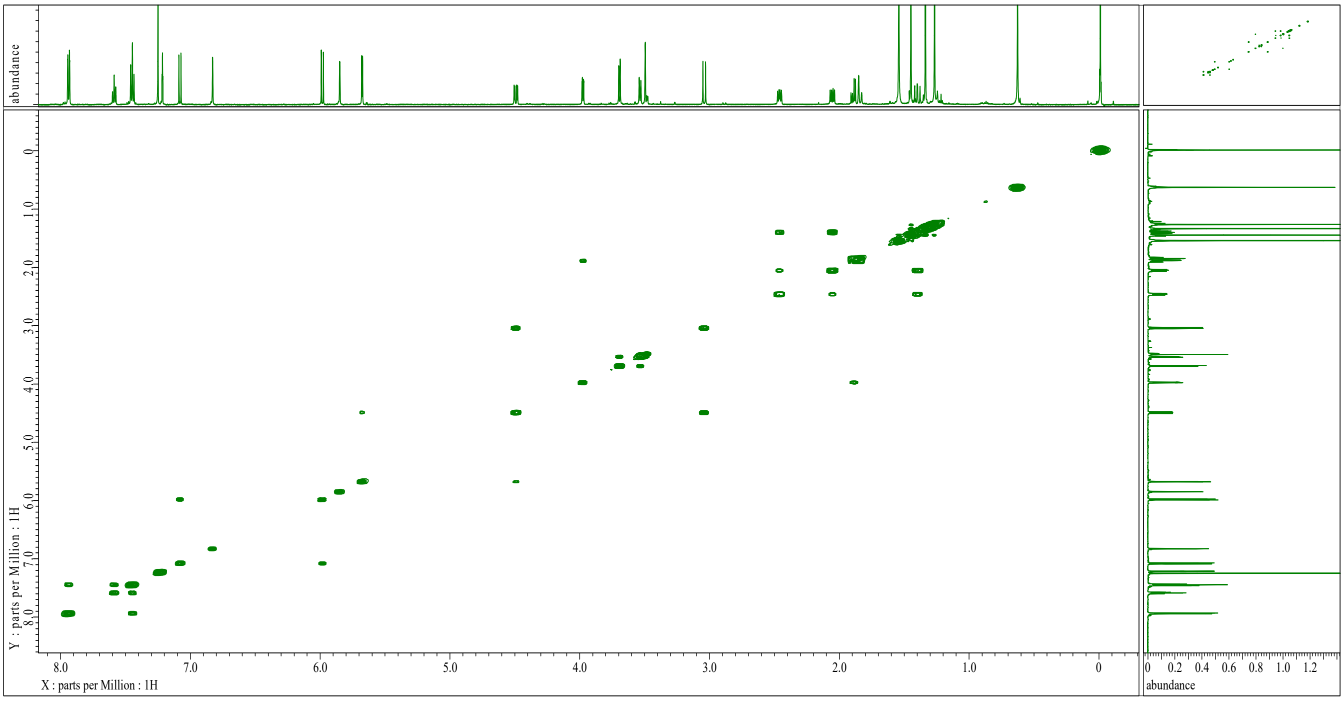


Figure S34. COSY spectrum of compound **5** in $CDCl_3$ at 600MHz

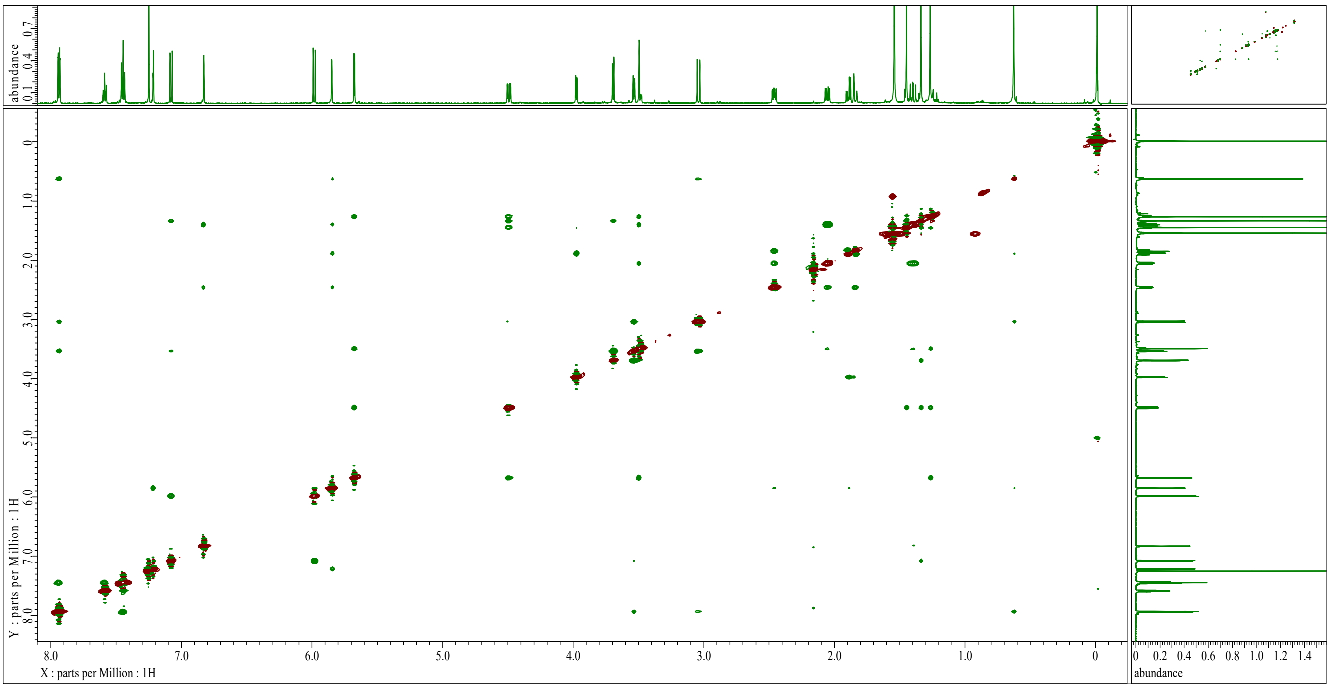


Figure S35. NOESY spectrum of compound **5** in $CDCl_3$ at 600MHz

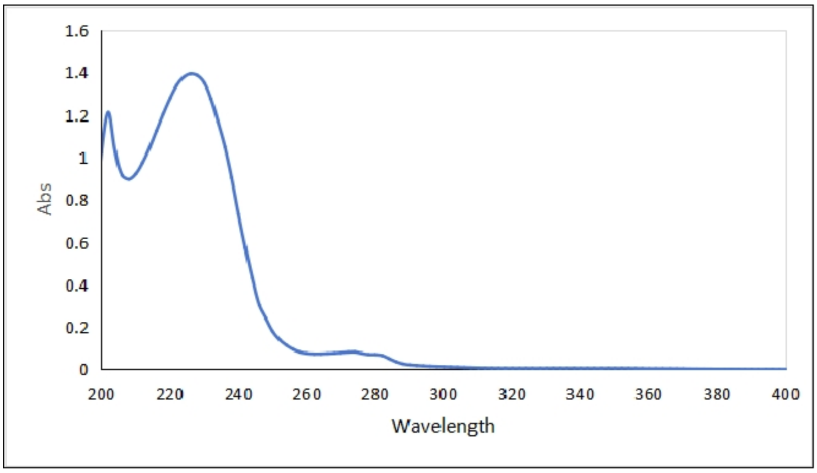


Figure S36. UV spectrum of compound **5** in MeOH

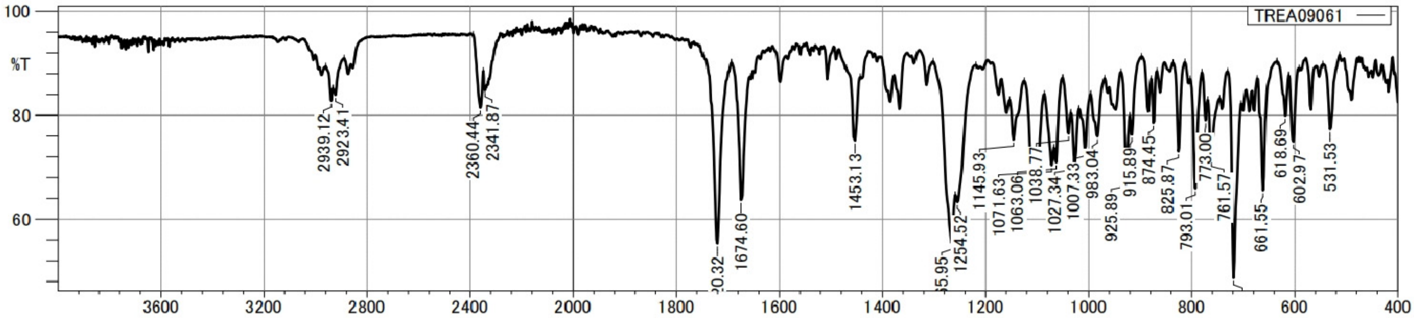


Figure S37. IR spectrum of compound **5**

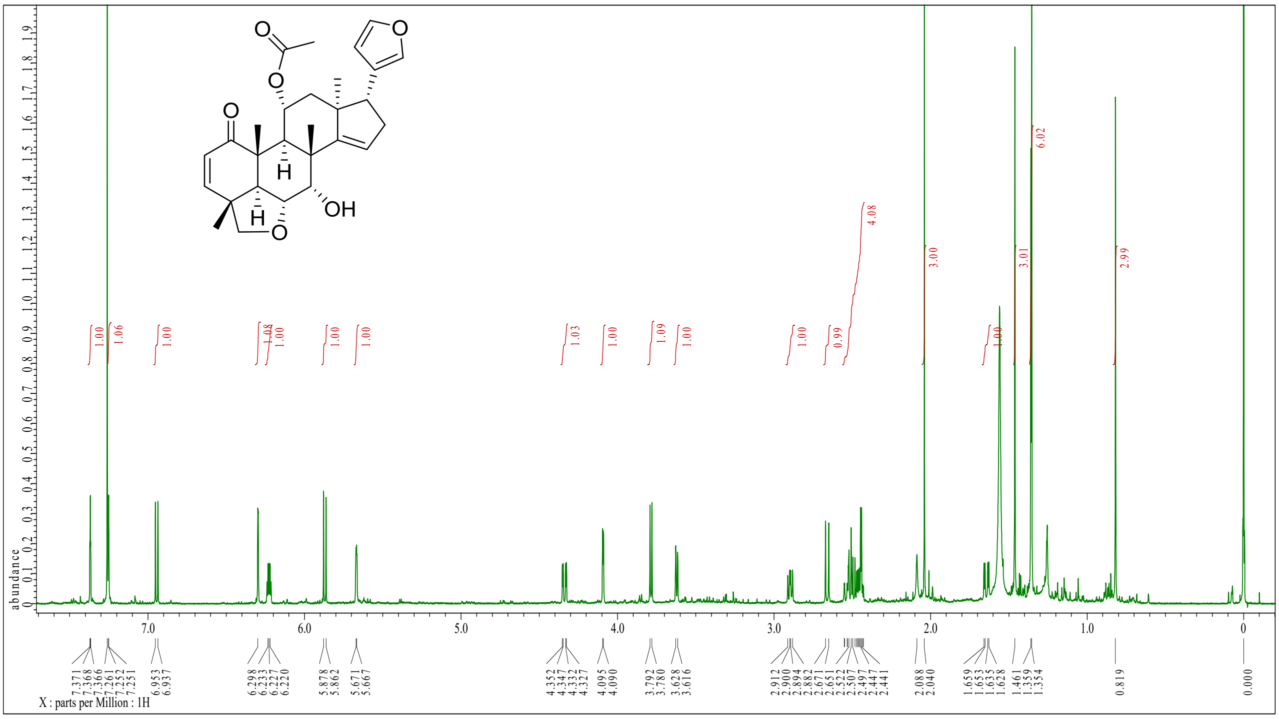

Figure S38. $^{1}H$ NMR spectrum of compound **6** in $CDCl_3$ at 600MHz

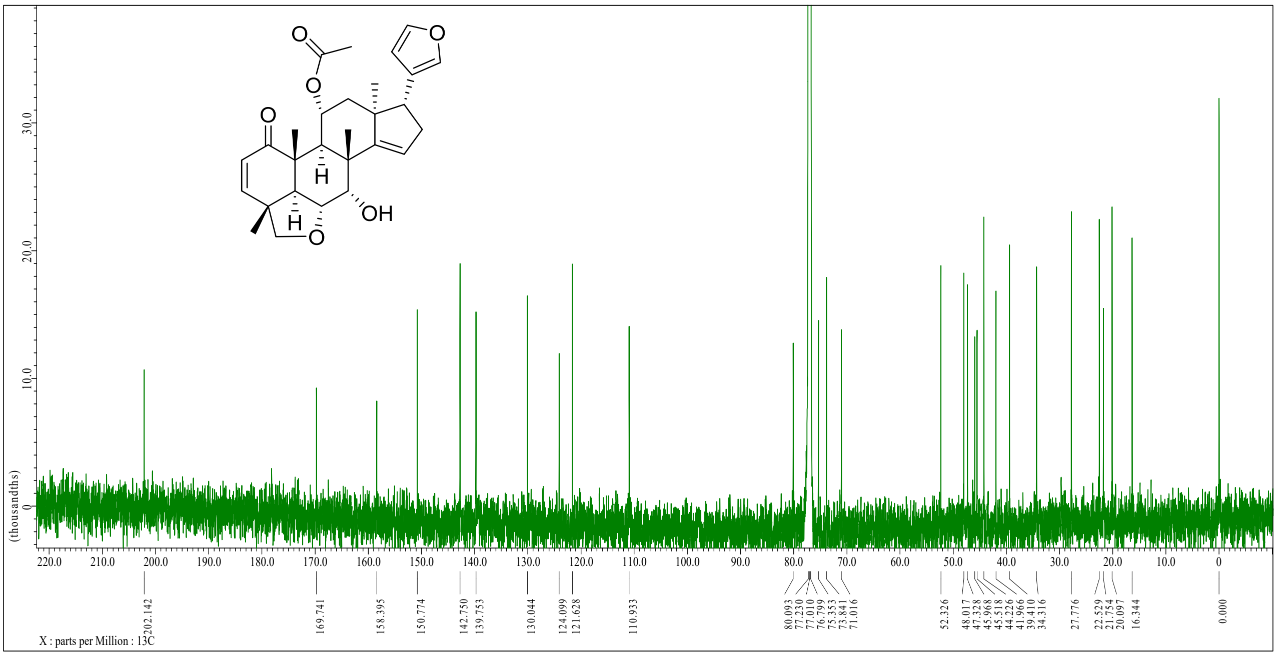

Figure S39. $^{13}C$ NMR spectrum of compound **6** in $CDCl_3$ at 150MHz

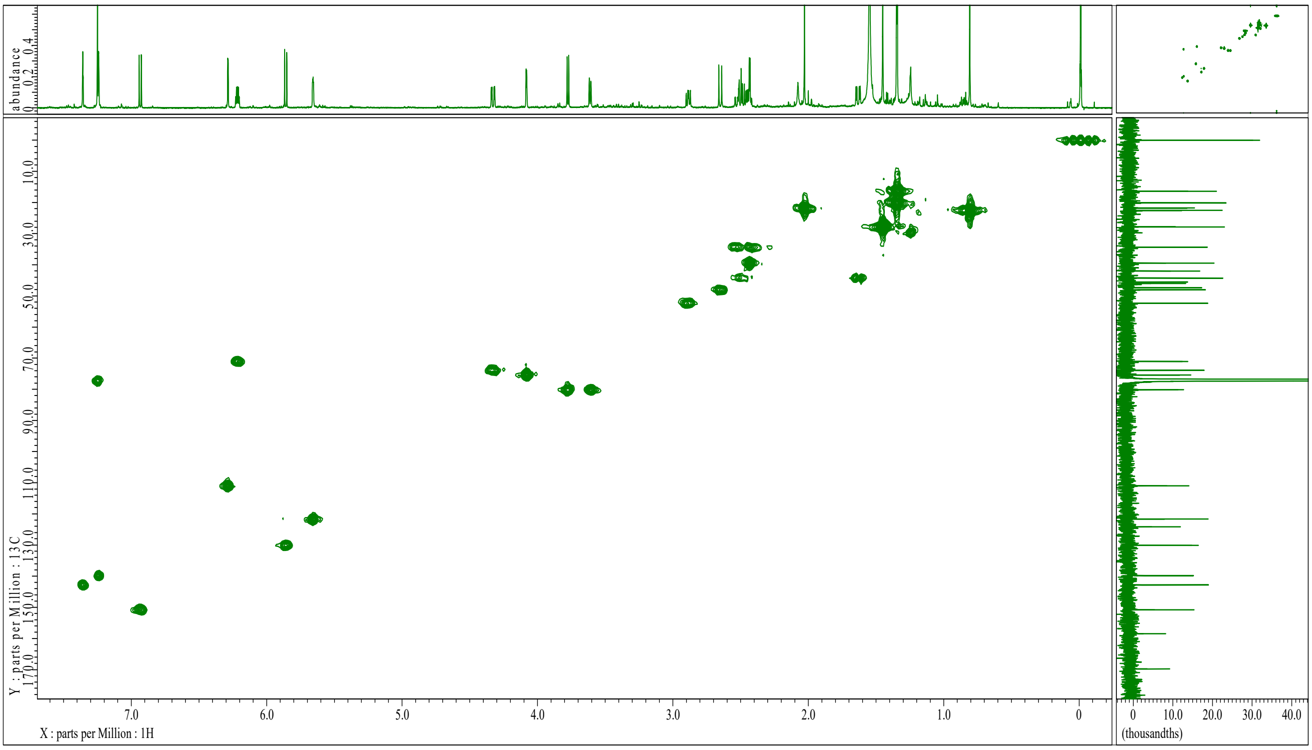


Figure S40. HMQC spectrum of compound **6** in $CDCl_3$ at 600MHz

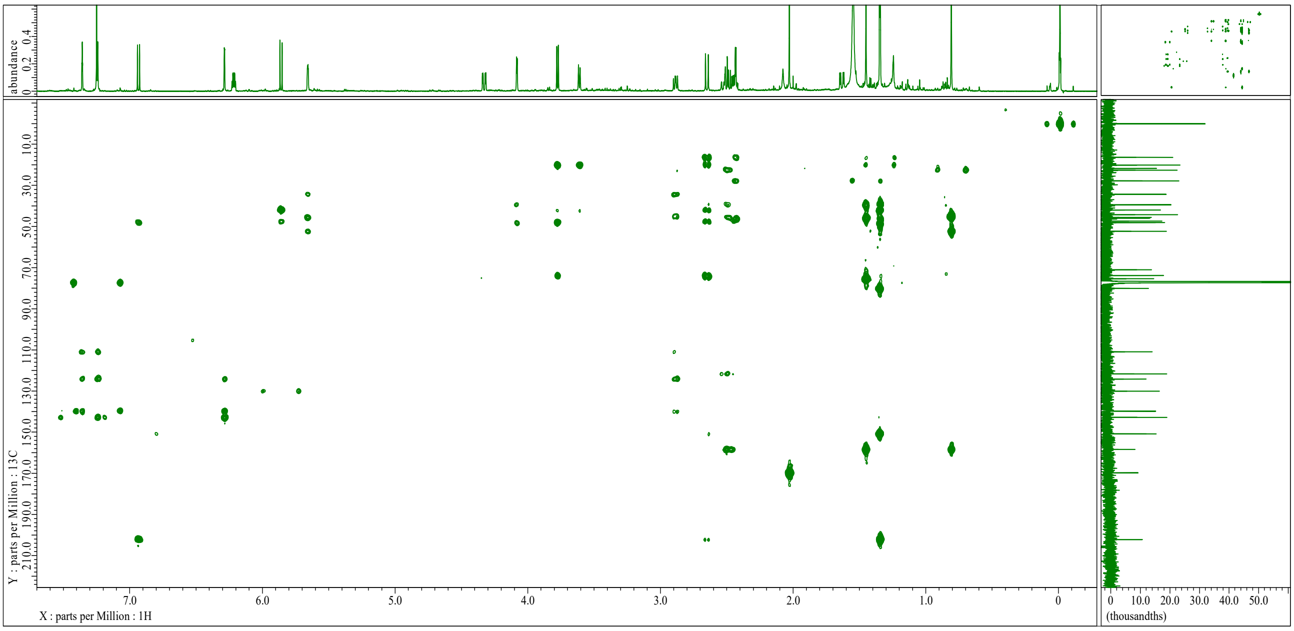


Figure S41. HMBC spectrum of compound **6** in $CDCl_3$ at 600MHz

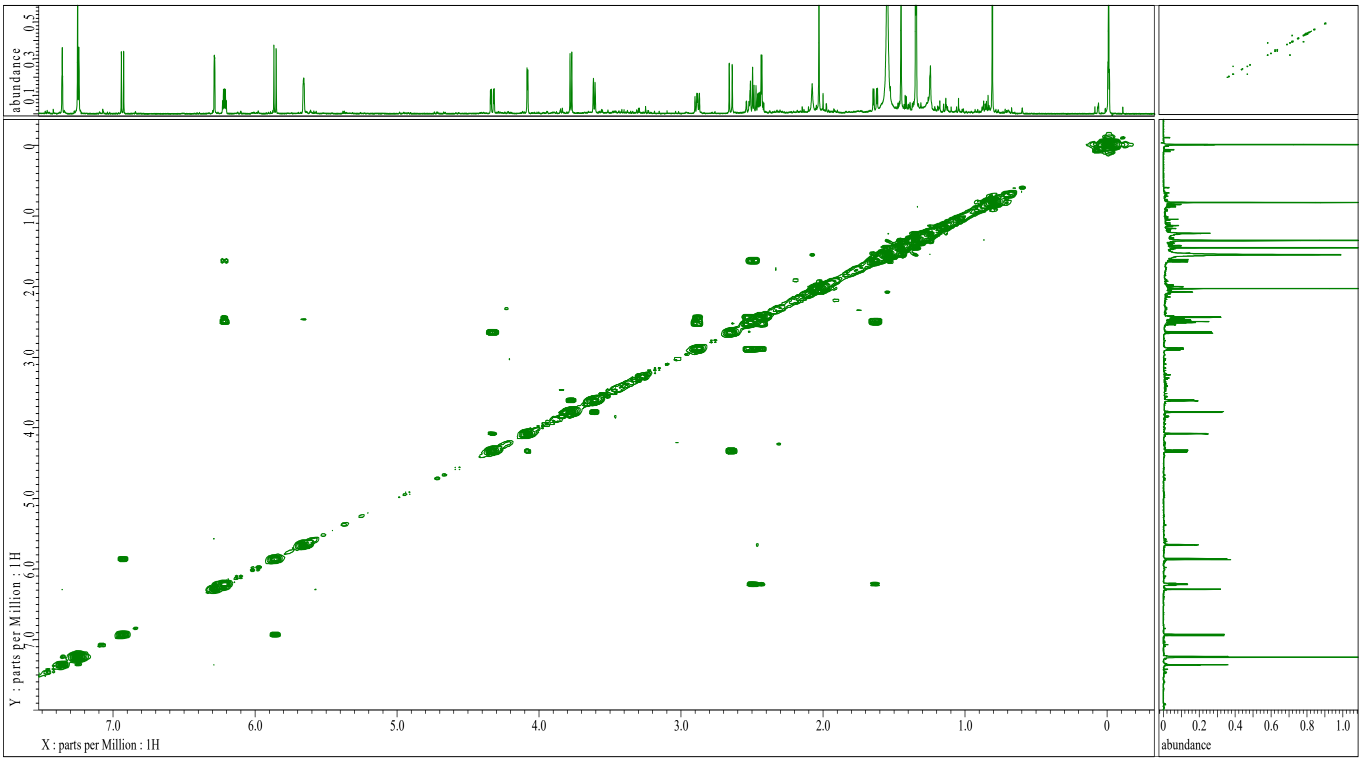


Figure S42. COSY spectrum of compound **6** in $CDCl_3$ at 600MHz

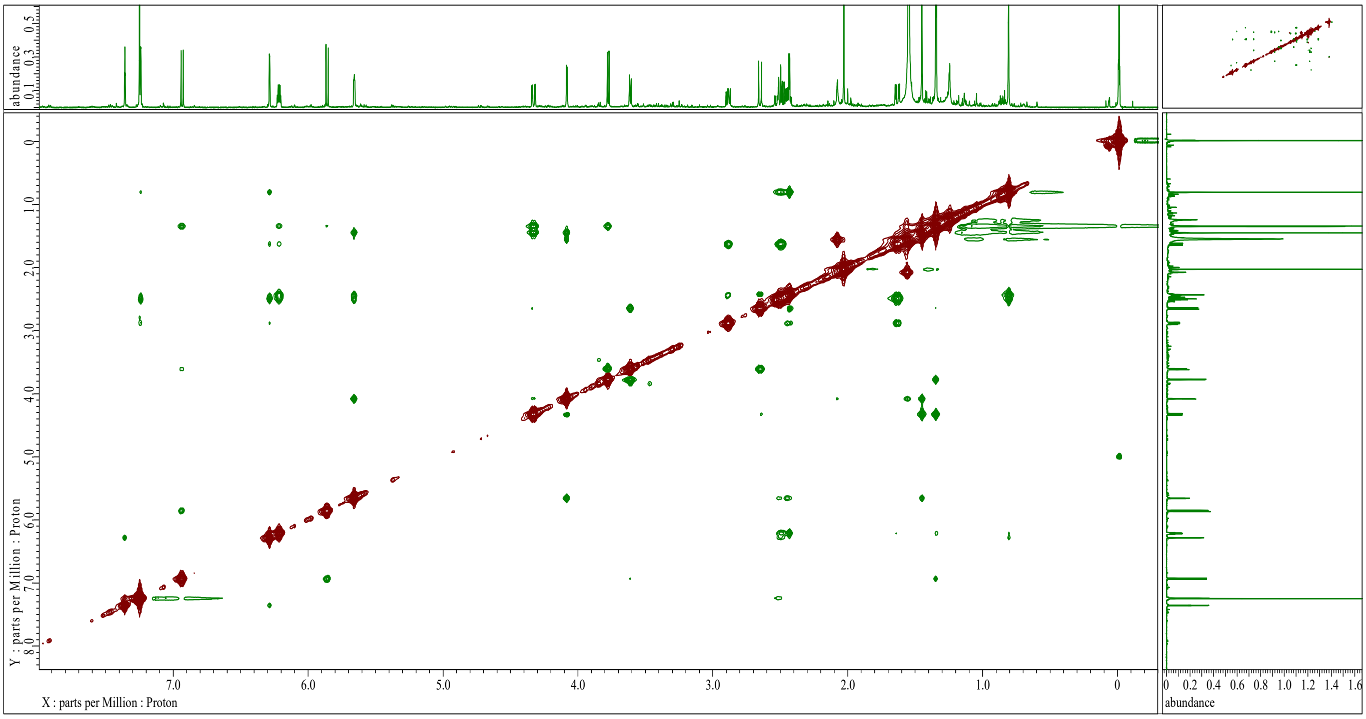


Figure S43. NOESY spectrum of compound **6** in $CDCl_3$ at 600MHz

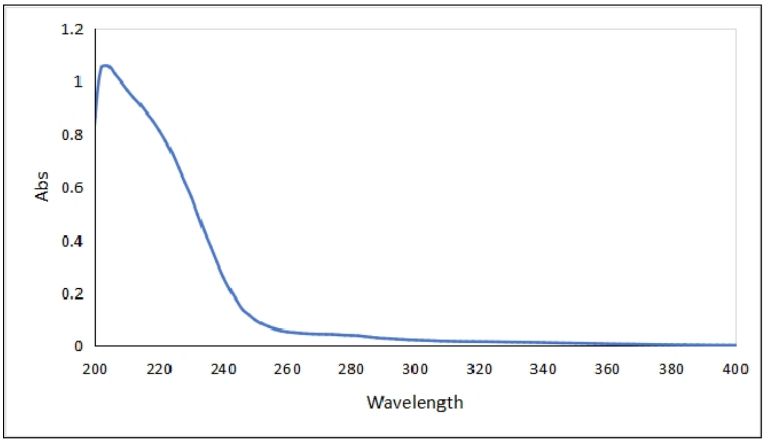


Figure S44. UV spectrum of compound **6** in MeOH

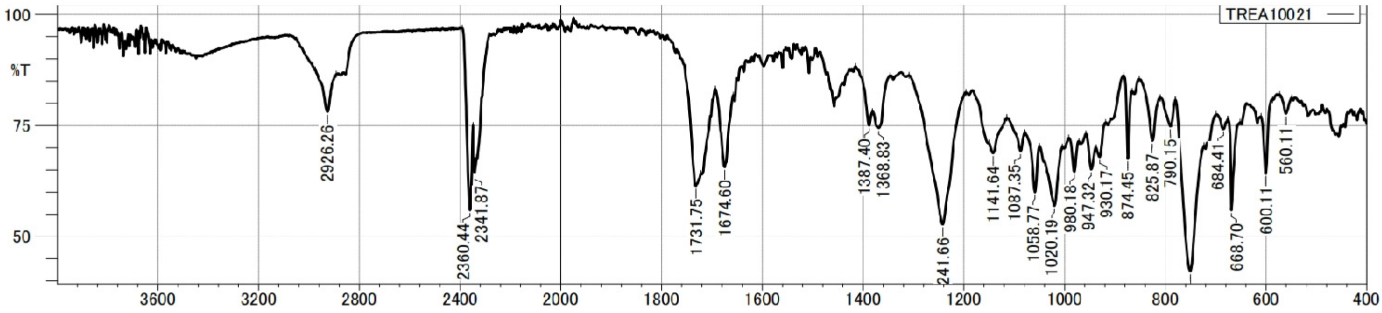


Figure S45. IR spectrum of compound **6**

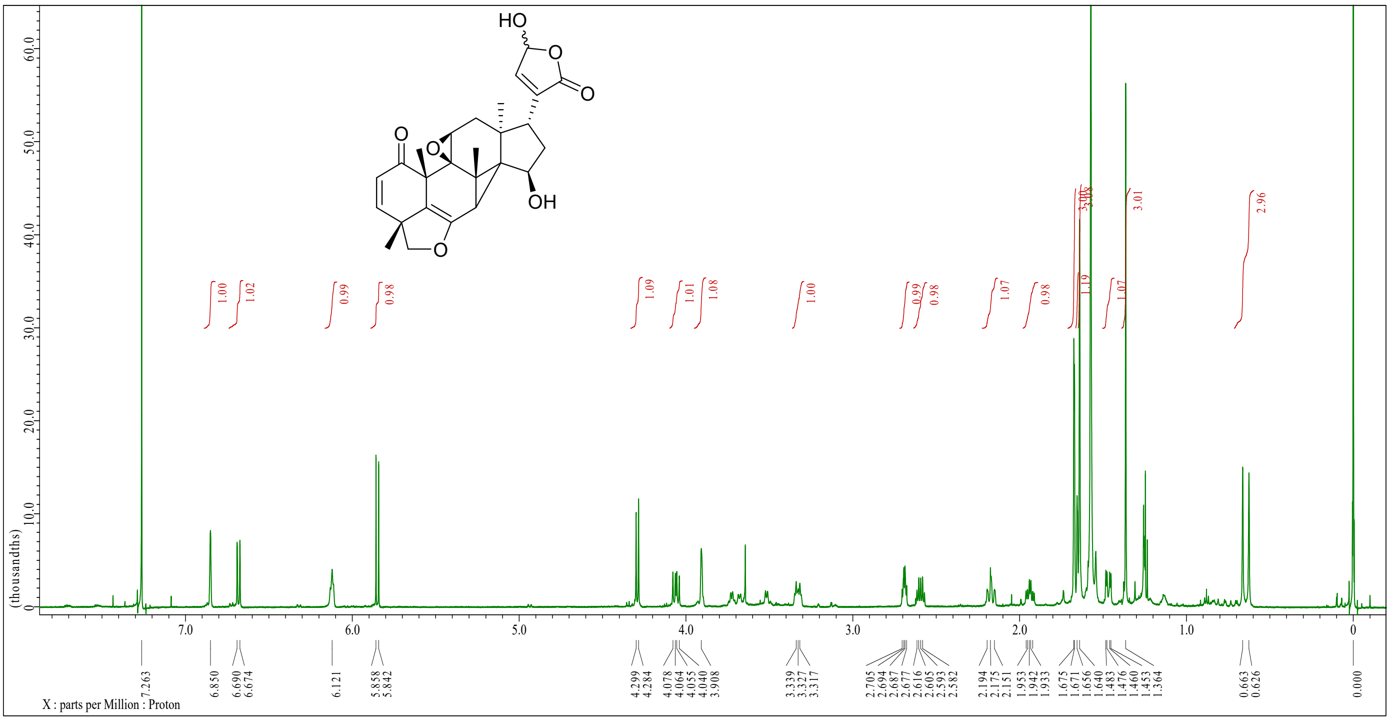

Figure S46. $^{1}$H NMR spectrum of compound **7** in $CDCl_3$ at 600MHz

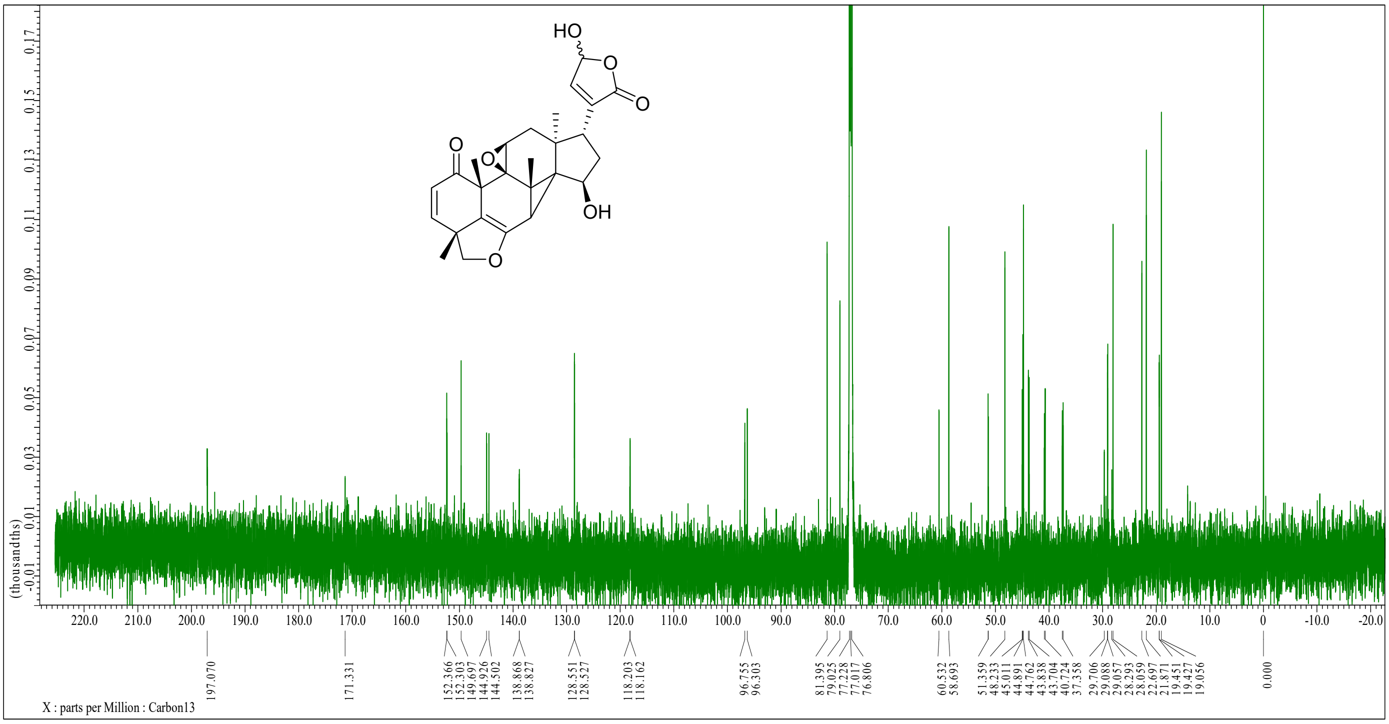

Figure S47. $^{13}$C NMR spectrum of compound **7** in $CDCl_3$ at 150MHz

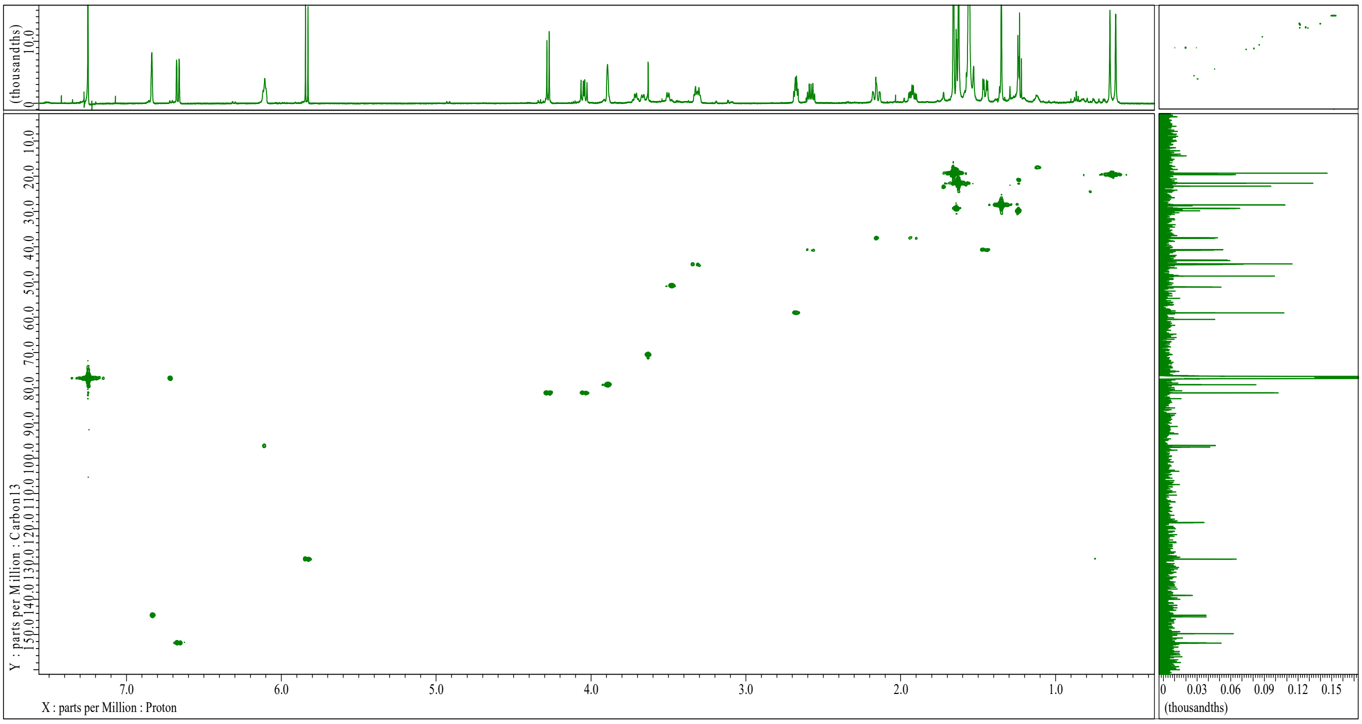

Figure S48. HMQC spectrum of compound **7** in $CDCl_3$ at 600MHz

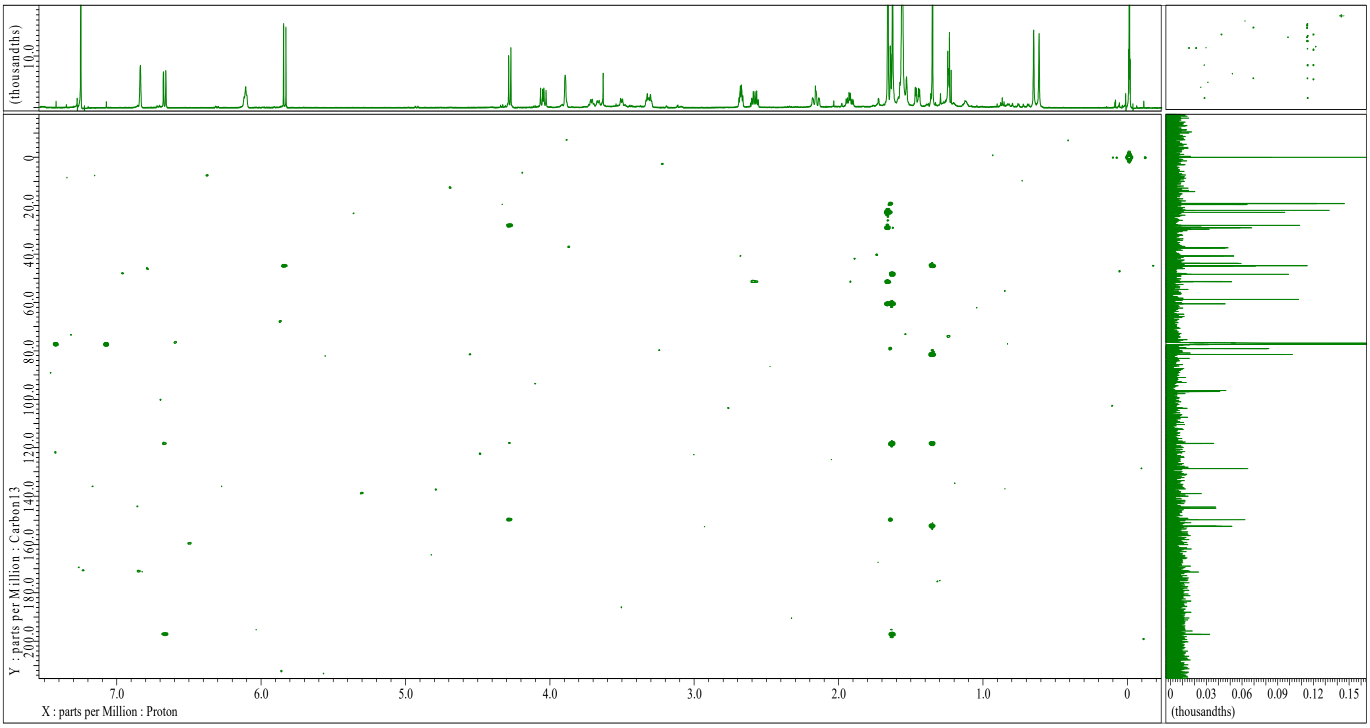

Figure S49. HMBC spectrum of compound **7** in $CDCl_3$ at 600MHz

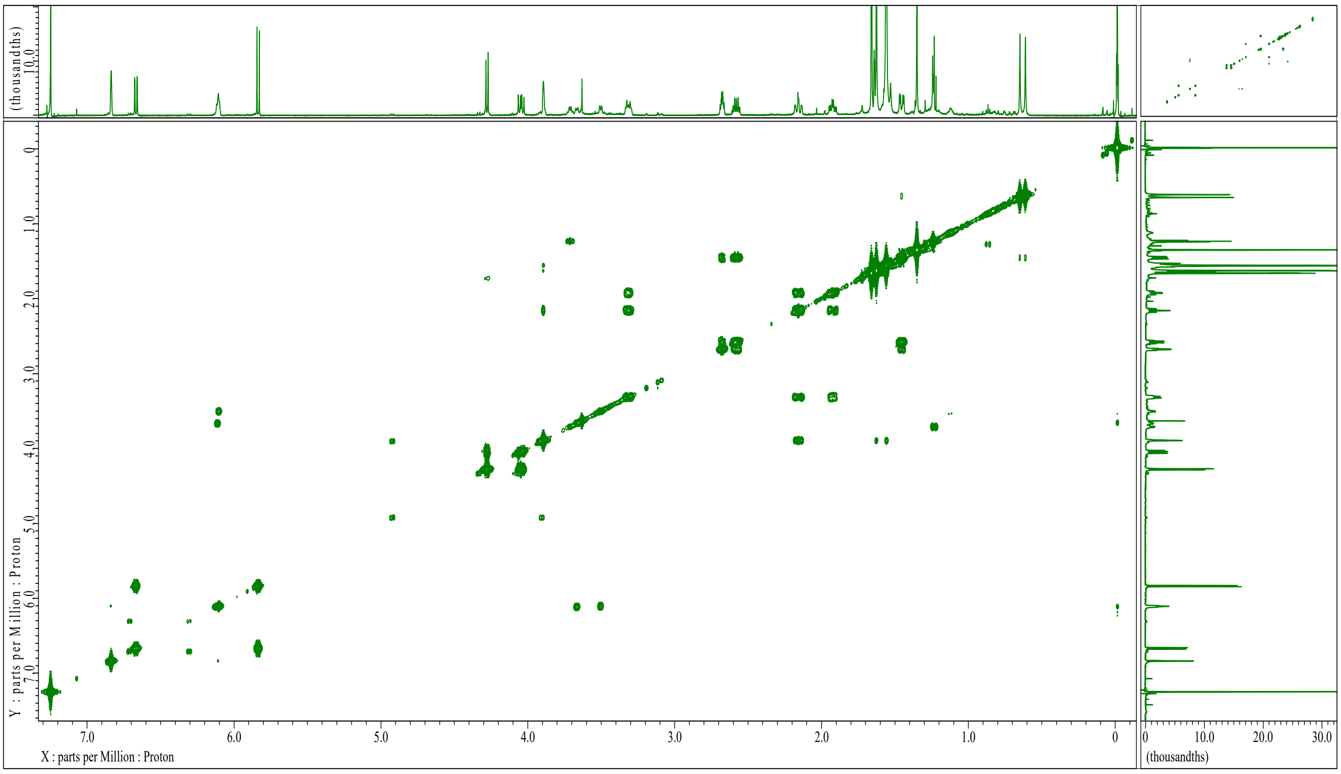


Figure S50. COSY spectrum of compound **7** in $CDCl_3$ at 600MHz

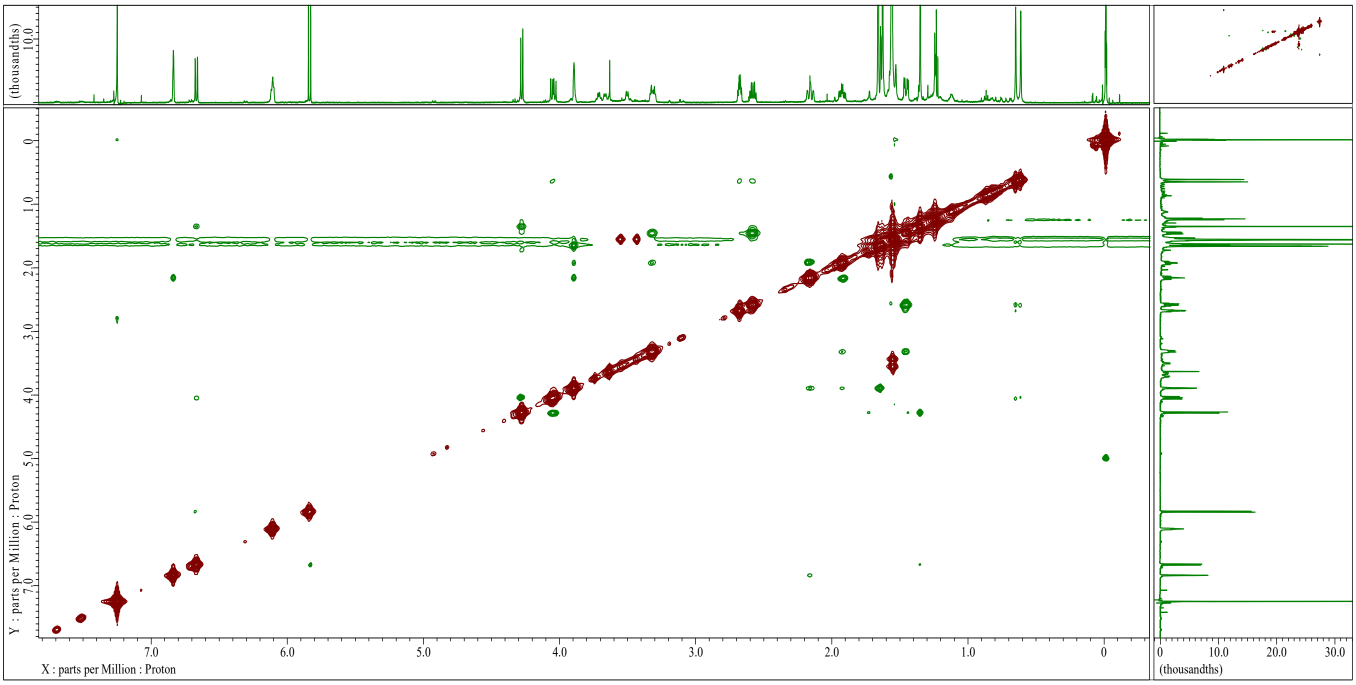


Figure S51. NOESY spectrum of compound **7** in $CDCl_3$ at 600MHz

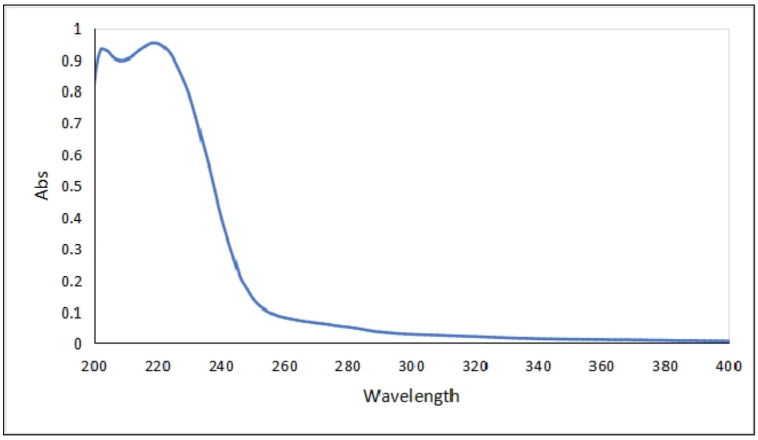


Figure S52. UV spectrum of compound **7** in MeOH

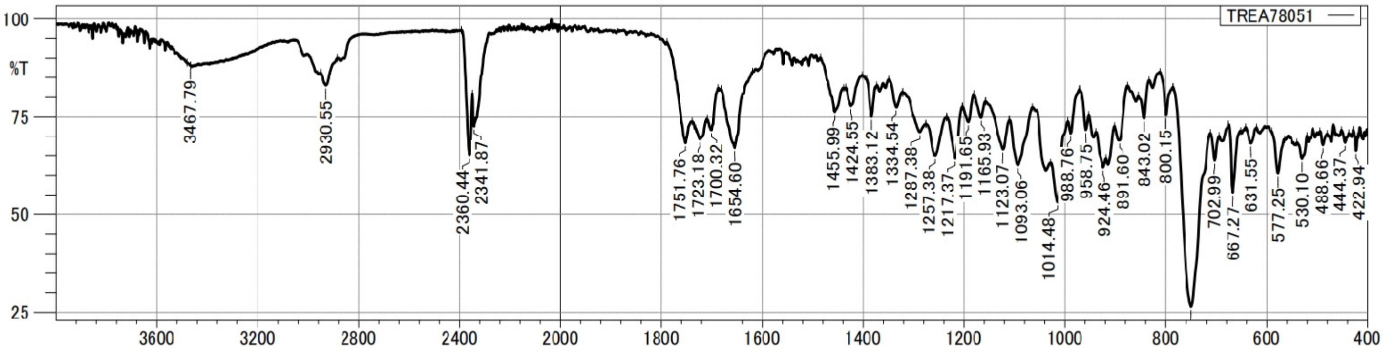


Figure S53. IR spectrum of compound **7**

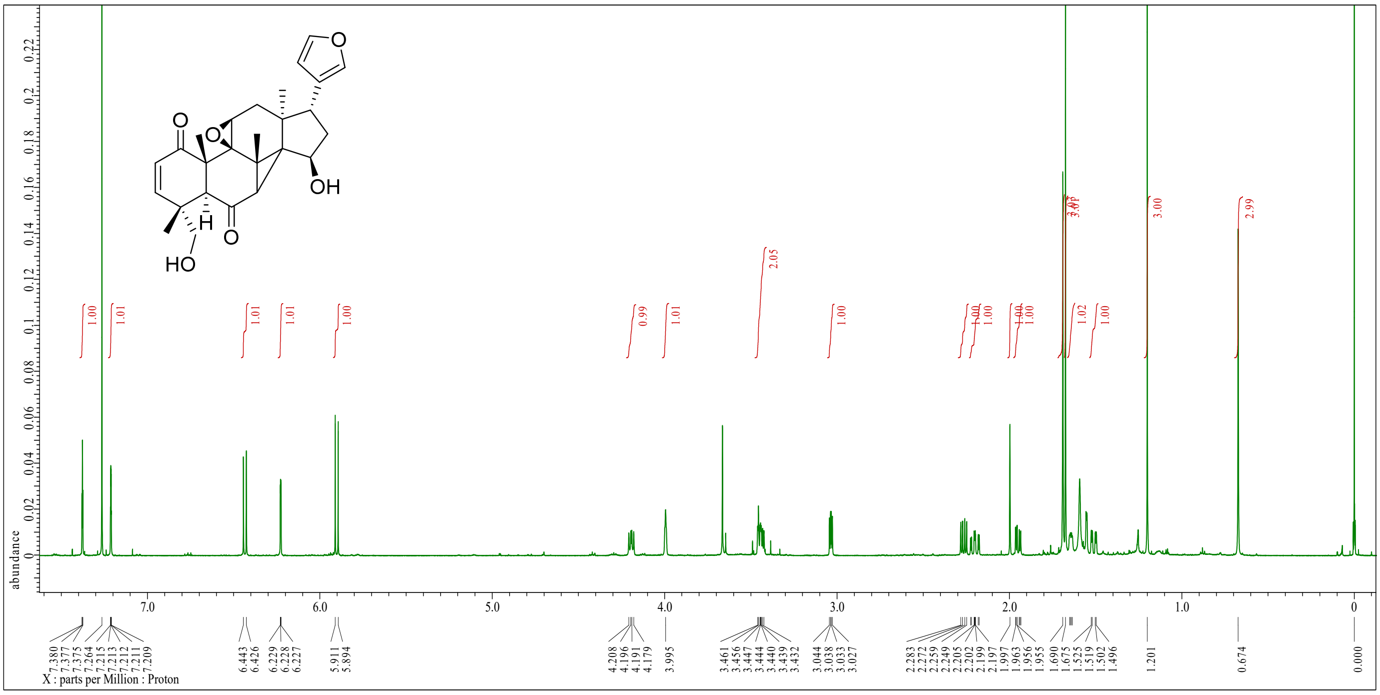

Figure S54. $^{1}$H NMR spectrum of compound **8** in $CDCl_3$ at 600MHz

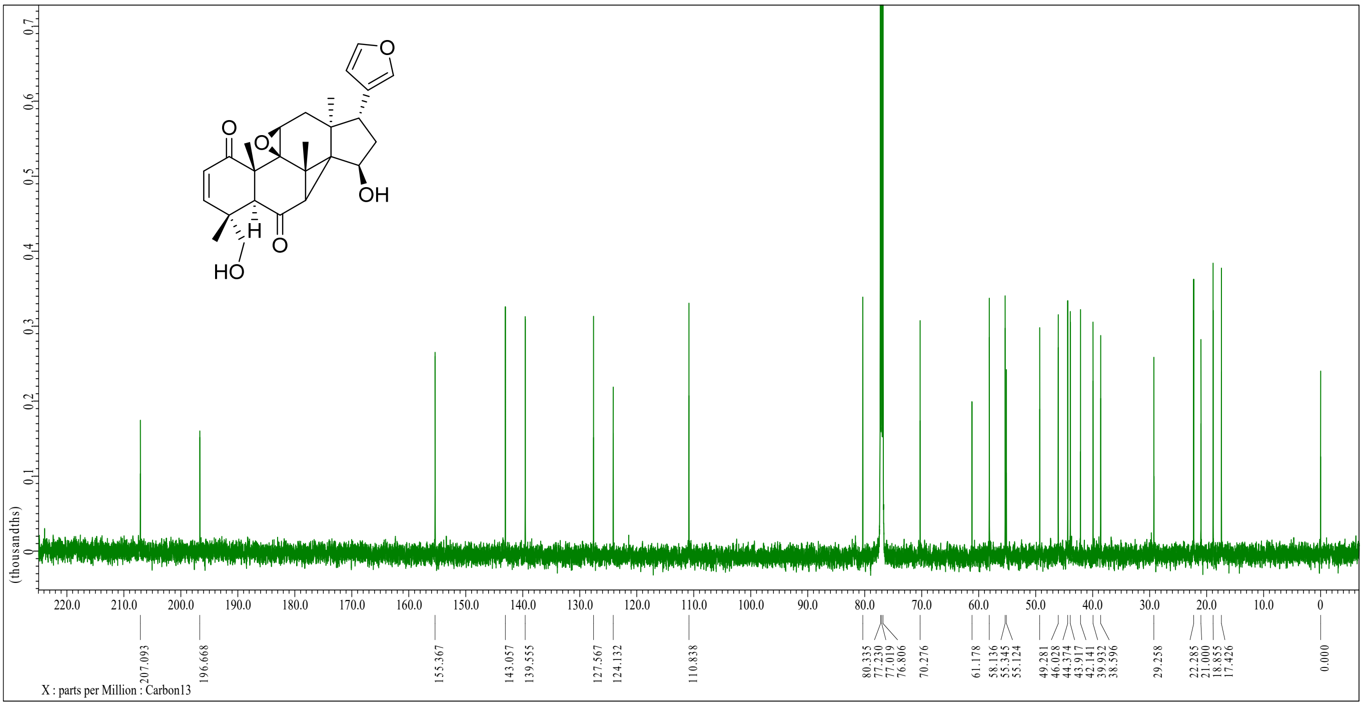

Figure S55.$^{13}$C NMR spectrum of compound **8** in $CDCl_3$ at 150MHz

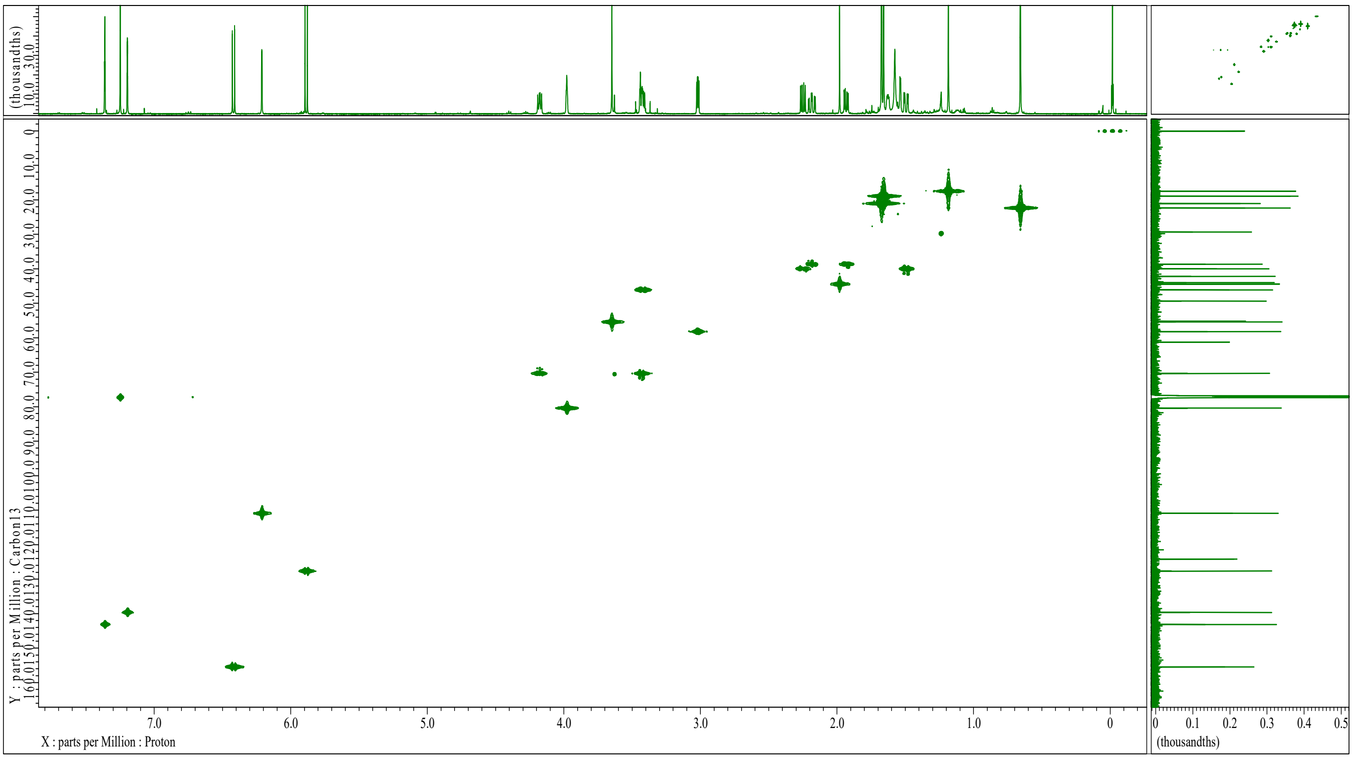


Figure S56. HMQC spectrum of compound **8** in $CDCl_3$ at 600MHz

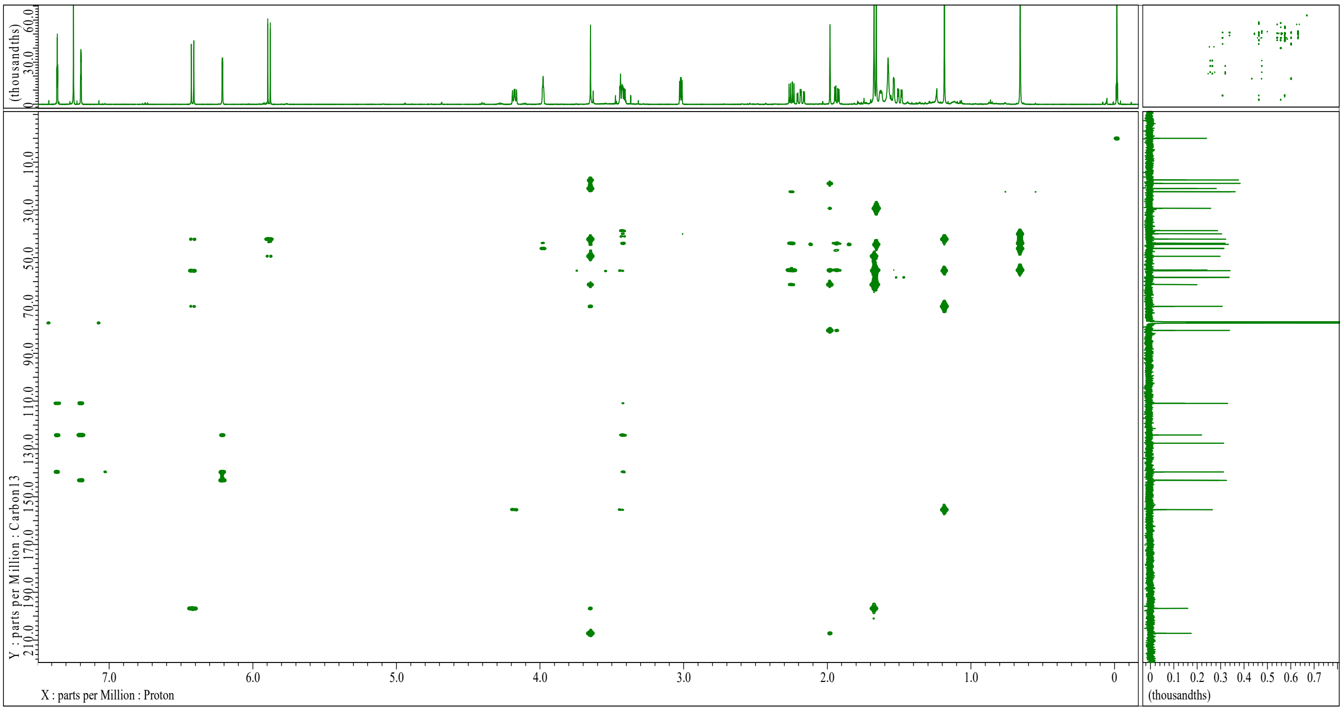


Figure S57. HMBC spectrum of compound **8** in $CDCl_3$ at 600MHz

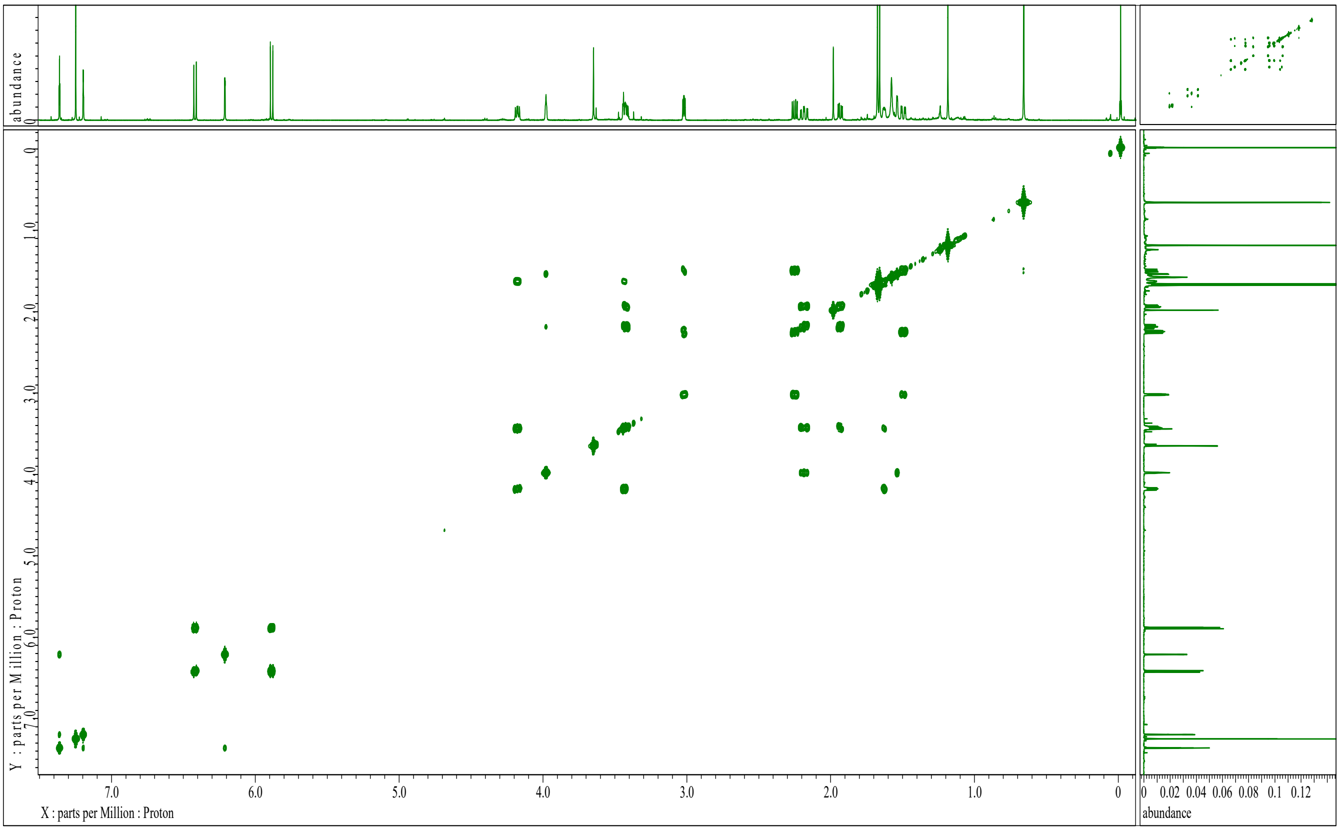


Figure S58. COSY spectrum of compound **8** in $CDCl_3$ at 600MHz

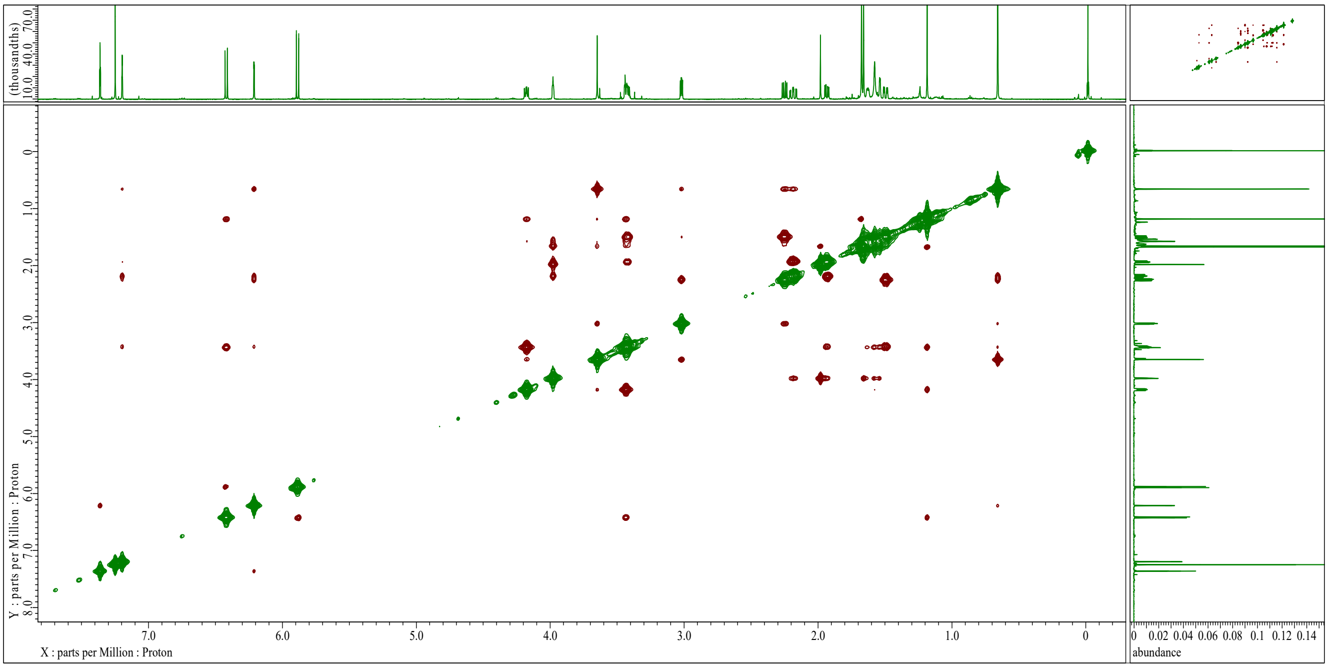


Figure S59. NOESY spectrum of compound **8** in $CDCl_3$ at 600MHz

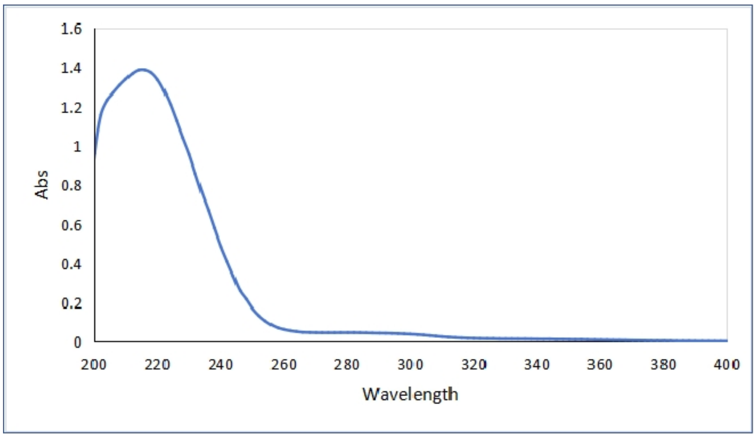


Figure S60. UV spectrum of compound **8** in MeOH

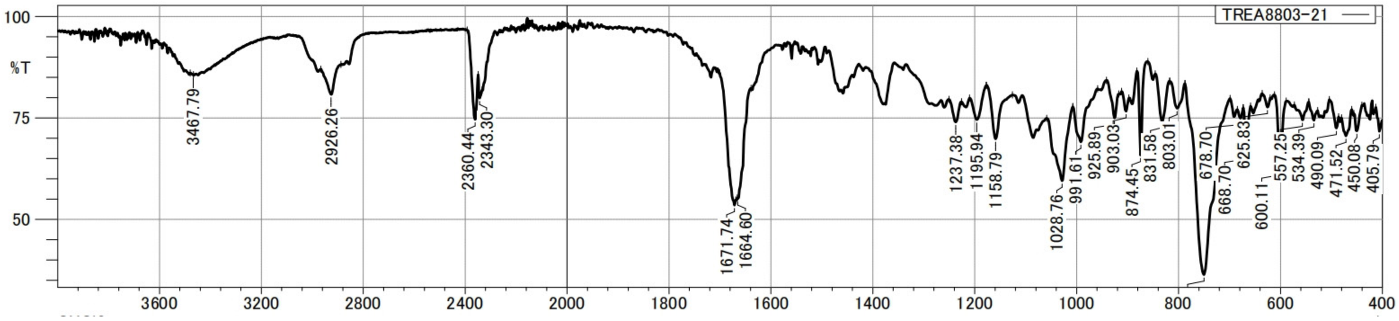


Figure S61. IR spectrum of compound **8**

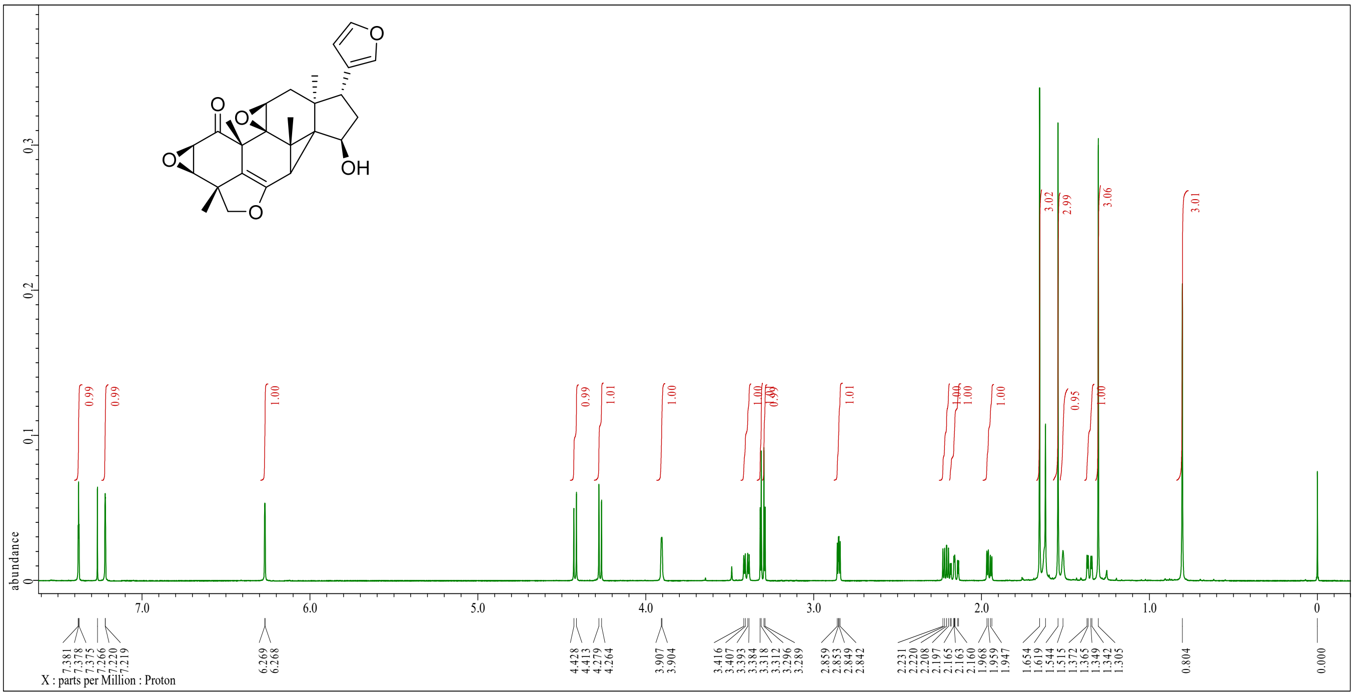

Figure S62. $^{1}$H NMR spectrum of compound **17** in $CDCl_3$ at 600 MHz

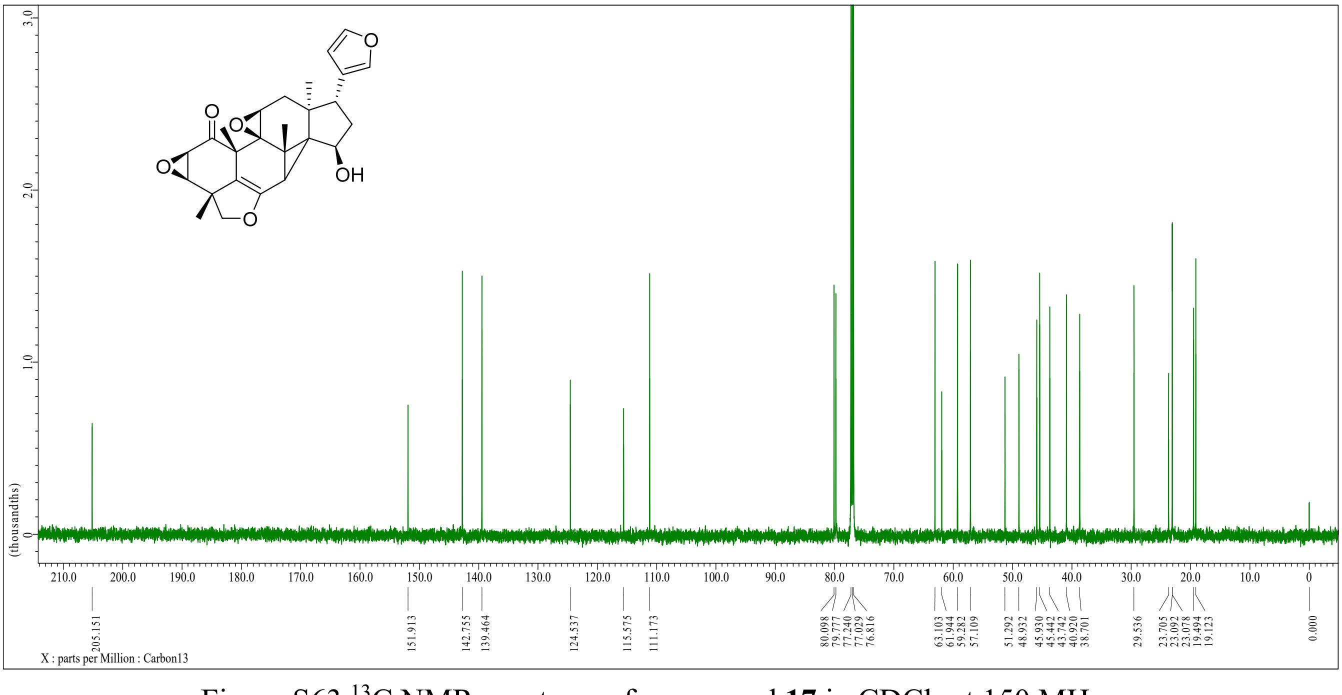

Figure S63.$^{13}$C NMR spectrum of compound **17** in $CDCl_3$ at 150 MHz

## LC/MS analysis of compound 1 and fraction 7.2

An authentic sample of compound **1** was diluted with acetonitrile/water (3:1) containing 0.1% formic acid to 10 µM. A concentration of the fraction 7.2 was also prepared to 10 µM with the same solvent, assuming an average molecular weight of compounds containing in the fraction as 500. The sample was subjected to LCMS-9030 (Shimadzu, Kyoto, Japan) connected with Candenza CX-C18 (2 x 50 mm, 3 µm, Imtakt, Kyoto, Japan). The mobile phases were solvent A: 0.1% formic acid in water, and solvent B: 0.1 % formic acid in acetonitrile. The gradient elution was performed as follows: 0–0.5 min, A90% /B5% ; 0.5–1.0 min, A90% /B10% B to A65% /B35% ; 1.0–5.0 min; A55% /B45%. The column temperature was maintained at 50°C, and the flow rate was 0.4 mL/min. The autosampler temperature was maintained at 4°C, and the injection volume was 3 µL. The sample was ionized by atmospheric pressure chemical ionization (APCI) and the data were obtained in the positive ion mode with a centroid format at *m/z* 100–1000 on a quadrupole time-of-flight (Q-TOF) mass spectrometer.

Total ion chromatogram (TIC)

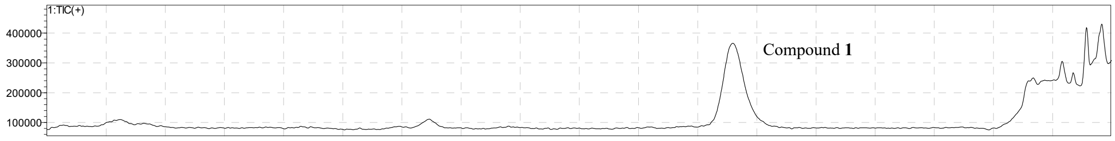


IC of 595.1979–595.2219

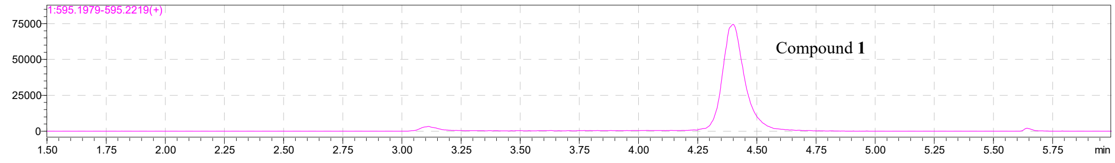


Mass spectrum at 4.4 min.

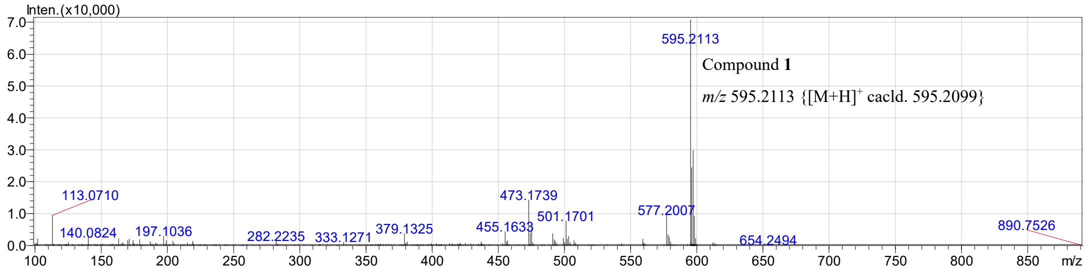


Figure S64. LC/MS analysis of compound **1**

TIC

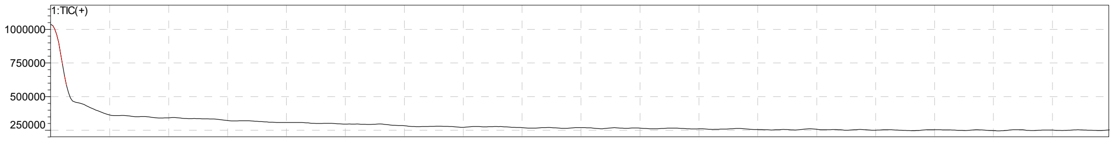

IC of 595.1979–595.2219

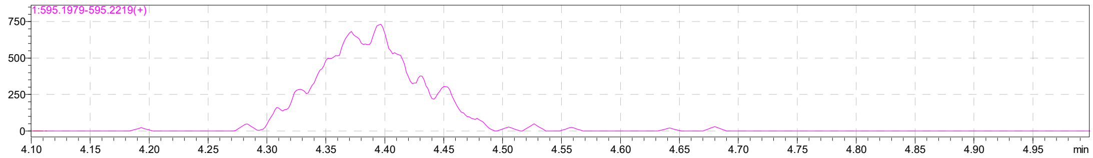

Mass spectrum at 4.4 min.

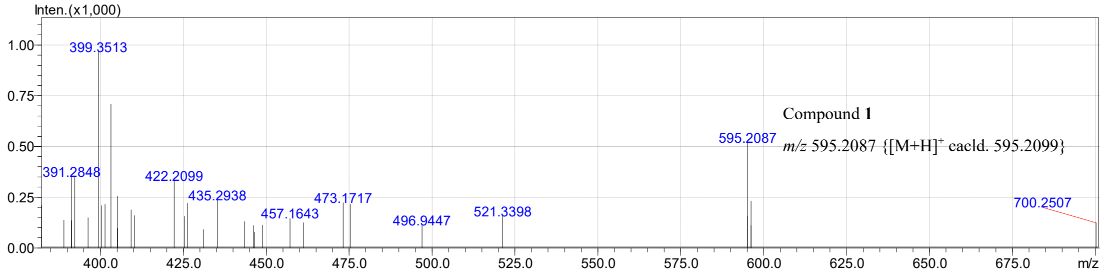


Figure S65. LC/MS analysis of fraction 7.2

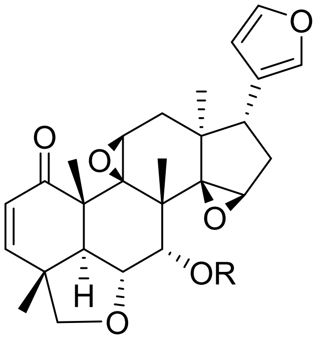

TS1 R= H

TS2 R=

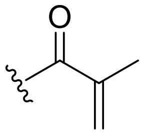

Figure S66. Structures of TS1 and TS2
